# Seed‐dispersing vertebrates and the abiotic environment shape functional diversity of the pantropical Annonaceae

**DOI:** 10.1111/nph.70113

**Published:** 2025-04-09

**Authors:** Andressa Cabral, Irene M. A. Bender, Thomas L. P. Couvreur, Søren Faurby, Oskar Hagen, Isabell Hensen, Ingolf Kühn, Carlos Rodrigues‐Vaz, Hervé Sauquet, Joseph A. Tobias, Renske E. Onstein

**Affiliations:** ^1^ German Centre for Integrative Biodiversity Research (iDiv) Halle – Jena – Leipzig Puschstrasse 4 Leipzig 04103 Germany; ^2^ Institute of Biology Leipzig University Leipzig 04103 Germany; ^3^ Instituto de Ecología Regional CONICET‐UNT Residencia Universitaria Horco Molle Ed. Las Cúpulas Yerba Buena 4107 Argentina; ^4^ DIADE, University of Montpellier, CIRAD, IRD 911 Ave d'Agropolis Montpellier 34394 France; ^5^ Department of Biological and Environmental Sciences University of Gothenburg Box 461 Göteborg SE 40530 Sweden; ^6^ Gothenburg Global Biodiversity Centre Box 461 Göteborg SE 40530 Sweden; ^7^ Martin Luther University Halle‐Wittenberg, Institute of Biology/Geobotany and Botanical Garden Große Steinstraße 79/80 Halle (Saale) 06108 Germany; ^8^ Department Community Ecology Helmholtz Centre for Environmental Research – UFZ Theodor‐Lieser‐Str. 4 Halle (Saale) 06120 Germany; ^9^ Institut de Systématique, Evolution, Biodiversité (ISYEB), Muséum National d'Histoire Naturelle‐CNRS‐SU‐EPHE‐UA Paris 75005 France; ^10^ National Herbarium of New South Wales (NSW), Botanic Gardens of Sydney Mount Annan NSW 2567 Australia; ^11^ Evolution and Ecology Research Centre, School of Biological, Earth and Environmental Sciences University of New South Wales Sydney NSW 2052 Australia; ^12^ Faculty of Natural Sciences, Department of Life Sciences (Silwood Park) Imperial College London London SL5 7PY UK; ^13^ Naturalis Biodiversity Center Darwinweg 2 Leiden 2333 CR the Netherlands

**Keywords:** frugivory, functional richness, macroecology, mutualism, taxonomic richness, trait matching, tropical rainforest

## Abstract

- Mutualistic interactions between fruiting plants and frugivorous animals are shaped by interaction‐relevant functional traits. However, it is unclear whether ‘trait matching’ underlies broad‐scale relationships in plant and frugivore species and their functional diversity.
- We integrated novel trait data and global occurrences for *c.* 1900 species in a major tropical plant family (Annonaceae) with data for 7607 bird and mammal species, including 1418 frugivores, alongside data on the abiotic environment. We applied structural equation models to evaluate the direct and indirect drivers of global and continental variation in frugivory‐related functional diversity in Annonaceae, and assessed frugivory‐exclusive drivers through comparisons with non‐frugivores.
- We show that global variation in Annonaceae frugivory‐related functional diversity is influenced by species richness (SRic) and trait matching with co‐occurring frugivorous mammals. Frugivorous birds and mammals indirectly influenced Annonaceae functional diversity at continental scales by affecting Annonaceae SRic. We found that climate, elevation, and seed dispersers jointly shaped Annonaceae diversity globally.
- Our results suggest that seed dispersal interactions with mammals are particularly important for shaping global variation in Annonaceae diversity, possibly through mutualistic co‐evolutionary dynamics. However, distinct effects of frugivores on Annonaceae diversity across biogeographical realms suggest that biogeography modulates how mutualistic interactions promote diversity.

## Introduction

The diversity of functional traits (i.e. characteristics of organisms that influence their fitness, growth, and survival; Violle *et al*., 2007) in assemblages is important for ecosystem multifunctionality, because co‐occurring species with contrasting trait values may exploit different resources, and thus increase resource utilization (Gross *et al*., 2017). In turn, multifunctionality may lead to diversity of ecosystem services, emphasizing the importance of understanding the origin and maintenance of functional diversity for conservation of ecosystems. Functional richness (i.e. trait space occupied by an assemblage, hereafter FRic; Mason *et al*., 2005; Villéger *et al*., 2008) has been increasingly used to understand how species interact with the environment and contribute to ecosystem functioning (e.g. Cadotte *et al*., 2011; Mason *et al*., 2013). Functional richness may differ between assemblages at different latitudes and biogeographical realms, as a result of differences in historical (e.g. paleoclimatic) and present‐day (e.g. current climate) processes shaping assemblages and traits (Lamanna *et al*., 2014; Svenning *et al*., 2015). The role of biotic interactions in shaping FRic across spatiotemporal scales, however, remains unclear and requires quantifying trait diversity in both interaction partners.

One such interaction type is the mutualism between fleshy‐fruited plants and frugivores (i.e. fruit‐eating and seed‐dispersing animals). This mutualism is prominent in tropical rainforests, where up to 90% of woody plants depend on frugivores for seed dispersal (Jordano, 2000). Both fruits and frugivores have evolved adaptive traits to facilitate interactions (Fleming & Kress, 2013). For example, frugivore gape width (and corresponding body size) constrains which fruit sizes can be swallowed, leading to the largest fruits generally being dispersed by the largest animals (Fleming & Kress, 2013; Galetti *et al*., 2013). This ‘trait matching’ (Dehling *et al*., 2014; Bender *et al*., 2018) may also explain the distribution of fruit sizes across broad‐scale global assemblages (Lim *et al*., 2020; McFadden *et al*., 2022; Wölke *et al*., 2023). Similarly, frugivores have evolved traits to facilitate the detection and handling of fruits (Fleming & Kress, 2013), such as primate color vision in relation to palm fruit colors (Onstein *et al*., 2020). This may explain the evolution of ‘fruit dispersal syndromes’, that is sets of matching frugivory‐related traits between plants and animals (Onstein *et al*., 2019, 2020; Valenta & Nevo, 2020). Frugivory‐related trait distributions and trait matching are, however, also influenced by abiotic variables, such as climatic conditions, productivity, and total plant or animal species richness (SRic; McFadden *et al*., 2022). Nevertheless, it remains unclear how frugivores, in addition to the abiotic environment, have influenced FRic of plants across broad‐scale assemblages and biogeographical regions (but see Albrecht *et al*., 2018).

**Fig. 1:**
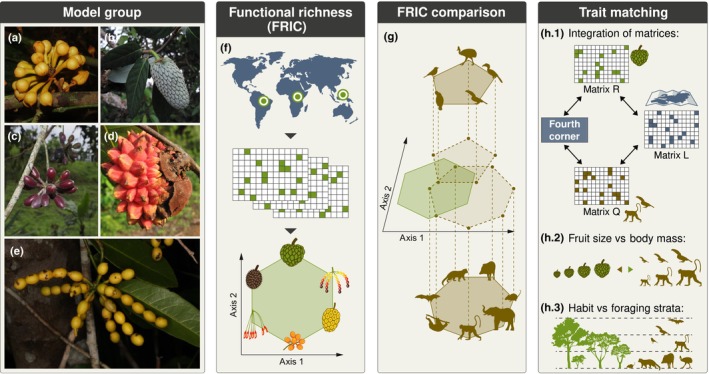
Conceptual framework of the quantitative trait‐based approach adopted in this study. (a–e) Examples of species and frugivory‐related functional traits of our model group Annonaceae (Table 1, Supporting Information Table S2): (a) *Anaxagorea phaeocarpa* Mart.; (b) *Annona hypoglauca* Mart.; (c) *Guatteria pichinchae* Maas & Westra; (d) *Letestudoxa bella* Pellegr.; (e) *Monanthotaxis* sp. (f) Inference of frugivory‐related functional trait spaces that reflect functional richness of co‐occurring Annonaceae species in an assemblage. (g) Comparison between frugivory‐related trait spaces of co‐occurring Annonaceae, frugivorous birds, and frugivorous mammals. Trait spaces were calculated separately for each assemblage and subsequently applied in our structural equation models. (h) Trait matching of Annonaceae, frugivorous birds, and frugivorous mammals across sites: (h.1) integration of plant (matrix R) and animals (matrix Q) trait with co‐occurrence (species interaction) data (matrix L) to calculate the ‘fourth corner’ – matching plant and frugivore traits; (h.2–h.3) examples of hypothesized matching plant and frugivore traits (Table S2); (h.2) fruit size with animal body size; and (h.3) plant habit with animal foraging strata. Photographs by: (a, c, d) T.L.P. Couvreur; (b) D. Sasaki; (e) L. Chatrou.

Here, we use a quantitative trait‐based approach to evaluate whether global variation in the frugivory‐related FRic of the pantropical custard apple plant family (Annonaceae) (Fig. 1a–f) can be explained by the (potential) mutualistic interaction with frugivorous birds and mammals (Fig. 1g,h). Annonaceae is the most species‐rich family within the Magnoliales (Chatrou *et al*., 2012), comprising *c*. 2500 predominantly tropical rainforest species (Couvreur *et al*., 2011; Chatrou *et al*., 2012; Erkens *et al*., 2022). Annonaceae have been well‐studied from taxonomic (e.g. Johnson & Murray, 2018), spatial (e.g. Erkens *et al*., 2022), biogeographic (e.g. Couvreur *et al*., 2011), and functional trait perspectives (e.g. Onstein *et al*., 2019; Xue *et al*., 2020), providing the basis for our study. Annonaceae show striking diversity of fruits (see Fig. 1a–e for examples), with sizes ranging from *c*. 0.25 cm (e.g. in certain apocarpic species such as *Greenwayodendron littorale* Lissambou, Dauby & Couvreur) up to 50 cm (e.g. in syncarpic species such as *Anonidium mannii* (Oliv.) Engl. & Diels) and brightly colored moniliform fruits with monocarps as beads in a necklace (e.g. a number of species within *Monanthotaxis* Baill.; see van Setten *et al*., 1992). This variation makes Annonaceae fruits attractive to an equally wide diversity of seed‐dispersing guilds of terrestrial vertebrates, especially birds, bats, primates, and other mammals (Supporting Information Table S1; Coates‐Estrada & Estrada, 1988; Kessler, 1993; McConkey *et al*., 2018; Onstein *et al*., 2019).

Due to reciprocal resource utilization and co‐evolutionary selective pressures from interacting partners, we hypothesize (H1) that frugivory‐related bird and mammal FRic (Fig. 1g) explains global variation in frugivory‐related Annonaceae FRic (Fig. 1f) across broad‐scale assemblages, even after accounting for the direct and indirect effects of SRic and the abiotic environment. Furthermore, we hypothesize (H2) that distinct biogeographical histories may have led to differences in frugivory‐related effects between realms. For example, we expect an overall stronger effect of frugivorous birds on Annonaceae diversity in the Neotropics and/or Asia‐Pacific realm than in the Afrotropics, due to the higher diversity and dominance of the frugivorous bird guild (compared with mammals, e.g. primates) in the respective realms (Kissling *et al*., 2009; Fleming & Kress, 2011). Finally, we hypothesize (H3) that individual functional traits of co‐occurring Annonaceae and frugivorous bird and mammal species are ‘matching’, underlying the association between frugivore and Annonaceae FRic (Fig. 1h; Table S2). For example, we expect that large‐fruited Annonaceae predominantly co‐occur with large‐bodied mammals, leading to a trait matching correlation between fruit sizes and animal body sizes.

## Materials and Methods

### Annonaceae data

Functional traits were selected based on their relevance for frugivory and seed dispersal (Table 1). Trait data were collected for each Annonaceae species by screening the literature (i.e. taxonomic monographs, floras and field guides, and online‐type collections such as JSTOR Global Plants, https://plants.jstor.org/). We focused measurements on the ‘dispersal unit’ (i.e. the ‘monocarp’ except in (pseudo‐)syncarpous fruits in which we measured the whole fruit). The data were documented in the PROTEUS database (Sauquet, 2019). When a range or multiple values were reported for a species, we used the species' mean trait value. We examined outliers and possibilities to reduce missing data through trait imputation from our initial dataset, which included 1895 species (spp.) and 109 genera (i.e. *c*. 77.5% and 98% of total species and genera, respectively). For further details, see Methods S1 and Fig. S1. From a total of 2448 Annonaceae species and 111 genera (following the World Checklist of Vascular Plants; Govaerts *et al*., 2021), our final database includes complete data on six frugivory‐related traits (fruit length, fruit width, fruit structure (apocarpous, (pseudo‐)syncarpous, moniliform), growth form (tree, shrub, liana), stipe length, and number of fruits; see Table 1 for trait descriptions) for 1274 spp. and 99 genera (i.e. *c*. 52% and 89% of total species and genera, respectively).

**Table 1:** Frugivory‐related Annonaceae traits used for functional richness estimates and their functionality for frugivory and seed dispersal.

Plant traits	Relevance for frugivory	References
Fruit length and width	There is a positive correlation between fruit size and bird gape size, beak volume, or bird or mammal body size in endozoochoric dispersal, due to physical constraints on the size of fruits and seeds that can be swallowed, ingested, and effectively dispersed	Wheelwright (1985); Jordano (2000); Chen & Moles (2015); Lim *et al*. (2020); McFadden *et al*. (2022); Wölke *et al*. (2023)
Fruit structure: apocarpic (i.e. fruit developed from a gynoecium with free carpels); (pseudo‐)syncarpic (i.e. fruit developed from partly connate carpels); moniliform (i.e. fruits constricted between the seeds)	Fruit type has been associated with dispersal by distinct frugivore guilds, such as moniliform with bird dispersal, and syncarpous fruits with dispersal by large‐bodied mammals (e.g. ‘megafauna’)	Janzen & Martin (1982); Gautier‐Hion *et al*. (1985); Onstein *et al*. (2019)
Growth form: tree (i.e. woody plant at least 5 m tall, typically with an unbranched main axis in its lower part); shrub (i.e. woody plant < 5 m tall, either lacking a distinct main axis or having branches that persist along the main axis nearly to its base); liana (i.e. woody climber)	Growth form affects the display of fruits across forest strata, and thus interaction with frugivore guilds that are restricted to particular strata (e.g. understory vs canopy frugivores). Vertical niche differentiation has been observed to persist even when food resources are available across forest strata (e.g. climber growth form). Plant height (approximated by growth form) positively relates to fruit size and dispersal distance, similarly as body mass approximates (home) range size in mammals, and wing shape (hand wing index) approximates flight efficiency and dispersal ability in birds	Carbone *et al*. (2005); Givnish (2010); Thomson *et al*. (2011); Onstein *et al*. (2017); Pires *et al*. (2018); Claramunt (2021); Thiel *et al*. (2023)
Stipe length (i.e. length of the stalk of a monocarp) and number of fruits (i.e. number of monocarps or (pseudo‐)syncarps that a single peduncle holds)	Many small rather than few large fruits, with many small, single‐seeded fruits, often positioned on long stipes as a result of spatial ‘packing’ of the monocarps, may increase dispersal opportunity as they can be swallowed and dispersed by a wider range of frugivores	Howe (1993); Godínez‐Alvarez *et al*. (2020)

For trait pairs used in the trait matching approach, see Supporting Information Table S2.

Distribution data for all Annonaceae species were assembled at the Taxonomic Databases Working Group (TDWG; https://www.tdwg.org/) level 1 (‘continents’) and level 3 (‘botanical countries’) (Govaerts *et al*., 2021). Botanical countries generally correspond to countries, but some larger countries are subdivided (e.g. the United States and Australia are divided into states; Indonesia is divided into each major island or archipelago). For 71 spp. that were missing from the TDWG data, we extracted occurrence coordinates from the Global Biodiversity Information Facility (GBIF, 2022) and other publications (Table S3). The GBIF coordinates were cleaned with R package coordinatecleaner (Zizka *et al*., 2019) and intersected with botanical country polygons to create a presence/absence matrix for each species in each botanical country (‘assemblage’, hereafter), using R package terra, by specifying the relations between polygons and coordinates as ‘coveredby’ and ‘overlaps’ (Hijmans *et al*., 2022). Species for which trait data were available contributed to *c*. 63% of the total spatial coverage of the family across botanical countries. This suggests that, although our dataset encompasses only about half of the total number of Annonaceae species, it captures a larger percentage of the spatial distribution of the family, particularly due to the presence of widespread species.

For a subset of 599 species (*c*. 25% of total), we also used previously cleaned and carefully curated occurrence records (Erkens *et al*., 2022) to define the presence/absence of species in assemblages at a more refined spatial resolution (i.e. cell size of 1 × 1 degree, *c*. 110 × 110 km, based on the Behrmann cylindrical equal‐area projection with standard parallels at 30°). This was performed to evaluate how spatial resolution affected our findings (for details see ‘Sensitivity analyses’ in the Materials and Methods section and Methods S1).

### Frugivore data

We focused on terrestrial birds and mammals because they are the most prominent frugivore guilds in tropical rainforests and the primary seed dispersers of Annonaceae (Coates‐Estrada & Estrada, 1988; Kessler, 1993; McConkey *et al*., 2018; Onstein *et al*., 2019). These groups are reported to account for *c*. 98% (birds: 32%; mammals: 66%) of the 297 unique pairwise interactions with 87 Annonaceae species (3.55% of the total 2448 Annonaceae species) in a global plant–frugivore meta‐network (Table S1). Bird and mammal taxonomy followed Tobias *et al*. (2022) and the International Union for Conservation of Nature (IUCN, https://www.iucnredlist.org, downloaded on 3 May 2022). We selected traits relevant for frugivory and seed dispersal. For birds, the traits included hand wing index and beak volume index (AVONET, Tobias *et al*., 2022; Table S2), foraging strata (EltonTraits, Wilman *et al*., 2014; Table S2), and percentage of fruits in the diet – reflecting the dependence of animals on fruit availability or frugivore–plant specialization (EltonTraits, Wilman *et al*., 2014). Beak volume index, calculated using beak length (from the tip along the culmen to the skull base), beak width, and beak depth (both measured at the anterior edge of the nostrils) following McFadden *et al*. (2022), simplifies the 3D geometry of avian beaks into an ellipsoid cone model to facilitate relative comparisons across species. For mammals, the traits included body mass (Phylacine 1.2, Faurby *et al*., 2018; Table S2), activity period (EltonTraits, Corlett, 2011; Wilman *et al*., 2014), foraging strata (EltonTraits, Wilman *et al*., 2014; Table S2), percentage of fruits in the diet (EltonTraits, Wilman *et al*., 2014), and color vision (Onstein *et al*., 2020).

Birds and mammals were classified into two functional groups according to the percentage of fruit in their diets: ‘frugivores’ (i.e. species with a predominantly fruit‐based diet, consuming fruits for at least 50% of their diet) and ‘non‐frugivores’ (i.e. species that (almost) never consume fruits, i.e. with 0% fruit in their diet, in contrast to those with seasonal or varying levels of fruit consumption). For justification of these classifications, see ‘Testing for frugivory‐exclusive drivers in the SEMs’ in the Materials and Methods section. For 90% bird species and 80% mammal species, diet data came from (reasonably) reliable sources (see data description in EltonTraits, Wilman *et al*., 2014); hence, we have high confidence in the classification. For the remaining species, diet was inferred based on genus‐ or family‐level information (Wilman *et al*., 2014).

Distribution range maps for extant native birds and mammals were obtained from BirdLife (http://datazone.birdlife.org/species/requestdis, downloaded on 14 February 2022) and IUCN (https://www.iucnredlist.org/resources/spatial‐data‐download, downloaded on 3 May 2022), respectively. These maps were intersected with the ‘botanical countries’ polygons to create a presence/absence matrix for each species in each assemblage. We focused on terrestrial birds and mammals (excluding species classified as marine or riverine) that co‐occur in assemblages with at least four species of Annonaceae (for justification of the cutoff value, see ‘Global assessment of frugivory‐related trait spaces’ in the Materials and Methods section). The included bird, mammal, and Annonaceae species are mostly confined to tropical rainforests.

Our final dataset for analysis was filtered to only include species with complete trait data, that is 1274 Annonaceae spp., 948 frugivorous bird spp., 3979 non‐frugivorous bird spp., 470 frugivorous mammal spp., and 2210 non‐frugivorous mammal spp. For more details on species coverage by family and functional group, see Table S4, and Table S5 for further details on frugivorous species not used in our framework, specifically those with a less strict fruit‐based diet.

### Abiotic environmental data

The abiotic environment can also influence plant and animal diversity (e.g. Sinnott‐Armstrong *et al*., 2021; McFadden *et al*., 2022). Hence, we assembled information on elevation (SRTM, Farr *et al*., 2007), net primary productivity (NPP in gC m^−2^/yr^−1^; MODIS, Justice *et al*., 1998), mean annual temperature, annual precipitation and precipitation seasonality, and total area occupied by a botanical country (area size, in km^2^) (Chelsa v.1.2, Karger *et al*., 2017; Lim *et al*., 2020). Mean values were calculated as averages of climatic raster cells that overlapped with each botanical country polygon. Ranges were calculated as the difference between the 95% and 5% quantiles (i.e. reducing the effect of outliers) in each assemblage (botanical country).

### Global assessment of frugivory‐related trait spaces

**Fig. 2:**
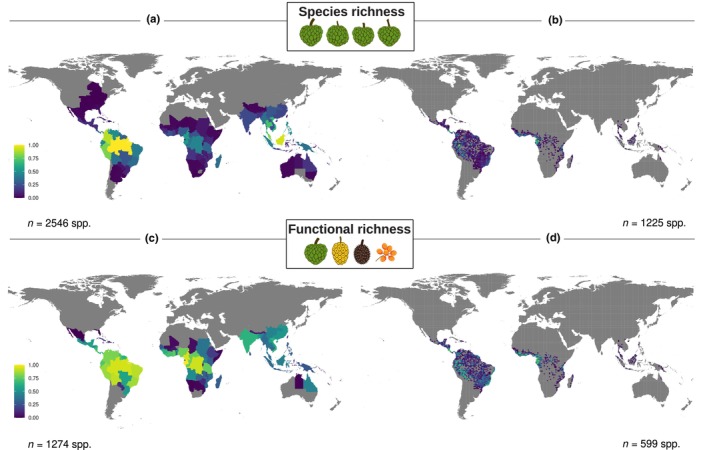
Global distribution of Annonaceae species and frugivory‐related functional richness (FRic). Species richness (a, b) and frugivory‐related FRic (c, d) are illustrated across botanical countries (a, c) and grid cells with *c*. 110‐km^2^ resolution (b, d). Only botanical countries and grid cells with at least four Annonaceae species, as used in the analyses, are illustrated. The minimum species count per assemblage was determined by the number of principal coordinate analysis (PCoA) axes used to calculate FRic, ensuring species counts exceeded the corresponding PCoA axes. Species richness ranges from 4 to 212 spp. (a) or from 4 to 53 spp. (b). All values were rescaled between 0 and 1 for visualization purposes. *n*, number of included Annonaceae species.

To estimate assemblage‐level functional diversity (i.e. FRic; Fig. 1f,g), multidimensional trait spaces were constructed for Annonaceae and for the two functional groups of birds and mammals separately (see ‘Frugivore data’ in the Materials and Methods section for further details). To optimize the representation of frugivory‐related functional diversity, we included as many relevant functional traits as feasible. Fruit width and the number of fruits were excluded due to their high Pearson's correlation with fruit length (*r* = 0.74) and stipe length (*r* = 0.69), respectively, as well as a higher proportion of missing data (Fig. S1). Additionally, the percentage of fruit in the diet was excluded due to its unequal variance between frugivores and non‐frugivores (zero inflation for non‐frugivores). Categorical traits were transformed into ‘dummy’ variables, whereby each trait state is represented by a single binary column (0 or 1). To accommodate for intraspecific variability (i.e. polymorphic traits within a species), we used a ‘fuzzy’ variable approach following De Bello *et al*. (2021b) whereby we coded the binary states such that the sum of the trait states per species equals 1 (i.e. 0.5 for trait state ‘0’, 0.5 for trait state ‘1’). In this way, a species can exhibit varying degrees of traits rather than fitting into a single category (1 or 0), thereby better capturing polymorphic traits. However, due to the lack of population‐level trait state frequency data, we used equal weights of trait states as a simplification. This avoids assuming dominance of one trait state over the other in the absence of explicit empirical evidence, while still acknowledging polymorphism. We emphasize, however, that this equal‐weighting approach assumes that trait states are equally prevalent within species, which may not align with biological reality in cases in which trait frequencies are unequal. Mammals with ‘polymorphic’ color vision (i.e. populations with a mix of tri‐ and dichromatic individuals; Jacobs, 1993) were scored as 0.5 trichromatic and 0.5 non‐trichromatic. Continuous traits were log‐transformed to improve normality, and subsequently scaled between 0 and 1 to achieve similar weights in the multitrait dissimilarity matrix. We used the ‘Gawdis’ distance (De Bello *et al*., 2021a) to calculate the dissimilarity between species pairs, as this method accommodates multiple types of variables (Pavoine *et al*., 2009). It adjusts trait weights based on their type (e.g. categorical vs continuous) by minimizing the differences in the correlation between individual trait dissimilarities and the multitrait dissimilarity. The multitrait dissimilarity matrix was used to perform a principal coordinate analysis (PCoA) using R package mfd (Magneville *et al*., 2022). The quality of PCoA spaces was evaluated by comparing the mean absolute deviation between the trait‐based distance and distance in the PCoA‐based space while increasing the number of principal components (i.e. ‘mad’ values, function ‘quality.fspaces’ in the R package mfd, Magneville *et al*., 2022). In this way, these ‘mad’ values indicate how many dimensions, that is PCoA axes, will maximize the quality of the functional space. To investigate the variation in frugivory‐related trait spaces globally, we calculated the functional volume of the convex hull occupied by each botanical country (FRic), using the clade‐specific multidimensional trait space provided by the PCoA. FRic calculations were based on the number of PCoA axes indicated by the ‘mad’ values. Specifically, we used two PCoA axes for frugivorous mammals, three for Annonaceae and non‐frugivorous mammals, and five for frugivorous and non‐frugivorous birds. Since these calculations can only be performed for assemblages with a greater number of species than the selected PCoA axes, we restricted FRic calculations to assemblages with species counts exceeding the corresponding number of PCoA axes. Therefore, we used botanical countries with a minimum of four Annonaceae and non‐frugivorous mammal species, three frugivorous mammal species, and six frugivorous and non‐frugivorous bird species. Finally, we combined the FRic/SRic datasets of Annonaceae with those for each functional group of birds and mammals, resulting in 101 botanical countries for the frugivore model and 109 for the non‐frugivore model (Table S6). In this way, we emphasize that our subsequent analyses are constrained specifically to the geographical zones where Annonaceae species are found (Fig. 2). As such, occurrences outside these zones have no impact on the estimated relationships, and our conclusions should be interpreted within this geographical scope.

### Global drivers of frugivory‐related Annonaceae functional richness

**Fig. 3:**
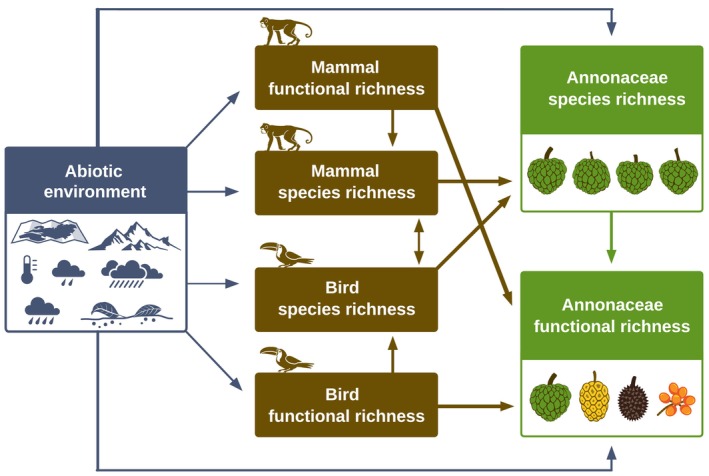
All hypothesized direct and indirect pathways among the predictor variables for Annonaceae species richness (SRic) and functional richness in the structural equation model. Nonsignificant relationships were gradually removed to obtain the optimal model fit, in which only significant paths (at *P* < 0.05) remained. A covariance parameter between bird and mammal SRic was included *a priori* in the base model due to their high Pearson's correlation in the main global model (*r* = 0.86). Model modification indices were evaluated, and when necessary, theoretically justified covariates were incorporated *a posteriori* to improve overall model fit.

Structural equation models (R package lavaan; Rosseel, 2012) were used to investigate the direct and indirect (cascading) effects on global assemblage‐level variation in Annonaceae FRic (H1). Our primary objective was to assess whether the FRic of frugivorous birds and mammals had a positive effect on Annonaceae FRic. Additionally, we examined whether Annonaceae SRic and various abiotic environmental factors (i.e. elevation range, NPP range, mean annual temperature, mean annual precipitation, mean precipitation seasonality, and area size) directly or indirectly (via bird or mammal SRic and/or FRic) explained Annonaceae FRic (Fig. 3). However, we emphasize that these biotic relationships are not assumed to be directly causal. The base model therefore reflects both direct environmental drivers and indirect ecological interactions that operate through these latent processes, which are not explicitly modeled but acknowledged as influential, for example niche differentiation and co‐diversification that likely drive patterns of Annonaceae SRic and FRic. Furthermore, our framework does not isolate the direct effect of animal FRic on Annonaceae FRic but instead accounts for cascading effects, in which animal SRic influences animal FRic, which in turn affects Annonaceae FRic, aligning with theoretical expectations. For each equation in the SEMs, we assessed the variance inflation factor, checked the normality and homogeneity of residuals, and examined extreme outliers that could potentially affect the results. To improve residual normality, we applied square root transformation to FRic of birds and mammals. For SRic, a count‐based response variable within the base model (Fig. 3), we compared residual distributions from generalized linear models with Poisson and negative binomial specifications against those from linear regression using SRic in its raw form and with transformations (i.e. logarithmic and square root). Square root transformation was selected for its conceptual simplicity and consistent approximation of normally distributed residuals. This was evidenced by visual inspection (*Q*–*Q* plots), low skewness (−0.29 to 0.34), and nonsignificant Shapiro–Wilk tests (*P* > 0.05) across equations in which SRic was a response variable in the global SEMs. To further account for remaining non‐normality and heteroscedasticity of residuals, we used a maximum likelihood estimation with robust SE when applicable (‘MLR’). This estimator adjusts SE and provides a scaled test statistic (Gana & Broc, 2019; Li, 2021). Predictor variables were rescaled to a range between 0 and 1 to facilitate comparison of their effects in the SEMs. Following the approach from Onstein *et al*. (2020), we first included all hypothesized direct and indirect pathways among the predictor variables (Fig. 3), and gradually removed paths with the least statistical significance until only significant paths (at *P* < 0.05) remained. A covariance parameter between bird and mammal SRic was included *a priori* due to their high Pearson's correlation in the main global model (*r* = 0.86). The model's modification indices were evaluated, and when necessary, theoretically justified *a posteriori* covariance parameters were incorporated to improve the overall model fit. Selection of the optimal model was based on the following criteria: *P*‐value of χ^2^ tests > 0.05, comparative fit index (CFI) > 0.90, and confidence interval of the root mean square error of approximation (RMSEA) < 0.05 or 0.08 (Schumacker & Lomax, 2004). From the optimal model, we extracted the standardized coefficients (Std.coef) and SE (Std.Err) for each significant path.

Simultaneous autoregressive error (SAR_err_) models were applied to evaluate the effect of spatial autocorrelation on our SEM outcomes. Following Onstein *et al*. (2020), we first fitted a (non‐spatial) ordinary least squares (OLS) (linear) regression model with the same set of predictor variables on Annonaceae FRic and SRic as recovered from the SEM. Because the standardized coefficients from the OLS models are equivalent to the path coefficients of the SEMs, they allow for a direct comparison of spatial autocorrelation between spatial and nonspatial models (Kissling & Carl, 2008). A spatial weight matrix was subsequently calculated using the minimum distance (i.e. increment = 1595.99 km) to connect each botanical country to at least one neighbor (R package spdep, Bivand *et al*., 2015) and applied to the SAR_err_ models (R package spatialreg, Bivand & Wong, 2018). As both OLS and SAR_err_ models produced similar predictor effects, we prioritized SEM standardized effects to facilitate interpretation, as spatial autocorrelation may also arise from biological processes rather than methodological biases. To determine whether spatial autocorrelation was present in the model residuals, we computed a spatial correlogram of Moran's *I* vs lag‐distance by applying the minimum distance to connect each botanical country to at least one neighbor using 999 permutations (R package ncf, Bjornstad & Bjornstad, 2022).

### Testing for frugivory‐exclusive drivers in the SEMs

Our SEMs focused on frugivores, that is animals with at least 50% fruit in their diet, because they are highly dependent on fruit as a major food source, and therefore, hypothetically, strongly shaped by trait matching with their food plants, including Annonaceae. However, to evaluate whether positive effects from frugivore FRic/Sric on Annonaceae FRic/SRic could have resulted from effects other than their frugivory‐related interactions (i.e. type I error), we repeated the global SEM with the ‘non‐frugivore’ bird and mammal datasets. We expected that the absence of frugivory‐related interactions should lead to a negative or absence of relationship between mammal or bird FRic/SRic and Annonaceae FRic/SRic in the SEMs, that is we checked whether we were able to correctly reject the false hypothesis of association. Accordingly, based on the overall SEM structure, we only consider positive biotic effects detected in the frugivore models that were negative or absent in the comparative non‐frugivore models as true ‘frugivory‐exclusive’ drivers (indicated with a star in our SEM figures).

### Simulations and sensitivity analyses

We used a null model approach to evaluate whether our findings deviated from a random expectation. Specifically, we repeated the global SEM after randomly shuffling Annonaceae FRic (with the corresponding SRic) across assemblages (botanical countries). Under this scenario, frugivore FRic/SRic should not affect Annonaceae FRic/SRic, because Annonaceae values are random, and thus independent from frugivory‐related interactions. By contrast, if similar effects of frugivore FRic/SRic on Annonaceae FRic/SRic would be detected in the null and empirical models, this would suggest that FRic/SRic of Annonaceae and frugivores could be correlated due to aspects other than their frugivory‐related interactions, and trait matching within assemblages would unlikely explain co‐occurrence patterns. Random shuffling (without replacement) was performed 1000 times using the R‐base ‘sample’ function. We assessed whether observed (empirical) effect sizes and *P*‐values of frugivore FRic/SRic on Annonaceae FRic/SRic fell outside the 95% distribution of simulated effect sizes and *P*‐values.

We used ‘botanical countries’ as our spatial unit of analysis because we are more confident of the presence/absence of Annonaceae species within these units, whereas at higher spatial resolution, false absences could bias outcomes. However, this means that we assume that co‐occurrence approximates frugivory‐related interactions of Annonaceae and frugivorous animals within these units, leading to co‐diversity patterns. To assess the validity of this assumption, we repeated the global SEM using a more refined spatial resolution for a subset of Annonaceae species and assessed whether results were consistent with findings at the botanical country level. See Methods S1 for further details.

### Biogeographical differences in drivers of Annonaceae functional richness

To evaluate whether biogeographical realms differed in the effects of frugivore FRic on Annonaceae FRic (H2), we repeated the SEM approach for subsets of botanical countries within the Afrotropics, Asia‐Pacific, or Neotropics. To this end, we assigned botanical countries and their respective continent (i.e. TDWG level 1) to biogeographic realms (i.e. ‘Africa’ to ‘Afrotropics’; ‘Southern America’ and ‘Northern America’ to ‘Neotropics’; ‘Asia‐Temperate’, ‘Asia‐Tropical’, ‘Australasia’, and ‘Pacific’ to ‘Asia‐Pacific’). RMSEA values were not considered for our continental‐scale SEMs due to their reduced sample size and degrees of freedom, which could potentially indicate a falsely poor model fit (Kenny *et al*., 2015).

### Trait matching in Annonaceae and frugivorous birds and mammals

To investigate which ‘trait matching’ in co‐occurring Annonaceae and frugivorous birds and mammals may underlie the functional diversity relationships detected in the global and realm models (H3), we used a modified version of the fourth‐corner analysis (Legendre *et al*., 1997; Dray & Legendre, 2008; Dehling *et al*., 2014). Specifically, we used the species interaction matrix (i.e. co‐occurring Annonaceae and frugivore species in botanical countries, plant × frugivore, matrix L), the Annonaceae trait data (Annonaceae species × traits, matrix R), and the frugivorous bird and mammal trait data (frugivore species × traits, matrix Q), to estimate the fourth corner: matching between Annonaceae and frugivore traits (Dehling *et al*., 2014; Fig. 1h). While FRic calculations included as many functional traits as feasible, the fourth‐corner analysis focused specifically on hypothesized trait relationships (Table S2). To evaluate the significance of trait matching, permutations were performed. Dray & Legendre (2008) and ter Braak *et al*. (2012) demonstrated that all previously suggested permutation models (i.e. Models 1–5) exhibited inflated type I error rates. A proposed solution was to combine the outputs from Model 2 (in which the rows, i.e. Annonaceae species of the interaction matrix are permuted) and Model 4 (in which the columns, i.e. frugivore species of the interaction matrix are permuted), and selecting the largest *P*‐values from these two permutation types (i.e. permutation model 6; Dray & Legendre, 2008; ter Braak *et al*., 2012). Following this approach, we applied permutation model 6 and specified a total of 1000 permutations. This analysis was performed using the function ‘fourthcorner’ in the R package ade4 (Dray *et al*., 2007).

## Results

### Pantropical covariation of frugivory‐related plant and animal functional richness

**Fig. 4:**
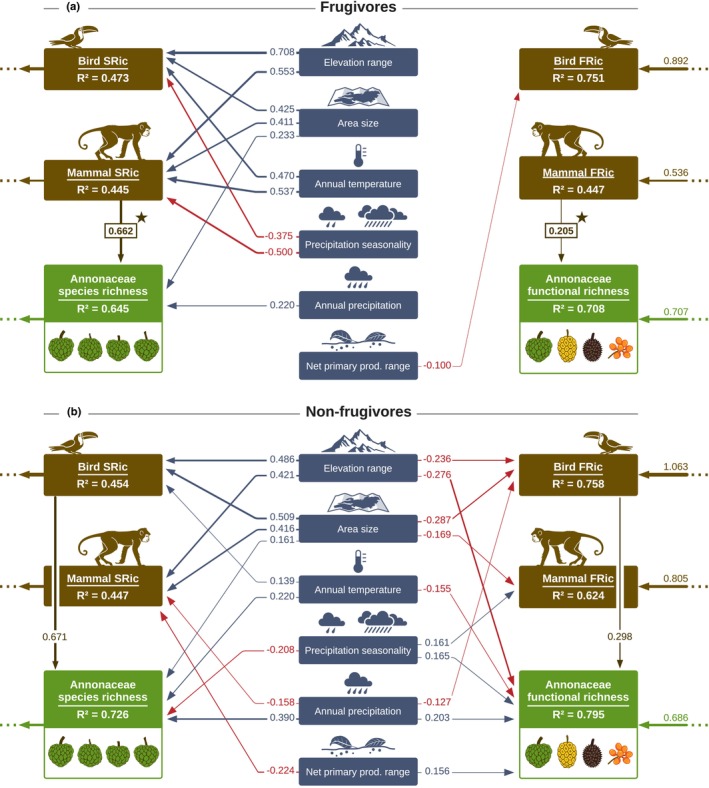
Global drivers of Annonaceae species richness and frugivory‐related functional richness. (a) Structural equation model (SEM) representing the standardized effects of predictor variables on Annonaceae, frugivorous bird, and frugivorous mammal species richness (SRic) and frugivory‐related functional richness (FRic) at the global scale. Bird and mammal SRic and FRic were based on a subset of frugivorous species with at least 50% of fruits in diet. (b) Comparative SEM with non‐frugivores. In this case, bird and mammal SRic and FRic were based on a subset of species with no fruit in their diet. For both (a) and (b), only statistically significant effects (standardized coefficients with *P* < 0.05) are shown. Standardized coefficients reflect the change in the response variable per unit change in the predictor, conditional on all other variables being held constant. Arrows indicate the direction of the effect, with arrow thickness proportional to effect strength. Red arrows represent negative effects. Positive biotic effects that were significant in the model with frugivores (a), but not significant in the model with non‐frugivores (b), are highlighted with a star. These suggest that the association between frugivores and Annonaceae is due to frugivory‐related interactions, rather than due to covariation between Annonaceae and frugivore SRic and/or FRic because of other factors. *R* ^2^ of response variables refers to the explained variation by all the predictor variables. Before model selection, FRic of birds and mammals, as well as SRic of Annonaceae, birds, and mammals, was square‐root‐transformed. A covariance parameter between bird and mammal SRic was included *a priori* in the base model. The model's modification indices were evaluated, and when necessary, *a posteriori* covariance parameters were incorporated to improve the overall model fit. These included the following: (a) bird FRic and mammal FRic, mammal FRic and mammal SRic, mammal FRic and Annonaceae SRic, Annonaceae FRic and bird FRic, bird FRic and mammal SRic, Annonaceae SRic and mammal SRic, and Annonaceae SRic and bird SRic; (b) none. Optimal model fit: (a) scaled *P*‐value of χ^2^ test = 0.083, robust comparative fit index (robust CFI) = 0.983 and robust root mean square error of approximation (robust RMSEA) = 0.067; (b) scaled *P*‐value of χ^2^ test = 0.174, robust CFI = 0.991 and robust RMSEA = 0.054.

Species richness and functional richness covaried globally for Annonaceae, frugivorous birds, and frugivorous mammals (Figs 2, S2). Consistent with H1, our SEM showed that Annonaceae FRic was directly and positively explained by frugivorous mammal FRic (Std.coef = 0.205, Std.Err = 0.042), but not bird FRic, after accounting for variation explained by the positive effect of Annonaceae SRic (Std.coef = 0.707, Std.Err = 0.068; Fig. 4a). Collectively, these factors explained 70.8% of the variation in Annonaceae FRic, which decreased to 68.2% after removing frugivore FRic and SRic from the base model and subsequently reapplying the stepwise removal of the least statistically significant paths in the whole model. Additionally, Annonaceae FRic was indirectly explained by mammal SRic via the positive effect on mammal FRic (Std.coef = 0.536, Std.Err = 0.095, Fig. 4a). Likewise, Annonaceae SRic was primarily positively explained by the SRic of frugivorous mammals (Std.coef = 0.662, Std.Err = 0.128; Fig. 4a), after accounting for the positive effects of area size (Std.coef = 0.233, Std.Err = 0.086; Fig. 4a) and annual precipitation (Std.coef = 0.220, Std.Err = 0.071, Fig. 4a). Our model explained 64.5% of the global variation in Annonaceae SRic, which decreased to 43.7% when we considered only abiotic predictors in the base model. With the exception of NPP range and annual precipitation, all abiotic variables also had an indirect effect on Annonaceae SRic via effects on mammal SRic (Fig. 4a).

The SEM including non‐frugivores (Fig. 4b) showed a positive association between Annonaceae FRic and bird FRic (Std.coef = 0.298, Std.Err = 0.065; Fig. 4b), but not with mammal FRic. Furthermore, Annonaceae SRic was also positively associated with the SRic of non‐frugivorous birds (Std.coef = 0.671, Std.Err = 0.080; Fig. 4b), but not with the SRic of non‐frugivorous mammals. This suggests that Annonaceae and frugivorous bird SRic may match due to non‐frugivory‐related processes, whereas frugivory is likely the primary driver of matching between Annonaceae FRic/SRic and mammal FRic/SRic, respectively (highlighted with a star in the SEMs; Fig. 4a).

Moran's *I* values indicated minimal spatial autocorrelation in model residuals (Fig. S3), possibly because the spatial autocorrelation structures of the predictor variables match that of the response variable. Furthermore, after accounting for spatial autocorrelation, our SAR_err_ models indicated mostly the same significant effects with similar strengths on Annonaceae FRic and Annonaceae SRic as in the SEM (Table S7).

### Effects of frugivorous mammals on Annonaceae diversity are not driven by stochasticity or spatial resolution

Results from the null model simulations suggest that the effect of frugivorous mammal FRic on Annonaceae FRic was not due to purely stochastic FRic distributions across botanical countries: Observed effect sizes strongly deviated from the mean and 95% distribution of simulated effect sizes, and *c.* 94% of the simulated effects were nonsignificant (*P* > 0.05; Fig. S4a,c). Additionally, the effects of frugivorous mammal SRic on Annonaceae SRic were no longer significant under our null model approach (i.e. > 94.5% of the effects with *P* > 0.05, and empirical effect sizes strongly deviated from the mean and 95% distribution of simulated effect sizes; Fig. S4b,d).

Biotic effects detected in the main model (Fig. 4a) were supported by the analysis with a more refined spatial resolution (Fig. S5a), with a similar effect of mammal SRic on Annonaceae SRic (Std.coef = 0.241, Std.Err = 0.022; Fig. S5a) and of mammal FRic on Annonaceae FRic (Std.coef = 0.030, Std.Err = 0.019; Fig. S5a). These effects were not observed in the models with non‐frugivore species (Fig. S5b). Additionally, the null models indicated that the effects of frugivorous mammals were not driven by purely stochastic FRic/SRic distributions across grid cells (Fig. S6).

### Biogeographical differences in drivers of Annonaceae functional richness

**Fig. 5:**
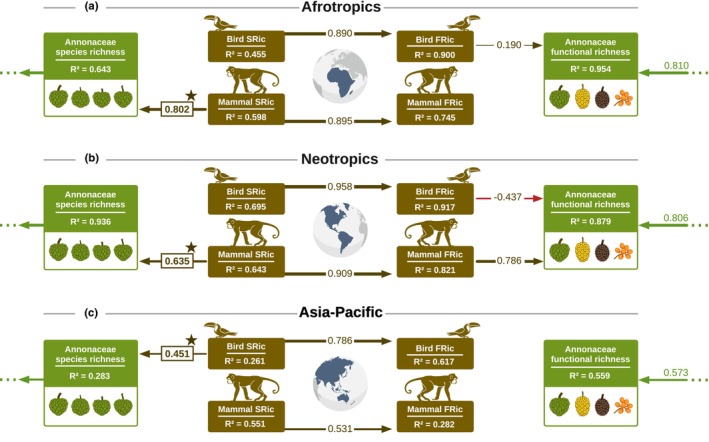
Drivers of Annonaceae species richness and frugivory‐related functional richness in the different biogeographical realms. Structural equation models (SEMs) representing the standardized effects of Annonaceae, frugivorous bird and frugivorous mammal species richness (SRic) and frugivory‐related functional richness (FRic) at continental scales, that is Afrotropics (a), Neotropics (b), and Asia‐Pacific (c). Abiotic effects were omitted to avoid visual redundancy with the global model, but see Figs S7(a), S8(a), and S9(a) for complete models. Bird and mammal SRic and FRic were based on a subset of frugivorous species with at least 50% of fruits in their diet. Only statistically significant effects (standardized coefficients with *P* < 0.05) are shown. Standardized coefficients reflect the change in the response variable per unit change in the predictor, conditional on all other variables being held constant. Arrows indicate the direction of the effect, with arrow thickness proportional to effect strength. Red arrows represent negative effects. Positive biotic effects that were significant in the models with frugivores, but not significant in the models with non‐frugivores (Figs S7b, S8b, S9b), are highlighted with a star. These suggest that the association between frugivores and Annonaceae is due to frugivory‐related interactions, rather than due to covariation between Annonaceae and frugivore SRic and/or FRic because of other factors. *R* ^2^ of response variables refers to the explained variation by all the predictor variables. Before model selection, FRic of birds and mammals, as well as SRic of Annonaceae, birds, and mammals, was square‐root‐transformed. A covariance parameter between bird and mammal SRic was included *a priori* in the base model. The model's modification indices were evaluated, and when necessary, *a posteriori* covariance parameters were incorporated to improve the overall model fit. These included the following: (a) none; (b) bird FRic and mammal FRic, bird FRic and Annonaceae SRic, and mammal FRic and Annonaceae SRic; (c) mammal FRic and Annonaceae SRic. Optimal model fit: (a) *P*‐value of χ^2^ test = 0.304, comparative fit index (CFI) = 0.991; (b) *P*‐value of χ^2^ test = 0.214, CFI = 0.990; (c) *P*‐value of χ^2^ test = 0.259, CFI = 0.976.

Consistent with H2, biogeographical realms differed in the effects of frugivores on Annonaceae FRic, with 95.4% (Afrotropics), 87.9% (Neotropics), and 55.9% (Asia‐Pacific) variation in Annonaceae FRic explained by the predictor variables (Figs 5, S7a, S8a, S9a).

In the Afrotropics, in contrast to the global model, there was a positive effect of frugivorous bird FRic (Std.coef = 0.190, Std.Err = 0.048) on Annonaceae FRic, but no effect of mammal FRic (Figs 5a, S7). In addition, we detected a negative effect from elevation range (Std.coef = −0.151, Std.Err = 0.069) and a positive effect of annual precipitation (Std.coef = 0.120, Std.Err = 0.036) on Annonaceae FRic (Fig. S7). Similar to the global model, Annonaceae FRic was strongly and positively influenced by Annonaceae SRic (Std.coef = 0.810, Std.Err = 0.046; Figs 5a, S7), which, in turn, was strongly and positively affected by the SRic of frugivorous mammals (Std.coef = 0.802, Std.Err = 0.111; Figs 5a, S7), but not birds. We did not detect any direct abiotic drivers of Annonaceae SRic in the Afrotropics.

In the Neotropics, similar to the global model, there was a strong and positive effect of frugivorous mammal FRic on Annonaceae FRic (Std.coef = 0.786, Std.Err = 0.125; Figs 5b, S8). However, we detected a negative effect of frugivorous bird FRic on Annonaceae FRic (Std.coef = −0.437, Std.Err = 0.138; Figs 5b, S8). In addition, Annonaceae FRic was positively explained by NPP range (Std.coef = 0.205, Std.Err = 0.082) and negatively by elevation range (Std.coef = −0.261, Std.Err = 0.087) (Fig. S8). Finally, Annonaceae FRic was positively influenced by Annonaceae SRic (Std.coef = 0.806, Std.Err = 0.084), which, in turn, was directly and positively affected by mammal SRic (Std.coef = 0.635, Std.Err = 0.059), area size (Std.coef = 0.279, Std.Err = 0.065), annual precipitation (Std.coef = 0.206, Std.Err = 0.054), annual temperature (Std.coef = 0.233, Std.Err = 0.114), and elevation range (Std.coef = 0.286, Std.Err = 0.082) (Figs 5b, S8).

In the Asia‐Pacific region, there were no direct effects of frugivores on Annonaceae FRic (Figs 5c, S9). However, we detected a strong positive effect of Annonaceae SRic (Std.coef = 0.573, Std.Err = 0.083) and precipitation seasonality (Std.coef = 0.410, Std.Err = 0.089), and a negative effect of elevation range (Std.coef = −0.264, Std.Err = 0.067) on Annonaceae FRic (Figs 5c, S9). Furthermore, Annonaceae SRic was strongly and positively affected by SRic from frugivorous birds (Std.coef = 0.451, Std.Err = 0.220; Figs 5c, S9), but not mammals. Finally, we detected a negative effect from elevation range (Std.coef = −0.436, Std.Err = 0.099) on Annonaceae SRic (Fig. S9).

Models that included non‐frugivores within biogeographical realms (Figs S7b, S8b, S9b) showed similar biotic effects on Annonaceae FRic as the frugivore models (Figs 5, S7a, S8a, S9a). However, the positive effects from mammal SRic on Annonaceae SRic in the Afrotropics and Neotropics, and from bird SRic on Annonaceae SRic in the Asia‐Pacific region, were exclusive to models including frugivorous species. This suggests that frugivory‐related interactions are important drivers of the association between Annonaceae SRic and frugivore SRic within biogeographical realms (highlighted with a star in the SEMs; Figs 5, S7a, S8a, S9a).

### Matching of Annonaceae and frugivore traits

The fourth‐corner analysis evidenced significant relationships (at *P* < 0.05) between frugivory‐related traits of Annonaceae and frugivorous mammals globally (Table S8), supporting the functional diversity association in the SEM (Fig. 4). Specifically, we detected significant associations between mammal body mass and fruit length and between mammal body mass and growth forms globally (Table S8). As we did not detect significant frugivore–Annonaceae FRic associations within biogeographical realms, no trait matching analyses were carried out for the individual realms.

## Discussion

Functional richness is recognized as a key component in ecosystem functioning, and it is well known that the abiotic environment shapes this trait diversity in plants (Song *et al*., 2014; McFadden *et al*., 2022). However, here, we illustrate the important role of biotic interactions – that is mutualistic interactions between fleshy‐fruited plants and frugivorous seed‐dispersing animals – in shaping FRic across spatial scales (Figs 2, 3, 4, 5, S2). Specifically, we show that Annonaceae frugivory‐related FRic was explained by the FRic of mammalian frugivores, supporting H1 (Fig. 4a). We also detected indirect drivers: SRic of mammalian frugivores, area size, and annual precipitation affected Annonaceae SRic, which in turn affected Annonaceae FRic. Although frugivorous mammal FRic accounted for a small portion of the variance explained in Annonaceae FRic, mammal SRic had a substantial role in explaining the variance in Annonaceae SRic, which, in turn, accounted for most of the variance explained in Annonaceae FRic. While we did not detect any frugivory‐exclusive direct driver of Annonaceae FRic within biogeographical realms, SEMs revealed indirect drivers via Annonaceae SRic, thus supporting H2. Specifically, mammal SRic affected Annonaceae SRic in the Afrotropics and Neotropics, and bird SRic affected Annonaceae SRic in the Asia‐Pacific region (Figs 5, S7–S9). Finally, we found that fruit sizes and growth forms of Annonaceae matched body mass variation of co‐occurring frugivorous mammals across broad‐scale assemblages globally, supporting H3.

### Mammals as an important driver of global Annonaceae species richness and frugivory‐related functional richness

Our results show that the diversity of frugivorous mammals has been a strong driver of Annonaceae SRic and frugivory‐related FRic across global assemblages (Figs 4a, S5a), even after accounting for spatial autocorrelation, abiotic environmental variables, and confounding diversity associations between plants and vertebrates more generally (by null model simulations and comparative tests with non‐frugivores) (Figs 4b, S4, S5b, S6). This supports the ‘dispersal syndrome’ theory (Valenta & Nevo, 2020) and suggests that frugivory‐related processes are important for shaping the broad‐scale diversity of tropical plants. This could have resulted from evolutionary mechanisms – that is reciprocal adaptations and plant–frugivore co‐diversification dynamics as illustrated in palms (Onstein *et al*., 2017, 2020), primates (Gómez & Verdú, 2012; Fuzessy *et al*., 2022), lizards (Kahnt *et al*., 2023), and bats (Rojas *et al*., 2012). However, whether plant–frugivore co‐diversification has also influenced the evolutionary radiations in Annonaceae (Xue *et al*., 2020) needs to be tested in an explicit phylogenetic framework. Alternatively, ecological mechanisms, such as niche partitioning through resource–consumer dynamics (the complementary specialization of species on exclusive interaction partners), may have shaped the large‐scale interaction network of Annonaceae and frugivorous mammals across tropical regions (e.g. Albrecht *et al*., 2018; Durand‐Bessart *et al*., 2023). Indeed, resource–consumer interactions may be important for SRic across communities, as illustrated for frugivorous birds and fig plants in sub‐Saharan Africa (Kissling *et al*., 2007). Furthermore, with increasing niche partitioning – for example due to frugivory‐related trait matching among species – functional diversity may also increase (Vázquez *et al*., 2009; Albrecht *et al*., 2018). By contrast, a reduction in functional diversity in one trophic level may cause a reduction in niche partitioning and hence functional diversity in the other trophic level (niche contraction and convergence; Albrecht *et al*., 2018). This provides a potential explanation for the matching in FRic between Annonaceae and frugivorous mammals across broad‐scale assemblages.

We also detected ‘matching’ of individual frugivory‐related mammal and Annonaceae traits that may explain the global matching of frugivorous mammal and Annonaceae functional diversity. For example, we detected an association between Annonaceae fruit size and mammal body mass. This evidences how body mass and gape size may constrain the size of ingested fruits (Fleming & Kress, 2013; Galetti *et al*., 2013). This fruit size–body size relationship has important consequences for long‐distance seed dispersal (Jordano *et al*., 2007; Onstein *et al*., 2019), plant speciation (Onstein *et al*., 2017), and the turnover of plant species across assemblages (Méndez *et al*., 2022), because large‐bodied animals – at least historically – have larger home ranges and move over longer distances than small‐bodied animals (Carbone *et al*., 2005). With the Quaternary extinctions of many large‐bodied or ‘megafaunal’ mammals (> 10 kg or > 45 kg; e.g. elephant relatives or giant sloths; Martin & Klein, 1984; Sandom *et al*., 2014), it is possible that many large‐fruited Annonaceae (i.e. fruits > 4 cm diameter; Guimarães *et al*., 2008; corresponding to *c*. 22% of the Annonaceae species in our dataset) will suffer from dispersal limitation and could become ‘anachronistic’ in ecosystems (Janzen & Martin, 1982). These extinctions may also impact overall fruit consumption, as megafauna, such as elephants (Table S5), despite consuming fruits as a relatively small part of their diet (< 50%, therefore, not reaching the threshold used in our framework to classify species as frugivores), contribute to a significant fraction of frugivory due to their high standing biomass (Pedersen *et al*., 2023). We also found significant trait matching between Annonaceae growth form and mammal body mass (Table S8). This suggests that vertical stratification has probably been important for niche partitioning in Annonaceae and frugivorous mammals (e.g. body size linked to habitat preference, thereby partitioning environments in vertical dimensions; e.g. Wong, 1986; Shanahan & Compton, 2001).

Our data did not capture the full set of traits relevant for seed dispersal in Annonaceae, such as seed size, fruit crop mass, fruit color, and chemical/scent traits. However, such traits may further explain the diversity of Annonaceae (and other tropical plants) across assemblages (Bender *et al*., 2018; Nevo *et al*., 2018; Sinnott‐Armstrong *et al*., 2018; Petrocelli *et al*., 2024). Annonaceae species may also be dispersed through synzoochory (seed dispersal via seed‐caching animals, which also involves seed predation) and stomatochory (in which seeds are carried externally without ingestion) (McConkey *et al*., 2024). Furthermore, it is important to interpret our findings with caution due to methodological limitations such as incomplete species sampling (i.e. analyses based on 52% of the total number of Annonaceae species that contributed to *c*. 63% of the total spatial coverage for the family) and the potential influence of unmeasured variables. Finally, historic and prehistoric extinctions of frugivores and their functional diversity (Duncan *et al*., 2013; Faurby & Svenning, 2015; Sayol *et al*., 2021) may have limited our ability to detect frugivory‐related diversity matching (e.g. at continental scales; Fig. 5).

### Biogeographical differences in frugivory‐related functional diversity relationships

While we found no direct frugivory‐exclusive effects on the FRic of Annonaceae across biogeographical realms, our SEMs revealed such effects on Annonaceae SRic, which, in turn, strongly influenced Annonaceae FRic (Fig. 5). Interestingly, while mammalian frugivores consistently explained the distribution of Annonaceae diversity globally and also within biogeographical realms (i.e. Afrotropics and Neotropics), SEMs revealed strong effects of frugivorous birds on Annonaceae SRic (and indirectly on Annonaceae FRic) in the Asia‐Pacific region. This finding aligns with our expectation, given the high diversity of frugivorous birds and predominance and diversity of strong‐flying frugivores, such as fruit pigeons (Columbiformes) and hornbills (Bucerotiformes), in this region (Shanahan *et al*., 2001; Holbrook *et al*., 2002). Indeed, empirical evidence validates the importance of these bird clades in dispersing Annonaceae species in the Asia‐Pacific region (Table S1). These findings suggest that differences in major mammalian frugivore guilds between realms (e.g. predominance of primate frugivores in Africa, high diversity of fruit bats in Asia‐Pacific) may explain differences in Annonaceae functional diversity between continents, for example high diversity of moniliform fruits in the Afrotropics (where *c*. 61% of species with this trait in our dataset occur) and Asia‐Pacific region (*c*. 38%), or of large (pseudo‐)syncarpous fruits in the Neotropics (*c*. 79%) and Afrotropics (*c*. 22%). By contrast, differences in avian frugivore guilds may be more important for regional differences in Annonaceae functional diversity, that is within the Asia‐Pacific realm (Fleming & Kress, 2011). These biogeographical differences may have resulted from historical contingencies shaping evolutionary histories of plants and animals through processes such as trait evolution, niche conservatism, and past intercontinental and climatic shifts (Kissling *et al*., 2009, 2012; Wölke *et al*., 2023). Such past dynamics may explain contemporary differences in frugivory‐related traits between regions (Onstein *et al*., 2017; Wölke *et al*., 2023), including differences in Annonaceae fruit traits (Onstein *et al*., 2019). Intriguingly, our results revealed a negative effect of frugivorous birds on the FRic of Annonaceae in the Neotropics, which is particularly surprising given the high diversity and ecological dominance of frugivorous birds in this region (Kissling *et al*., 2009; Fleming & Kress, 2011; this study). However, the Neotropics are also home to other keystone fruit resources for frugivores (Messeder *et al*., 2020), and high competition for seed dispersers could obscure the direct relationship between frugivory‐related birds and Annonaceae diversity specifically. Alternatively, convergence in the phenotypes of Neotropical frugivorous birds may lead to a negative association with Annonaceae functional diversity, and our results may not fully capture local‐scale interactions between Annonaceae and frugivorous birds in the Neotropics due to the spatial resolution of our data, methodological limitations, or historical and prehistoric extinctions of key frugivores that have disrupted Annonaceae–bird interactions.

### Abiotic environment impacts Annonaceae species richness and frugivory‐related traits

Plant diversity is also explained by factors other than frugivory, such as precipitation, temperature, and the supply of usable energy in the environment (Hawkins *et al*., 2003; Field *et al*., 2005). Indeed, we found strong common effects of abiotic variables on the diversity of Annonaceae and frugivorous birds and mammals (Fig. 4a), with the highest Annonaceae diversity in areas with high annual precipitation (Figs 4a, S7a, S8a) and with low variation in elevation (Figs S7a, S8a, S9a), that is in lowland tropical forests (Richardson *et al*., 2004; Couvreur *et al*., 2011). Furthermore, high annual temperatures led indirectly to high Annonaceae SRic via positive effects on frugivore SRic (Fig. 4a). Annonaceae species are an important ecological component of lowland tropical forest ecosystems (Richardson *et al*., 2004; Couvreur *et al*., 2011), and abundance and SRic are generally highest in high temperature and rainfall areas (see Punyasena *et al*., 2008), providing the conditions (water availability and temperature stability) for coexistence in diverse assemblages (Leigh *et al*., 2004). Niche conservatism linked to rainfall availability and warmer temperatures may therefore be important for Annonaceae diversification, with the majority of speciation events taking place within a specific niche (e.g. as for mimosoid legumes – Ringelberg *et al*., 2023). Precipitation and temperature have also been shown to be important drivers for fruit trait variation at regional and global scales (Zhao *et al*., 2018; McFadden *et al*., 2022), consistent with Annonaceae FRic patterns.

### Conclusion

Mutualistic interactions can either promote or limit SRic, depending on the specificity of the partnership, the level of partner dependence, and the mutualistic function (Chomicki *et al*., 2019). Our results are consistent with mutualistic seed dispersal interactions promoting species and frugivory‐related functional diversity – as Annonaceae SRic and FRic were positively associated with species and FRic of co‐occurring frugivorous mammals at global, Afrotropical, and Neotropical scales (Figs 4a, 5a,b) and with birds at the Asia‐Pacific scale (Fig. 5c). Our work builds on studies of particular animal groups or regions (e.g. Gómez & Verdú, 2012; Rojas *et al*., 2012; Albrecht *et al*., 2018) by providing a global view of how frugivores have shaped pantropical variation in plant diversity. We not only emphasize the importance of mutualisms for broad‐scale biodiversity patterns but also show that such biotic drivers of diversity are often in close concordance with the abiotic environment (Luo *et al*., 2023).

## Competing interests

None declared.

## Author contributions

AC and REO conceived the research, with input from IMAB, TLPC, SF, OH, IH, IK, CR‐V, HS and JAT. AC, CR‐V, REO and TLPC collected the data. AC ran the analyses with input from IMAB, TLPC, SF, OH, IH, IK, CR‐V, HS, JAT and REO. AC and REO wrote the manuscript with contributions from IMAB, TLPC, SF, OH, IH, IK, CR‐V, HS and JAT.

## Disclaimer

The New Phytologist Foundation remains neutral with regard to jurisdictional claims in maps and in any institutional affiliations.

## Supporting information


**Fig. S1** Frequency and distribution of missing data in Annonaceae trait matrix.
**Fig. S2** Distribution of Annonaceae and frugivorous bird and mammal species richness and functional richness across botanical countries.
**Fig. S3** Overview of spatial autocorrelation in the data.
**Fig. S4** Overview of null models (simulations).
**Fig. S5** Structural equation models at the global scale using a finer spatial resolution.
**Fig. S6** Overview of null models (simulations) using a more refined spatial resolution.
**Fig. S7** Structural equation models at the continental scale: Afrotropical region.
**Fig. S8** Structural equation models at the continental scale: Neotropical region.
**Fig. S9** Structural equation models at the continental scale: Asia‐Pacific region.
**Methods S1** Additional details on Annonaceae data, outliers, missing data and subsets, and on global models using a finer spatial resolution.
**Table S1** Pairwise interactions between native Annonaceae and frugivore species from plant–frugivore meta‐network.
**Table S2** Hypothesized trait matchings between frugivory‐related plant and animal traits.
**Table S3** Spatial information for Annonaceae species.
**Table S4** Number of bird and mammal species per family used in the analyses.
**Table S5** Number of bird and mammal species per family not included in our analyses.
**Table S6** List of botanical countries (assemblages), their respective continents, and the biogeographical realms used in the analyses.
**Table S7** Results for spatial autoregressive model.
**Table S8** Frugivory‐related trait matching between Annonaceae and mammalian frugivores at a global scale.
Please note: Wiley is not responsible for the content or functionality of any Supporting Information supplied by the authors. Any queries (other than missing material) should be directed to the *New Phytologist* Central Office.

## Data Availability

Data and code supporting the results are available at https://github.com/andressacabral/Annonaceae-frugivory-functional-diversity. The data are also archived on Figshare at doi: 10.6084/m9.figshare.28611935.v2.
