# Supplementary material for: Seed‐dispersing vertebrates and the abiotic environment shape functional diversity of the pantropical Annonaceae
Source: New Phytol. 2025 Apr 9;246(5):2263–79. doi: 10.1111/nph.70113 (PMC12059537; doi:10.1111/nph.70113)
Supplement: Supplementary file 1 — Fig. S1 Frequency and distribution of missing data in Annonaceae trait matrix. Fig. S2 Distribution of Annonaceae and frugivorous bird and mammal species richness and functional richness across botanical countries. Fig. S3 Overview of spatial autocorrelation in the data. Fig. S4 Overview of null models (simulations). Fig. S5 Structural equation models at the global scale using a finer spatial resolution. Fig. S6 Overview of null models (simulations) using a more refined spatial resolution. Fig. S7 Structural equation models at the continental scale: Afrotropical region. Fig. S8 Structural equation models at the continental scale: Neotropical region. Fig. S9 Structural equation models at the continental scale: Asia‐Pacific region. Methods S1 Additional details on Annonaceae data, outliers, missing data and subsets, and on global models using a finer spatial resolution. Table S1 Pairwise interactions between native Annonaceae and frugivore species from plant–frugivore meta‐network. Table S2 Hypothesized trait matchings between frugivory‐related plant and animal traits. Table S3 Spatial information for Annonaceae species. Table S4 Number of bird and mammal species per family used in the analyses. Table S5 Number of bird and mammal species per family not included in our analyses. Table S6 List of botanical countries (assemblages), their respective continents, and the biogeographical realms used in the analyses. Table S7 Results for spatial autoregressive model. Table S8 Frugivory‐related trait matching between Annonaceae and mammalian frugivores at a global scale. Please note: Wiley is not responsible for the content or functionality of any Supporting Information supplied by the authors. Any queries (other than missing material) should be directed to the New Phytologist Central Office. [file NPH-246-2263-s001.pdf]

Supplementary Information

Seed-dispersing vertebrates and the abiotic environment shape functional diversity of the pantropical Annonaceae

Andressa Cabral[1,2*], Irene M.A. Bender[3], Thomas L.P. Couvreur[4], Søren Faurby[5,6], Oskar Hagen[1,2], Isabell Hensen[1,7], Ingolf Kühn[1,7,8], Carlos Rodrigues-Vaz[4,9], Hervé Sauquet[10,11], Joseph A. Tobias[12], Renske E. Onstein[1,2,13*]

[1]German Centre for Integrative Biodiversity Research (iDiv) Halle – Jena – Leipzig, Puschstrasse 4, Leipzig 04103 , Germany

[2]Institute of Biology, Leipzig University, Leipzig 04103, Germany

[3]Instituto de Ecología Regional CONICET-UNT, Residencia Universitaria Horco Molle, ed. Las Cúpulas, Yerba Buena 4107, Argentina

[4]DIADE, University of Montpellier, CIRAD, IRD, 911 avenue d'Agropolis, Montpellier 34394, France

[5]Department of Biological and Environmental Sciences, University of Gothenburg, Box 461, SE 40530 Göteborg, Sweden

[6]Gothenburg Global Biodiversity Centre, Box 461, SE 40530 Göteborg, Sweden

[7]Institute of Biology/Geobotany and Botanical Garden, Martin Luther University Halle-Wittenberg, Große Steinstraße 79/80, Halle (Saale) 06108, Germany

[8]Helmholtz Centre for Environmental Research – UFZ, Department Community Ecology, Theodor-Lieser-Str. 4, Halle (Saale) 06120, Germany

[9]Institut de Systématique, Evolution, Biodiversité (ISYEB), Muséum National d'Histoire Naturelle-CNRS-SU-EPHE-UA, Paris 75005, France

[10]National Herbarium of New South Wales (NSW), Botanic Gardens of Sydney, Mount Annan NSW 2567, Australia

[11]Evolution and Ecology Research Centre, School of Biological, Earth and Environmental Sciences, University of New South Wales, Sydney NSW 2052, Australia

[12]Imperial College London, Faculty of Natural Sciences, Department of Life Sciences (Silwood Park), SL5 7PY, UK

[13]Naturalis Biodiversity Center, Darwinweg 2, Leiden 2333 CR, The Netherlands

***Corresponding authors: acabral@outlook.com.br; onsteinre@gmail.com**

Article acceptance date: 13 March 2025

Methods S1

Annonaceae data, outliers, missing data and subsets

The Annonaceae taxonomy used in TDWG did not fully match the taxonomy of the trait data. The latter followed taxonomy by Couvreur *et al*., in prep.; see our digital repository for species names. Sample quantiles were used to identify outliers in the continuous trait data (1% threshold) for each fruit type (syncarpous, i.e., developed from congenitally fused carpels; apocarpous, i.e., from free carpels; pseudosyncarpous, i.e. from monocarps fused after fecundation, van Setten *et al*., 1992). Outliers were subsequently checked with information from the original source, and corrected when needed. Missing values (NAs) can generate a biased distance matrix in subsequent analyses (i.e., functional distance matrix) by weighting the NAs as zeros (see Brown *et al*., 2012). We therefore explored two parallel approaches to deal with the occurrence of NAs in our trait matrix. First, all species with at least one NA were removed from the dataset, resulting in a matrix with 1,274 spp. (i.e., ca. 52% of the total number of Annonaceae species). Second, a non-parametric missing data imputation approach for mixed-type data using Random Forest ('missForest' package, Stekhoven & Bühlmann, 2012) was applied. However, given the substantial number of NAs (left panel in Fig. S1), visually non-homogeneous distribution of missing data throughout the matrix (right panel in Fig. S1), and low accuracy of predictions for continuous traits (Normalized Root Mean Squared Error = 0.72), we excluded the resulting imputed matrix from subsequent analyses.

Assessing global drivers of Annonaceae species richness and functional richness at a finer spatial resolution

To evaluate whether the relationship between the diversity (i.e., species richness and functional richness, hereafter SRic and FRic, respectively) of Annonaceae and frugivores is due to the area size of assemblages (i.e., botanical countries), we repeated the global piecewise structural equation models (SEMs) using a more refined spatial scale (i.e., cell size of 1 × 1 degree, ca. 110 × 110 km, based on the Behrmann cylindrical equal-area projection with standard parallels at 30°).

Annonaceae occurrence data were obtained from Erkens *et al*. (2022), where a taxonomically verified spatial dataset is provided. This dataset provides species coordinates for 1,225 species and 88 genera of Annonaceae globally (50% and 79.28% of the total number of species and genera, respectively; total numbers following the World Checklist of Vascular Plants, Govaerts *et al*., 2021). Species occurrence per assemblage (i.e., grid cells) was calculated using the R-package 'terra' specifying the relations between polygons and coordinates as "coveredby" and "overlaps" (Hijmans *et al*., 2022). Spatial information of resident and native current birds and

mammals was obtained from Coelho *et al.* (2023). This dataset provided occurrence of species per grid cells of 110-km resolution with a Behrmann equal area projection, and used occurrence ranges from BirdLife and IUCN, matching with the sources used in our primary analyses with botanical countries. Additionally, this dataset provides averaged current climatic variables (i.e., on temperature and water availability) within each spatial grid cell.

Following the same procedure as described in the main manuscript, we performed structural equation models (SEMs) to investigate direct and indirect effects explaining the global variation of Annonaceae SRic and FRic. These analyses were performed including frugivores (i.e., species with at least 50% fruit in diet), annual precipitation and temperature and precipitation seasonality as predictors. To assess whether an observed relationship between the SRic and FRic of Annonaceae and frugivores could have resulted from effects other than their frugivory-related interactions, we conducted a second set of SEMs using only SRic and FRic of non-frugivorous birds and mammals (i.e., species with 0% in the diet) as biotic variables.

Supplementary Figures

Figure S1: Frequency (left panel) and distribution (right panel) of missing data in the initial trait matrix with 1,895 Annonaceae species. Missing data is represented in red and trait data in blue.

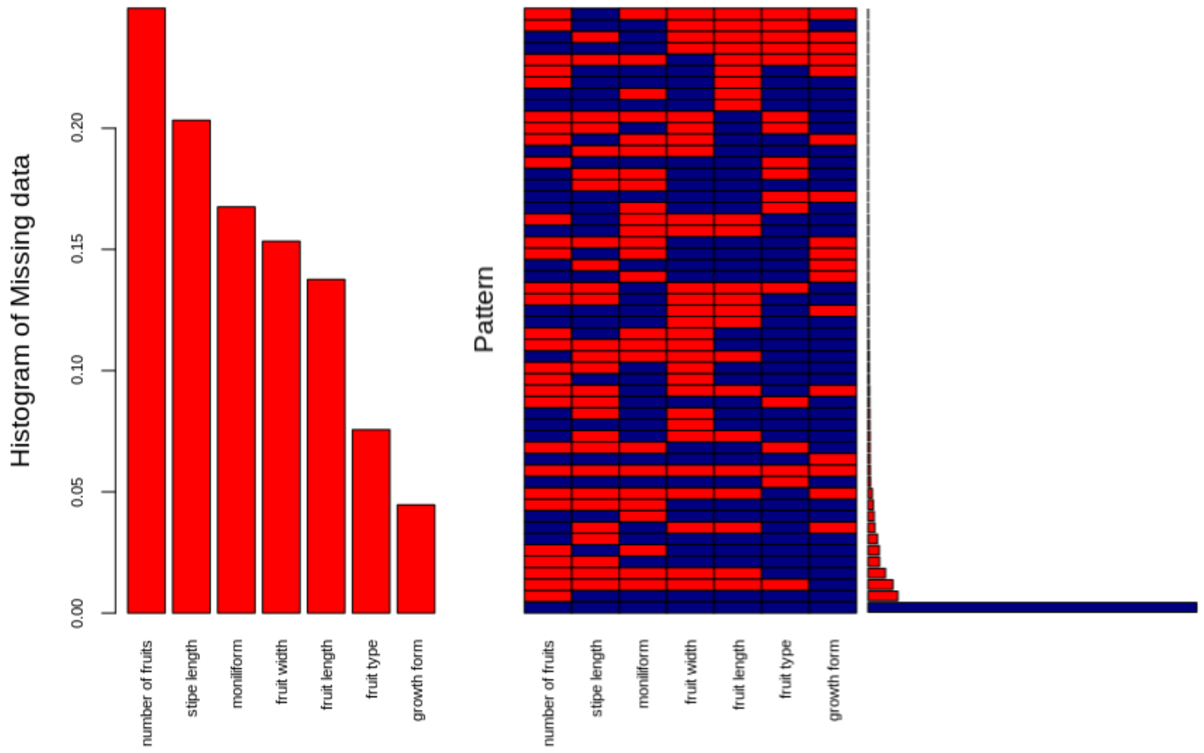

Figure S2: Global distribution of Annonaceae and frugivorous bird and mammal species richness (SRic) and frugivory-related functional richness (FRic) captured by the main global SEMs. (a) Annonaceae, (b) frugivorous birds and (c) frugivorous mammals. Bird and mammal values were based on a subset of frugivorous species with at least 50% of fruits in diet. Outer circles are coloured by functional richness and inner circles by species richness. Circles correspond to the same assemblages as the ones used in the global SEMs, specifically co-occurring Annonaceae species alongside frugivorous birds and mammals. The minimum species count per assemblage was determined by the number of Principal Coordinate Analysis (PCoA) axes used to calculate functional richness, ensuring species counts exceeded the corresponding PCoA axes. Species richness ranges from 4 – 212 spp. (a), 6 – 253 spp. (b), or 3 – 77 spp. (c). All raw values were rescaled to 0–1 for visualisation purposes, and plotted at the centroid position of the botanical countries.

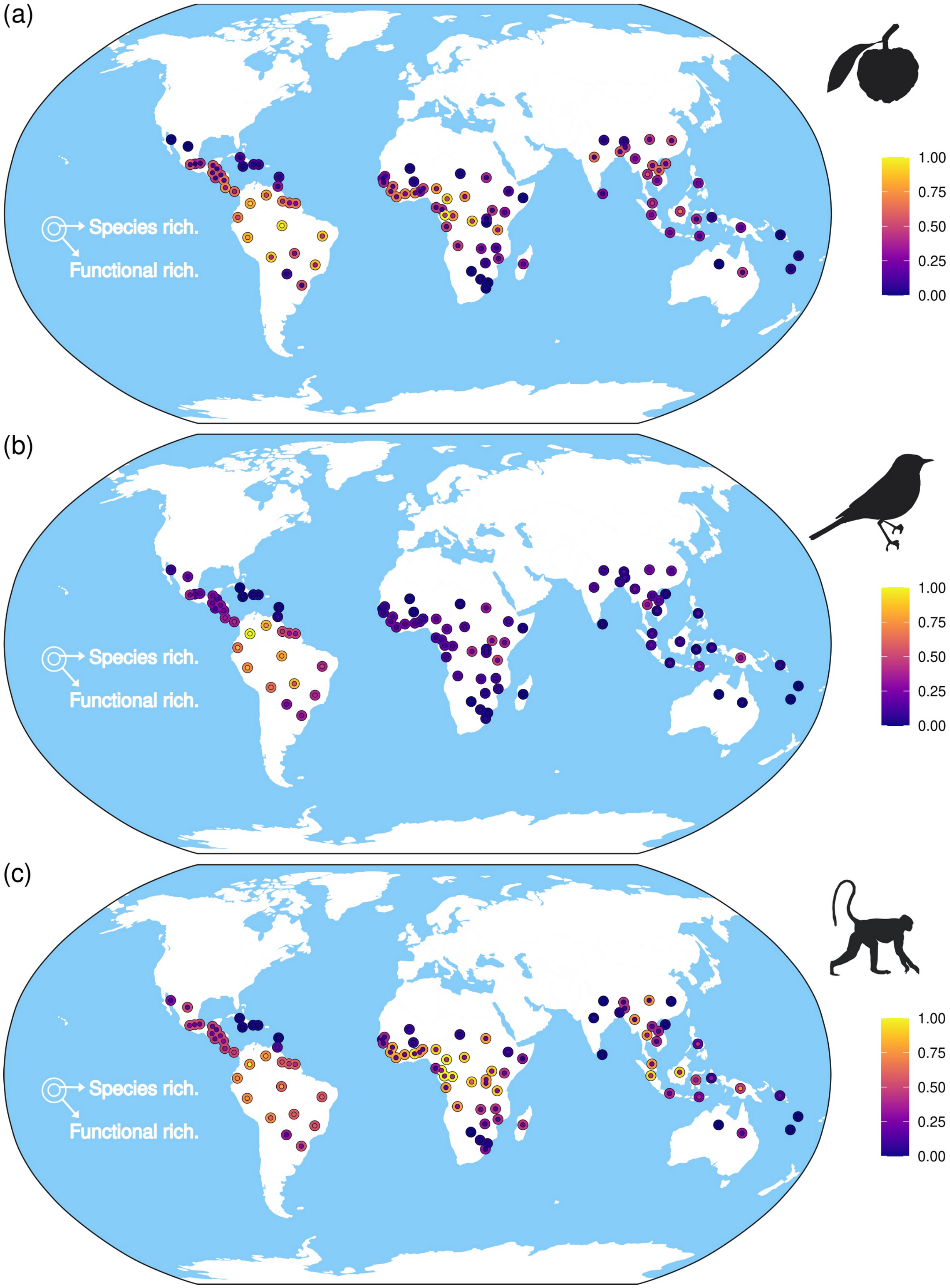
(a)
Species rich.
Functional rich.
1.00
0.75
0.50
0.25
0.00
(b)
Species rich.
Functional rich.
1.00
0.75
0.50
0.25
0.00
(c)
Species rich.
Functional rich.
1.00
0.75
0.50
0.25
0.00

Figure S3: Spatial autocorrelation in the residuals from the ordinary least square (linear) model (OLS) (white dots) and spatial error model (black squares). The y-axis (Moran's I) quantifies spatial autocorrelation: values close to zero suggest a random spatial distribution, negative values indicate dispersion, and positive values indicate clustering of observations. The x-axis (distance class) represents the division of spatial distances between observation points into discrete intervals. Spatial autocorrelation was analysed separately for equations in which (a) Annonaceae functional richness and (b) Annonaceae species richness were the response variables, both derived from the structural equation model assessing direct and indirect effects on Annonaceae FRic across botanical countries.

(a) Annonaceae functional richness as response variable

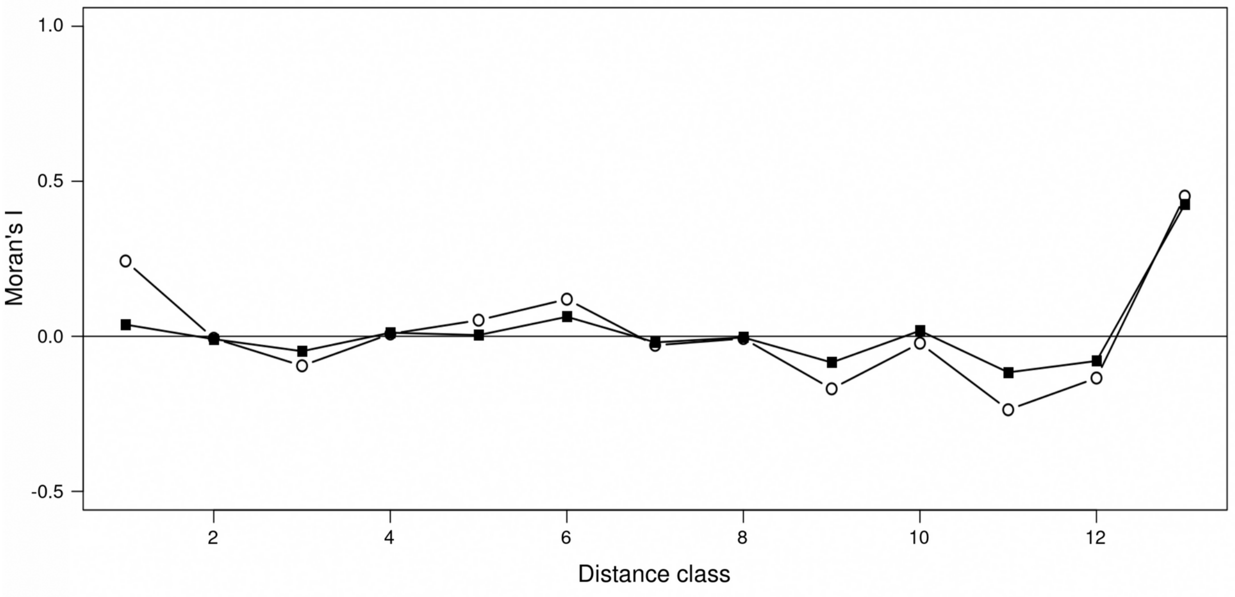

(b) Annonaceae species richness as response variable

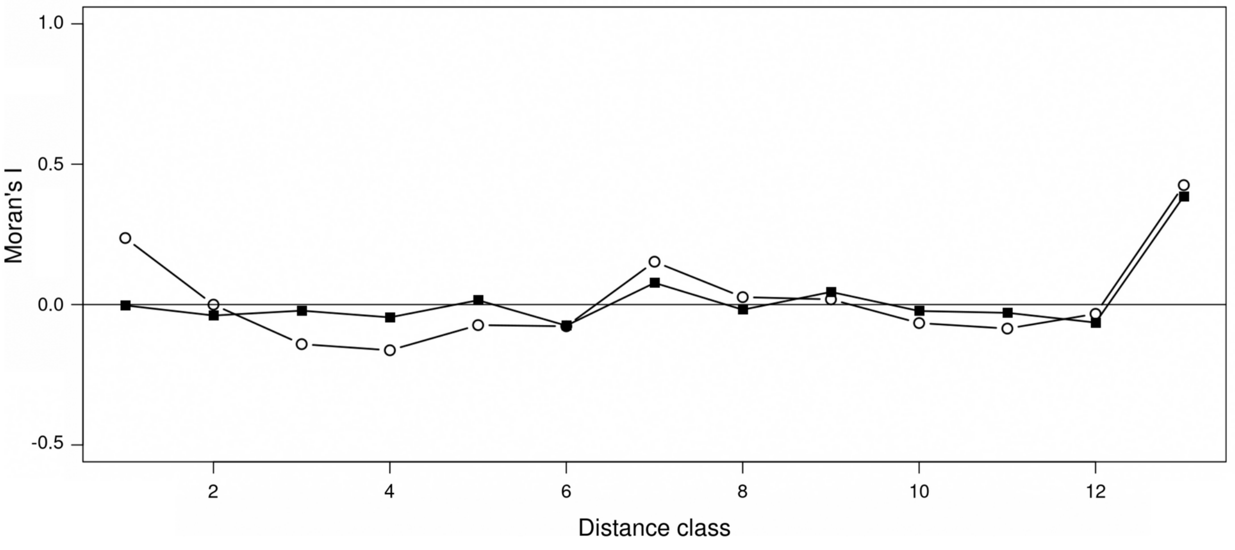

Figure S4: Distribution of the observed and simulated frugivore effect sizes and significance on Annonaceae functional richness (a,c) and on Annonaceae species richness (b,d). Mammal SRic and FRic were based on a subset of frugivorous species with at least 50% of fruits in diet. In this null model approach, we accessed the distribution and significance of frugivore effects on a simulated Annonaceae SRic and FRic (i.e., 1000 random shuffling across assemblages). Observed effect sizes and significance from the empirical models (see Fig. **3a**) are indicated in red. Observed estimates deviated from a random expectation globally, with observed frugivore effect sizes strongly deviating from mean simulated estimates and falling outside the distribution of approx. 95% of simulated effect sizes on Annonaceae SRic and FRic. Over 93.5% of the simulated effects on Annonaceae SRic and FRic were not significant at $p < 0.05$. Abbreviations: “FRic” = functional richness, “SRic” = species richness.

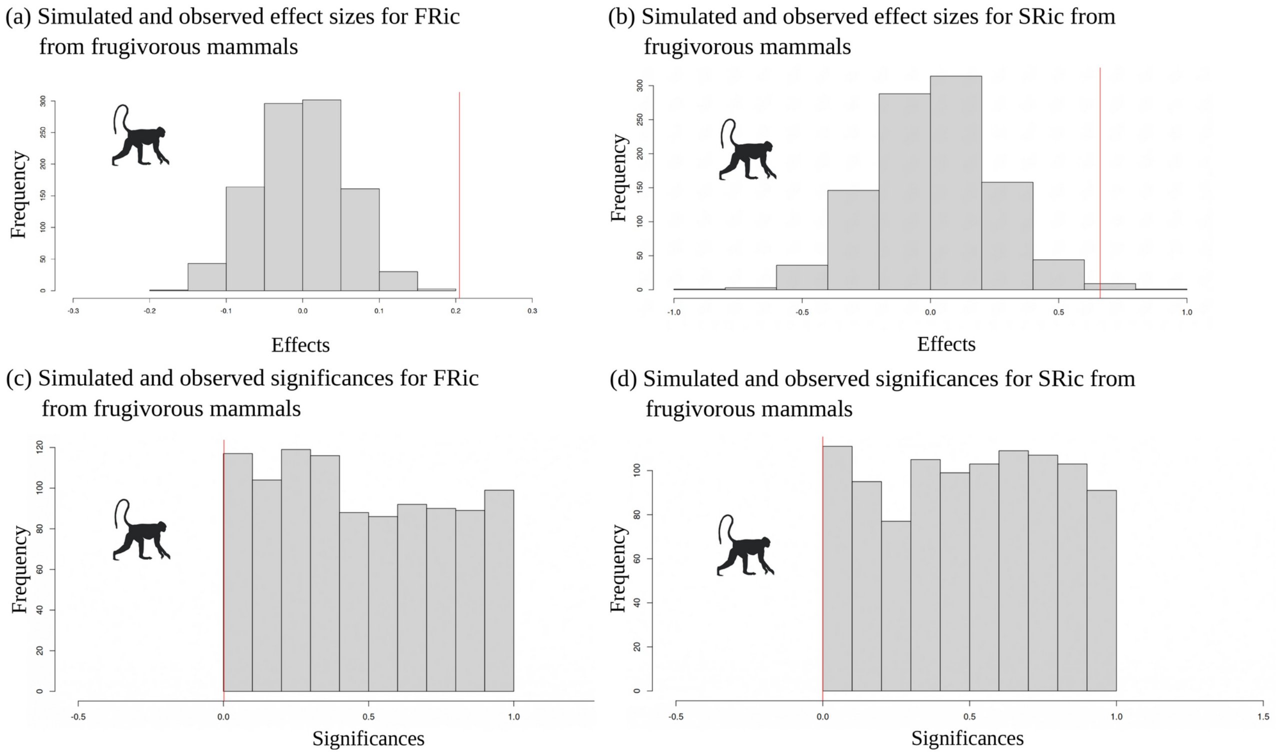

Figure S5: Global drivers of Annonaceae species richness and frugivory-related functional richness using grid cells of 1 × 1 degree. (a) Structural equation model (SEM) representing the standardized effects of predictor variables on Annonaceae, frugivorous bird and frugivorous mammal SRic and frugivory-related FRic at the global scale. Bird and mammal SRic and FRic were based on a subset of frugivorous species with at least 50% of fruits in diet. (b) Comparative SEM with non-frugivores. In this case, bird and mammal SRic and FRic were based on a subset of species with no fruit in their diet. For both (a) and (b), only statistically significant effects (standardized coefficients with $p < 0.05$) are shown. Grid cells (1 × 1 degree, ca. 110 × 110 km, Behrmann equal-area projection) were used as assemblages. Standardized coefficients reflect the change in the response variable per unit change in the predictor, conditional on all other variables being held constant. Arrows indicate the direction of the effect, with arrow thickness proportional to effect strength. Red arrows represent negative effects. Positive biotic effects that were significant in the model with frugivores (a), but not significant in the model with non-frugivores (b), are highlighted with a star. These suggest that the association between frugivores and Annonaceae is due to frugivory-related interactions, rather than due to co-variation between Annonaceae and frugivore SRic and/or FRic because of other factors. R^2 of response variables refers to the explained variation by all the predictor variables. Abbreviations: “FRic” = functional richness, “SRic” = species richness. Prior to model selection, FRic of birds and mammals, as well as SRic of Annonaceae, birds, and mammals, were square root-transformed. A covariance parameter between bird and mammal SRic was included *a priori* in the base model. The model's modification indices were evaluated, and when necessary, *a posteriori* covariance parameters were incorporated to improve the overall model fit. These included: (a) bird FRic and mammal FRic, mammal FRic and bird SRic, mammal FRic and mammal SRic, bird FRic and bird SRic, bird FRic and mammal SRic, mammal FRic and Annonaceae SRic, bird FRic and Annonaceae SRic, and Annonaceae FRic and bird SRic; (b) mammal FRic and bird SRic, mammal FRic and mammal SRic, bird FRic and Annonaceae SRic, mammal FRic and Annonaceae SRic, Annonaceae FRic and mammal SRic, and bird FRic and mammal FRic. Optimal model fit: (a) scaled p-value of $\chi2$-test = 0.542, robust comparative fit index (CFI) = 1.000 and robust root mean square error of approximation (RMSEA) = 0.000; (b) scaled p-value of $\chi2$-test = 0.092, robust CFI = 0.998 and robust RMSEA = 0.021.

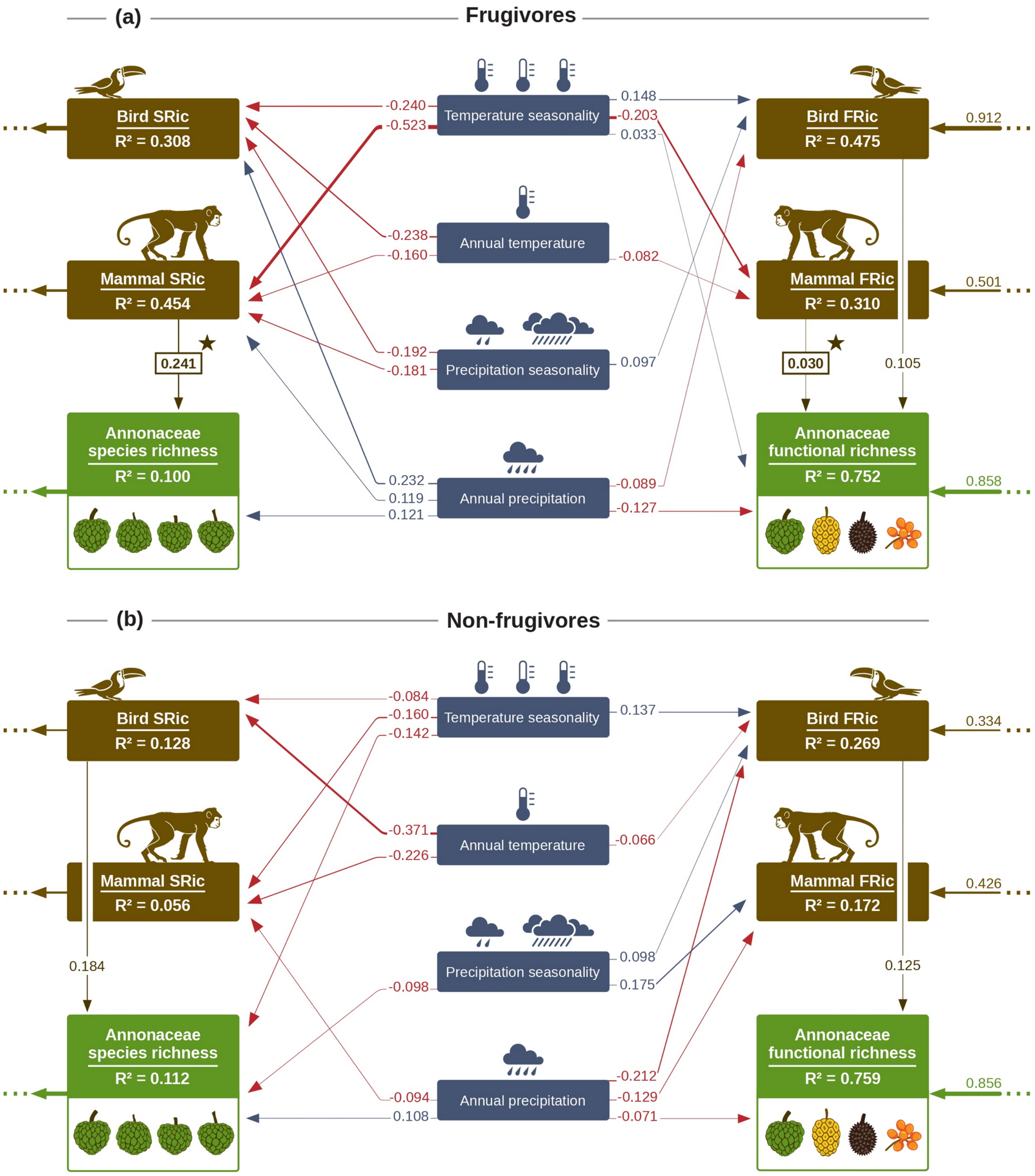
(a)
Frugivores
Bird SRic
R² = 0.308
Mammal SRic
R² = 0.454
0.241
Annonaceae species richness
R² = 0.100
Temperature seasonality
Annual temperature
Precipitation seasonality
Annual precipitation
-0.240
-0.523
-0.238
-0.160
-0.192
-0.181
0.232
0.119
0.121
0.148
-0.203
0.033
-0.082
0.097
-0.089
-0.127
Bird FRic
R² = 0.475
Mammal FRic
R² = 0.310
0.030
0.105
Annonaceae functional richness
R² = 0.752
0.912
0.501
0.858
(b)
Non-frugivores
Bird SRic
R² = 0.128
Mammal SRic
R² = 0.056
0.184
Annonaceae species richness
R² = 0.112
Temperature seasonality
Annual temperature
Precipitation seasonality
Annual precipitation
-0.084
-0.160
-0.142
-0.371
-0.226
-0.098
-0.094
0.108
0.137
-0.066
0.098
0.175
-0.212
-0.129
-0.071
Bird FRic
R² = 0.269
Mammal FRic
R² = 0.172
0.125
Annonaceae functional richness
R² = 0.759
0.334
0.426
0.856

Figure S6: Distribution of the observed and simulated frugivore effect sizes and significance on Annonaceae functional richness (a,c) and on Annonaceae species richness (b,d) for models using grid cells of 1 × 1 degree. Mammal SRic and FRic were based on a subset of frugivorous species with at least 50% of fruits in diet. In this null model approach, we accessed the distribution and significance of frugivore effects on a simulated Annonaceae SRic and FRic (i.e., 1000 random shuffling across assemblages). Observed effect sizes and significance from the empirical models (see Fig. **S6a**) are indicated in red. Observed estimates deviated from a random expectation globally, with observed frugivore effect sizes strongly deviating from mean simulated estimates and falling outside the distribution of approx. 95% of simulated effect sizes. Over 94% of the simulated effects on Annonaceae SRic and FRic were not significant at $p < 0.05$. Abbreviations: "FRic" = functional richness, "SRic" = species richness.

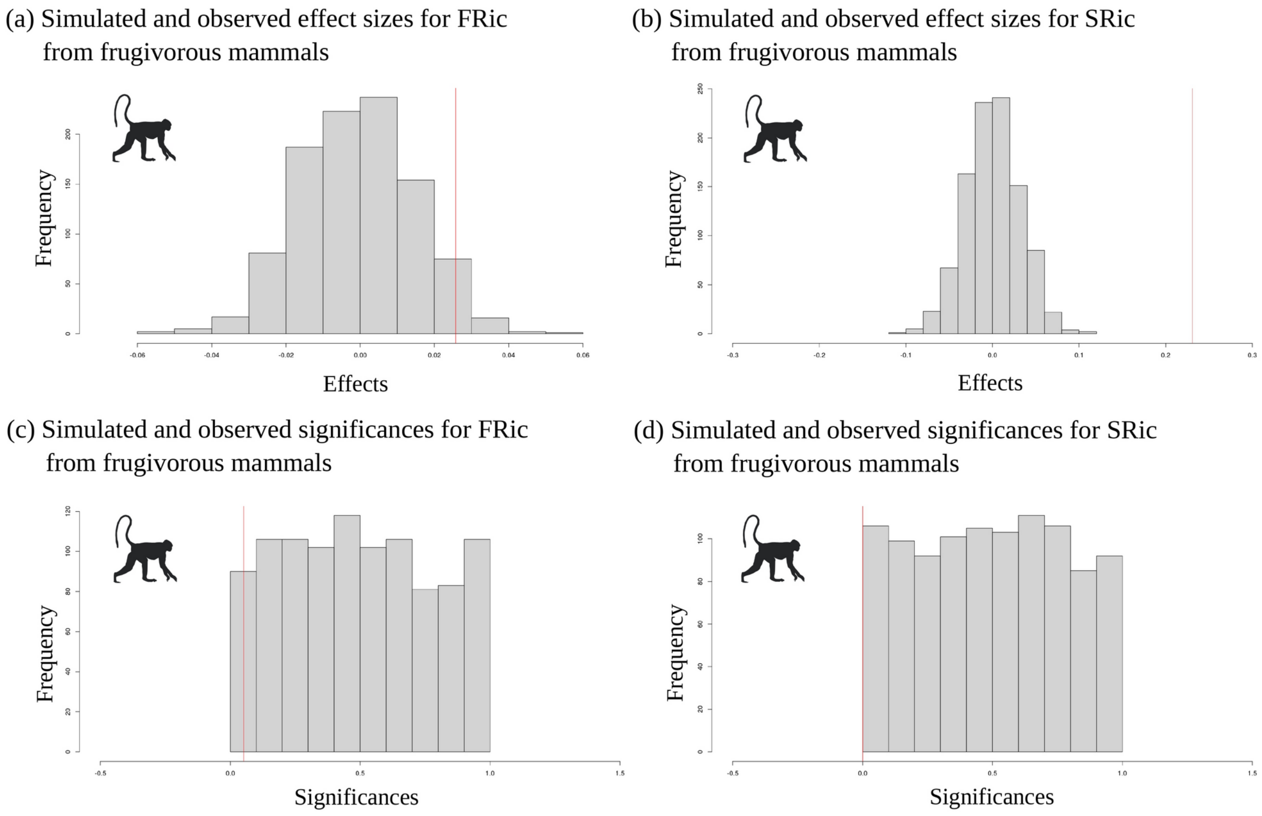

Figure S7: Drivers of Annonaceae species richness and frugivory-related functional richness in the Afrotropics. (a) Structural equation model (SEM) representing the standardized effects of predictor variables on Annonaceae, frugivorous bird and frugivorous mammal SRic and frugivory-related FRic in the Afrotropics. Bird and mammal SRic and FRic were based on a subset of frugivorous species with at least 50% of fruits in diet. (b) Comparative SEM with non-frugivores. In this case, bird and mammal SRic and FRic were based on a subset of species with no fruit in their diet. For both (a) and (b), only statistically significant effects (standardized coefficients with $p < 0.05$) are shown. Standardized coefficients reflect the change in the response variable per unit change in the predictor, conditional on all other variables being held constant. Arrows indicate the direction of the effect, with arrow thickness proportional to effect strength. Red arrows represent negative effects. Positive biotic effects that were significant in the model with frugivores (a), but not significant in the model with non-frugivores (b), are highlighted with a star. These suggest that the association between frugivores and Annonaceae is due to frugivory-related interactions, rather than due to co-variation between Annonaceae and frugivore SRic and/or FRic because of other factors. R^2 of response variables refers to the explained variation by all the predictor variables. Abbreviations: "FRic" = functional richness, "SRic" = species richness. Prior to model selection, FRic of birds and mammals, as well as SRic of Annonaceae, birds, and mammals, were square root-transformed. A covariance parameter between bird and mammal SRic was included *a priori* in the base model. The model's modification indices were evaluated, and when necessary, *a posteriori* covariance parameters were incorporated to improve the overall model fit. These included: (a) none; (b) none. Optimal model fit: (a) p-value of $\chi 2$-test = 0.304, comparative fit index (CFI) = 0.991; (b) p-value of $\chi 2$-test = 0.231, CFI = 0.984.

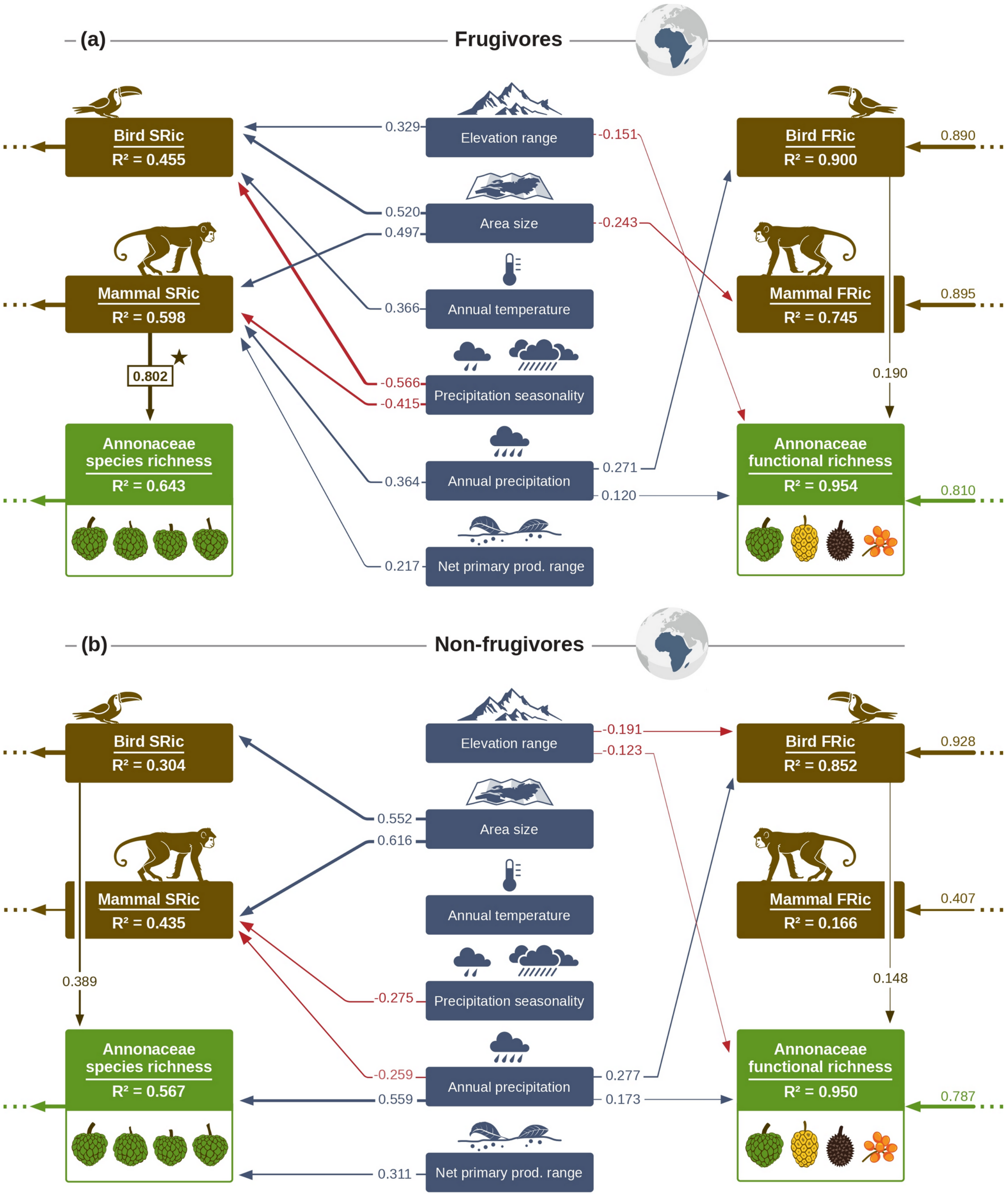
(a)
Frugivores
Bird SRic
R² = 0.455
Mammal SRic
R² = 0.598
0.802
Annonaceae species richness
R² = 0.643
Elevation range
Area size
Annual temperature
Precipitation seasonality
Annual precipitation
Net primary prod. range
0.329
0.520
0.497
0.366
-0.566
-0.415
0.364
0.217
-0.151
-0.243
0.271
0.120
Bird FRic
R² = 0.900
Mammal FRic
R² = 0.745
Annonaceae functional richness
R² = 0.954
0.890
0.895
0.190
0.810
(b)
Non-frugivores
Bird SRic
R² = 0.304
Mammal SRic
R² = 0.435
0.389
Annonaceae species richness
R² = 0.567
Elevation range
Area size
Annual temperature
Precipitation seasonality
Annual precipitation
Net primary prod. range
0.552
0.616
-0.275
-0.259
0.559
0.311
-0.191
-0.123
0.277
0.173
Bird FRic
R² = 0.852
Mammal FRic
R² = 0.166
Annonaceae functional richness
R² = 0.950
0.928
0.407
0.148
0.787

Figure S8: Drivers of Annonaceae species richness and frugivory-related functional richness in the Neotropics. (a) Structural equation model (SEM) representing the standardized effects of predictor variables on Annonaceae, frugivorous bird and frugivorous mammal SRic and frugivory-related FRic in the Neotropics. Bird and mammal SRic and FRic were based on a subset of frugivorous species with at least 50% of fruits in diet. (b) Comparative SEM with non-frugivores. In this case, bird and mammal SRic and FRic were based on a subset of species with no fruit in their diet. For both (a) and (b), only statistically significant effects (standardized coefficients with $p < 0.05$) are shown. Standardized coefficients reflect the change in the response variable per unit change in the predictor, conditional on all other variables being held constant. Arrows indicate the direction of the effect, with arrow thickness proportional to effect strength. Red arrows represent negative effects. Positive biotic effects that were significant in the model with frugivores (a), but not significant in the model with non-frugivores (b), are highlighted with a star. These suggest that the association between frugivores and Annonaceae is due to frugivory-related interactions, rather than due to co-variation between Annonaceae and frugivore SRic and/or FRic because of other factors. R^2 of response variables refers to the explained variation by all the predictor variables. Abbreviations: “FRic” = functional richness, “SRic” = species richness. Prior to model selection, FRic of birds and mammals, as well as SRic of Annonaceae, birds, and mammals, were square root-transformed. A covariance parameter between bird and mammal SRic was included *a priori* in the base model. The model's modification indices were evaluated, and when necessary, *a posteriori* covariance parameters were incorporated to improve the overall model fit. These included: (a) bird FRic and mammal FRic, bird FRic and Annonaceae SRic, and mammal FRic and Annonaceae SRic; (b) none. Optimal model fit: (a) p-value of $\chi 2$-test = 0.214, comparative fit index (CFI) = 0.990; (b) p-value of $\chi 2$-test = 0.133, CFI = 0.983.

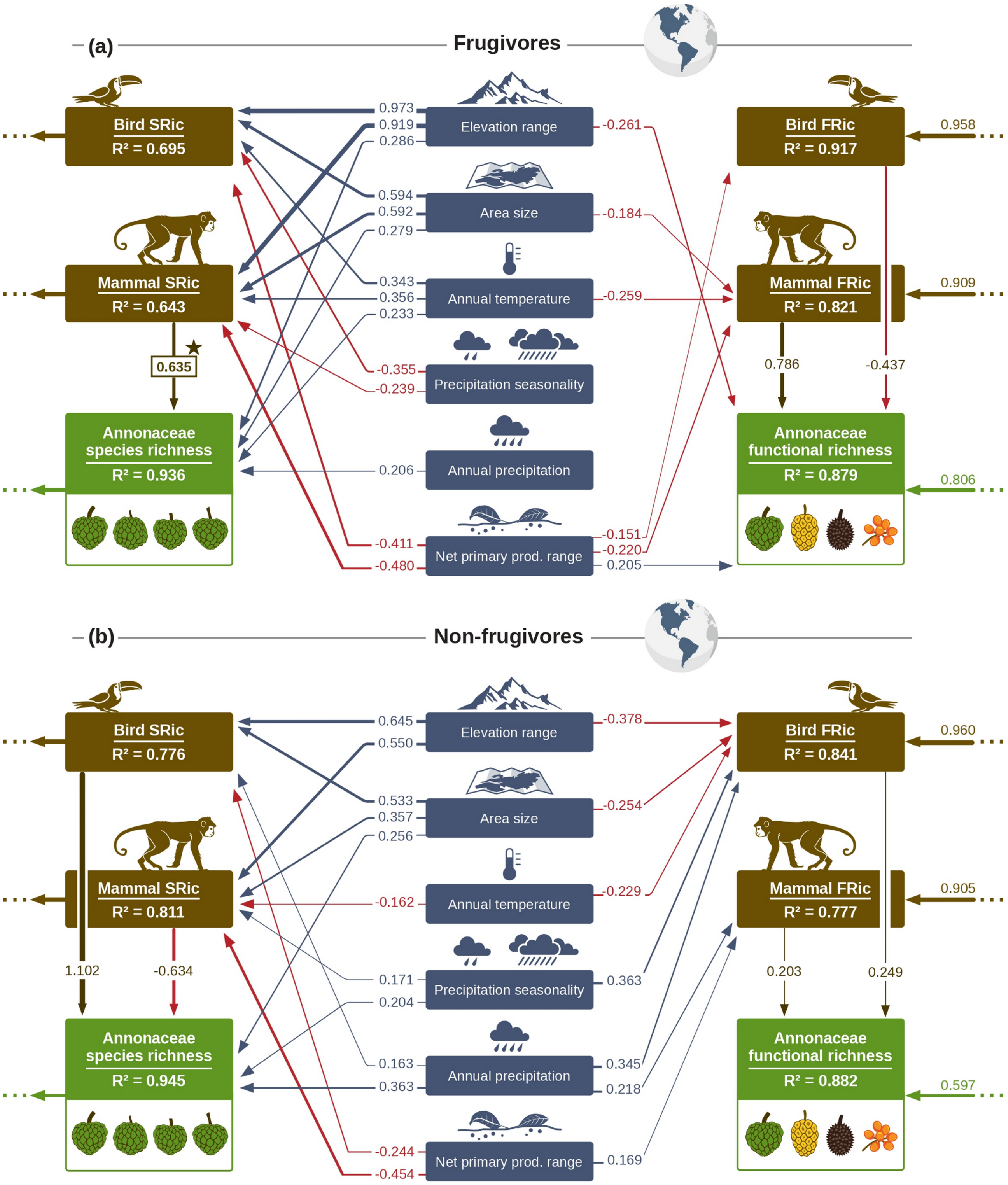
(a)
Frugivores
Bird SRic
R² = 0.695
Mammal SRic
R² = 0.643
0.635
Annonaceae species richness
R² = 0.936
Elevation range
Area size
Annual temperature
Precipitation seasonality
Annual precipitation
Net primary prod. range
0.973
0.919
0.286
0.594
0.592
0.279
0.343
0.356
0.233
-0.355
-0.239
0.206
-0.411
-0.480
-0.261
-0.184
-0.259
-0.151
-0.220
0.205
Bird FRic
R² = 0.917
0.958
Mammal FRic
R² = 0.821
0.909
0.786
-0.437
Annonaceae functional richness
R² = 0.879
0.806
(b)
Non-frugivores
Bird SRic
R² = 0.776
Mammal SRic
R² = 0.811
1.102
-0.634
Annonaceae species richness
R² = 0.945
Elevation range
Area size
Annual temperature
Precipitation seasonality
Annual precipitation
Net primary prod. range
0.645
0.550
0.533
0.357
0.256
-0.162
0.171
0.204
0.163
0.363
-0.244
-0.454
-0.378
-0.254
-0.229
0.363
0.345
0.218
0.169
Bird FRic
R² = 0.841
0.960
Mammal FRic
R² = 0.777
0.905
0.203
0.249
Annonaceae functional richness
R² = 0.882
0.597

Figure S9: Drivers of Annonaceae species richness and frugivory-related functional richness in the Asia-Pacific region. (a) Structural equation model (SEM) representing the standardized effects of predictor variables on Annonaceae, frugivorous bird and frugivorous mammal SRic and frugivory-related FRic in the Asia-Pacific region. Bird and mammal SRic and FRic were based on a subset of frugivorous species with at least 50% of fruits in diet. (b) Comparative SEM with non-frugivores. In this case, bird and mammal SRic and FRic were based on a subset of species with no fruit in their diet. For both (a) and (b), only statistically significant effects (standardized coefficients with $p < 0.05$) are shown. Standardized coefficients reflect the change in the response variable per unit change in the predictor, conditional on all other variables being held constant. Arrows indicate the direction of the effect, with arrow thickness proportional to effect strength. Red arrows represent negative effects. Positive biotic effects that were significant in the model with frugivores (a), but not significant in the model with non-frugivores (b), are highlighted with a star. These suggest that the association between frugivores and Annonaceae is due to frugivory-related interactions, rather than due to co-variation between Annonaceae and frugivore SRic and/or FRic because of other factors. R^2 of response variables refers to the explained variation by all the predictor variables. Abbreviations: “FRic” = functional richness, “SRic” = species richness. Prior to model selection, FRic of birds and mammals, as well as SRic of Annonaceae, birds, and mammals, were square root-transformed. A covariance parameter between bird and mammal SRic was included *a priori* in the base model. The model's modification indices were evaluated, and when necessary, *a posteriori* covariance parameters were incorporated to improve the overall model fit. These included: (a) mammal FRic and Annonaceae SRic; (b) none. Optimal model fit: (a) p-value of $\chi 2$-test = 0.259, comparative fit index (CFI) = 0.976; (b) p-value of $\chi 2$-test = 0.313, CFI = 0.991.

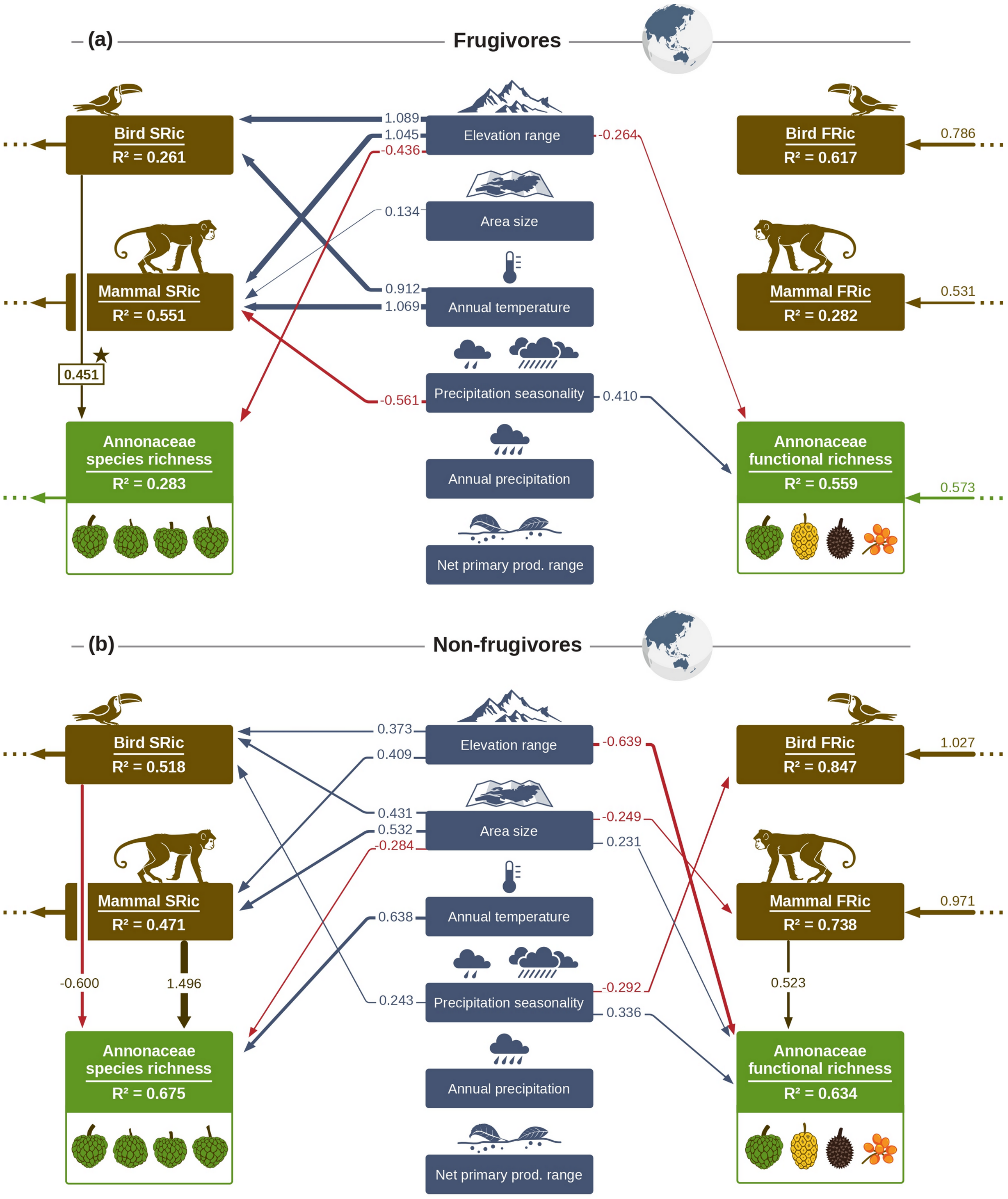

(a)
Frugivores
Bird SRic
R² = 0.261
Mammal SRic
R² = 0.551
Annonaceae species richness
R² = 0.283
Bird FRic
R² = 0.617
Mammal FRic
R² = 0.282
Annonaceae functional richness
R² = 0.559
Elevation range
Area size
Annual temperature
Precipitation seasonality
Annual precipitation
Net primary prod. range
1.089
1.045
-0.436
0.134
0.912
1.069
-0.561
-0.264
0.410
0.451
0.786
0.531
0.573
(b)
Non-frugivores
Bird SRic
R² = 0.518
Mammal SRic
R² = 0.471
Annonaceae species richness
R² = 0.675
Bird FRic
R² = 0.847
Mammal FRic
R² = 0.738
Annonaceae functional richness
R² = 0.634
Elevation range
Area size
Annual temperature
Precipitation seasonality
Annual precipitation
Net primary prod. range
0.373
0.409
0.431
0.532
-0.284
0.638
0.243
-0.639
-0.249
0.231
-0.292
0.336
-0.600
1.496
0.523
1.027
0.971

Supplementary Tables

Table S1: Pairwise interactions between native Annonaceae and frugivore species reported in a plant–frugivore meta-network (for further details, see Fricke & Svenning, 2020). Birds and mammals account for approximately 98% of the unique pairwise interactions with 87 Annonaceae species (3.55% of the total 2,448 Annonaceae species) in this meta-network, with birds representing 32% and mammals 66%, out of a total of 297 observations.

Study ID	Network ID	Locality	Region	Animal class	Animal order	Animal family	Animal accepted species	Plant family	Plant accepted species
M_SD_007	M_SD_007	Australia	Australian	Aves	Columbiformes	Columbidae	*Ptilinopus magnificus*	Annonaceae	*Cananga odorata*
M_SD_007	M_SD_007	Australia	Australian	Aves	Columbiformes	Columbidae	*Ptilinopus superbus*	Annonaceae	*Melodorum leichhardtii*
M_SD_010	M_SD_010 single partners added	Trinidad and Tobago	Amazonian	Aves	Passeriformes	Fringillidae	*Euphonia violacea*	Annonaceae	*Rollinia exsucca*
M_SD_012	M_SD_012	Brazil	South American	Aves	Passeriformes	Tyrannidae	*Pitangus sulphuratus*	Annonaceae	*Xylopia brasiliensis*
M_SD_012	M_SD_012	Brazil	South American	Aves	Passeriformes	Tyrannidae	*Tyrannus melancholicus*	Annonaceae	*Xylopia brasiliensis*
M_SD_019	M_SD_019	Costa Rica	Panamanian	Aves	Passeriformes	Turdidae	*Turdus plebejus*	Annonaceae	*Guatteria consanguinea*
M_SD_019	M_SD_019	Costa Rica	Panamanian	Aves	Passeriformes	Cotingidae	*Procnias tricarunculatus*	Annonaceae	*Guatteria consanguinea*
M_SD_019	M_SD_019	Costa Rica	Panamanian	Aves	Passeriformes	Tyrannidae	*Elaenia frantzii*	Annonaceae	*Guatteria consanguinea*
M_SD_019	M_SD_019	Costa Rica	Panamanian	Aves	Passeriformes	Turdidae	*Catharus ustulatus*	Annonaceae	*Guatteria consanguinea*
M_SD_01	M_SD_019	Costa Rica	Panamanian	Aves	Passeriformes	Turdidae	*Turdus grayi*	Annonaceae	*Guatteria*

9									*consanguinea*
M_SD_022	M_SD_022	Brazil	South American	Aves	Passeriformes	Pipridae	*Chiroxiphia caudata*	Annonaceae	*Guatteria australis*
M_SD_022	M_SD_022	Brazil	South American	Aves	Passeriformes	Turdidae	*Turdus albicollis*	Annonaceae	*Xylopia brasiliensis*
M_SD_022	M_SD_022	Brazil	South American	Aves	Passeriformes	Thraupidae	*Thraupis cyanoptera*	Annonaceae	*Xylopia brasiliensis*
M_SD_022	M_SD_022	Brazil	South American	Aves	Passeriformes	Fringillidae	*Euphonia pectoralis*	Annonaceae	*Xylopia brasiliensis*
M_SD_022	M_SD_022	Brazil	South American	Aves	Passeriformes	Turdidae	*Turdus flavipes*	Annonaceae	*Xylopia brasiliensis*
M_SD_022	M_SD_022	Brazil	South American	Aves	Piciformes	Ramphastidae	*Baillonius bailloni*	Annonaceae	*Xylopia brasiliensis*
M_SD_022	M_SD_022	Brazil	South American	Aves	Passeriformes	Icteridae	*Cacicus haemorrhous*	Annonaceae	*Xylopia brasiliensis*
M_SD_022	M_SD_022	Brazil	South American	Aves	Passeriformes	Tyrannidae	*Tityra cayana*	Annonaceae	*Xylopia brasiliensis*
M_SD_022	M_SD_022	Brazil	South American	Aves	Passeriformes	Cotingidae	*Lipaugus lanioides*	Annonaceae	*Xylopia brasiliensis*
M_SD_022	M_SD_022	Brazil	South American	Aves	Passeriformes	Tyrannidae	*Myiodynastes maculatus*	Annonaceae	*Xylopia brasiliensis*
M_SD_022	M_SD_022	Brazil	South American	Aves	Passeriformes	Thraupidae	*Habia rubica*	Annonaceae	*Xylopia brasiliensis*
M_SD_022	M_SD_022	Brazil	South American	Aves	Galliformes	Cracidae	*Aburria jacutinga*	Annonaceae	*Rollinia sericea*
M_SD_022	M_SD_022	Brazil	South American	Mammalia	Carnivora	Canidae	*Cerdocyon thous*	Annonaceae	*Rollinia emarginata*
M_SD_022	M_SD_022	Brazil	South American	Aves	Passeriformes	Fringillidae	*Orthogonys chloricterus*	Annonaceae	*Xylopia brasiliensis*
M_SD_022	M_SD_022	Brazil	South American	Aves	Passeriformes	Thraupidae	*Orchesticus abeillei*	Annonaceae	*Xylopia brasiliensis*
M_SD_022	M_SD_022	Brazil	South American	Aves	Psittaciformes	Psittacidae	*Pyrrhura frontalis*	Annonaceae	*Xylopia brasiliensis*

M_SD_022	M_SD_022	Brazil	South American	Mammalia	Primates	Atelidae	*Alouatta guariba*	Annonaceae	*Xylopia brasiliensis*
M_SD_022	M_SD_022	Brazil	South American	Aves	Psittaciformes	Psittacidae	*Brotogeris tirica*	Annonaceae	*Xylopia brasiliensis*
M_SD_022	M_SD_022	Brazil	South American	Aves	Passeriformes	Tyrannidae	*Pachyramphus validus*	Annonaceae	*Xylopia brasiliensis*
KITA mammals added	KITA mammals added	Thailand	Oriental	Mammalia	Primates	Hylobatidae	*Hylobates lar*	Annonaceae	*Platymitra macrocarpa*
KITA mammals added	KITA mammals added	Thailand	Oriental	Mammalia	Rodentia	Sciuridae	*Ratufa bicolor*	Annonaceae	*Platymitra macrocarpa*
SAAV	SAAV1	Bolivia	Amazonian	Aves	Piciformes	Ramphastidae	*Aulacorhynchus coeruleicinctis*	Annonaceae	*Guatteria glauca*
SAAV	SAAV2	Bolivia	Amazonian	Aves	Piciformes	Ramphastidae	*Aulacorhynchus coeruleicinctis*	Annonaceae	*Guatteria glauca*
VELHO	VELHO	India	Oriental	Aves	Columbiformes	Columbidae	*Ducula aenea*	Annonaceae	*Polyalthia simiarum*
VELHO	VELHO	India	Oriental	Aves	Passeriformes	Sturnidae	*Gracula religiosa*	Annonaceae	*Polyalthia simiarum*
VELHO	VELHO	India	Oriental	Aves	Piciformes	Megalaimidae	*Megalaima lineata*	Annonaceae	*Polyalthia simiarum*
VELHO	VELHO	India	Oriental	Aves	Columbiformes	Columbidae	*Ducula badia*	Annonaceae	*Polyalthia simiarum*
VELHO	VELHO	India	Oriental	Aves	Bucerotiformes	Bucerotidae	*Anthracoceros albirostris*	Annonaceae	*Polyalthia simiarum*
VELHO	VELHO	India	Oriental	Aves	Bucerotiformes	Bucerotidae	*Rhyticeros undulatus*	Annonaceae	*Polyalthia simiarum*
VELHO	VELHO2	India	Oriental	Aves	Columbiformes	Columbidae	*Ducula aenea*	Annonaceae	*Polyalthia simiarum*
VELHO	VELHO2	India	Oriental	Aves	Passeriformes	Sturnidae	*Gracula religiosa*	Annonaceae	*Polyalthia simiarum*
VELHO	VELHO2	India	Oriental	Aves	Columbiformes	Columbidae	*Ducula badia*	Annonaceae	*Polyalthia simiarum*

VELHO	VELHO2	India	Oriental	Aves	Bucerotiformes	Bucerotidae	*Anthracoceros albirostris*	Annonaceae	*Polyalthia simiarum*
VELHO	VELHO2	India	Oriental	Aves	Bucerotiformes	Bucerotidae	*Rhyticeros undulatus*	Annonaceae	*Polyalthia simiarum*
Bollen 2000	Bollen 2000	Madagascar	Madagascan	Aves	Columbiformes	Columbidae	*Alectroenas madagascariensis*	Annonaceae	*Fenerivia madagascariensis*
Bollen 2000	Bollen 2000	Madagascar	Madagascan	Aves	Passeriformes	Pycnonotidae	*Hypsipetes madagascariensis*	Annonaceae	*Fenerivia madagascariensis*
Bollen 2000	Bollen 2000	Madagascar	Madagascan	Mammalia	Primates	Lemuridae	*Eulemur collaris*	Annonaceae	*Fenerivia madagascariensis*
Bollen 2000	Bollen 2000	Madagascar	Madagascan	Mammalia	Primates	Cheirogaleidae	*Cheirogaleus major*	Annonaceae	*Fenerivia madagascariensis*
Bollen 2000	Bollen 2000	Madagascar	Madagascan	Mammalia	Primates	Cheirogaleidae	*Microcebus rufus*	Annonaceae	*Fenerivia madagascariensis*
Castro 2015	Castro 2015	Brazil	South American	Aves	Piciformes	Ramphastidae	*Ramphastos vitellinus*	Annonaceae	*Rollinia sericea*
Castro 2015	Castro 2015	Brazil	South American	Aves	Piciformes	Ramphastidae	*Selenidera maculirostris*	Annonaceae	*Rollinia sericea*
Castro 2015	Castro 2015	Brazil	South American	Aves	Passeriformes	Turdidae	*Turdus albicollis*	Annonaceae	*Xylopia langsdorfiana*
Castro 2015	Castro 2015	Brazil	South American	Aves	Passeriformes	Turdidae	*Turdus amaurochalinus*	Annonaceae	*Xylopia langsdorfiana*
Chapman 1996	Chapman 1996	Uganda	African	Mammalia	Primates	Cercopithecidae	*Cercopithecus ascanius*	Annonaceae	*Uvariopsis congensis*
Chapman 1996	Chapman 1996	Uganda	African	Mammalia	Primates	Cercopithecidae	*Cercopithecus mitis*	Annonaceae	*Uvariopsis congensis*
Chapman 1996	Chapman 1996	Uganda	African	Mammalia	Primates	Cercopithecidae	*Lophocebus albigena*	Annonaceae	*Uvariopsis congensis*
Correa 2016	Correa 2016 Apaporis	Colombia	Amazonian	Actinopteri	Characiformes	Bryconidae	*Brycon melanopterus*	Annonaceae	*Annona hypoglauca*
Correa 2016	Correa 2016 Apaporis	Colombia	Amazonian	Actinopteri	Characiformes	Serrasalmidae	*Myloplus asterias*	Annonaceae	*Annona hypoglauca*
Correa	Correa 2016	Colombia	Amazonian	Actinopteri	Characiformes	Serrasalmidae	*Myloplus rubripinnis*	Annonaceae	*Annona*

2016	Apaporis								*hypoglauca*
Correa 2016	Correa 2016 Caqueta	Colombia	Amazonian	Actinopteri	Characiformes	Bryconidae	*Brycon falcatus*	Annonaceae	*Oxandra leucodermis*
Correa 2016	Correa 2016 Caqueta	Colombia	Amazonian	Actinopteri	Characiformes	Bryconidae	*Brycon melanopterus*	Annonaceae	*Oxandra leucodermis*
Correa 2016	Correa 2016 Caqueta	Colombia	Amazonian	Actinopteri	Characiformes	Serrasalmidae	*Myloplus torquatus*	Annonaceae	*Annona hypoglauca*
Dehling 2017	Dehling 2017 San Pedro 1	Peru	Amazonian	Aves	Psittaciformes	Psittacidae	*Aratinga leucophthalma*	Annonaceae	*Guatteria terminalis*
Dehling 2017	Dehling 2017 San Pedro 1	Peru	Amazonian	Aves	Galliformes	Cracidae	*Penelope montagnii*	Annonaceae	*Guatteria terminalis*
Dehling 2017	Dehling 2017 San Pedro 3	Peru	Amazonian	Aves	Passeriformes	Cotingidae	*Rupicola peruvianus*	Annonaceae	*Guatteria duodecima*
Dehling 2017	Dehling 2017 San Pedro 4	Peru	Amazonian	Aves	Trogoniformes	Trogonidae	*Pharomachrus antisianus*	Annonaceae	*Guatteria duodecima*
Donatti 2011	Donatti 2011	Brazil	South American	Mammalia	Carnivora	Canidae	*Cerdocyon thous*	Annonaceae	*Annona dioica*
Donatti 2011	Donatti 2011	Brazil	South American	Testudines	Testudines	Testudinidae	*Chelonoidis carbonaria*	Annonaceae	*Annona dioica*
Donatti 2011	Donatti 2011	Brazil	South American	Mammalia	Perissodactyla	Tapiridae	*Tapirus terrestris*	Annonaceae	*Annona dioica*
Donatti 2011	Donatti 2011	Brazil	South American	Mammalia	Artiodactyla	Tayassuidae	*Tayassu pecari*	Annonaceae	*Annona dioica*
Dos Reis 1983	Dos Reis 1983 Cult et form sec	Brazil	Amazonian	Mammalia	Chiroptera	Phyllostomidae	*Phyllostomus elongatus*	Annonaceae	*Rollinia mucosa*
Dubost 1984	Dubost 1984	Gabon	Guineo-Congolian	Mammalia	Artiodactyla	Bovidae	*Philantomba monticola*	Annonaceae	*Artabotrys le-testui*
Dubost 1984	Dubost 1984	Gabon	Guineo-Congolian	Mammalia	Artiodactyla	Bovidae	*Philantomba monticola*	Annonaceae	*Annickia polycarpa*

Dubost 1984	Dubost 1984	Gabon	Guineo-Congolian	Mammalia	Artiodactyla	Bovidae	*Philantomba monticola*	Annonaceae	*Greenwayodendron suaveolens*
Dubost 1984	Dubost 1984	Gabon	Guineo-Congolian	Mammalia	Artiodactyla	Bovidae	*Philantomba monticola*	Annonaceae	*Uvaria klaineana*
Dubost 1984	Dubost 1984	Gabon	Guineo-Congolian	Mammalia	Artiodactyla	Bovidae	*Philantomba monticola*	Annonaceae	*Xylopia hypolampra*
Dubost 1984	Dubost 1984	Gabon	Guineo-Congolian	Mammalia	Artiodactyla	Bovidae	*Philantomba monticola*	Annonaceae	*Xylopia staudtii*
Dubost 1984	Dubost 1984	Gabon	Guineo-Congolian	Mammalia	Artiodactyla	Tragulidae	*Hyemoschus aquaticus*	Annonaceae	*Artabotrys le-testui*
Dubost 1984	Dubost 1984	Gabon	Guineo-Congolian	Mammalia	Artiodactyla	Tragulidae	*Hyemoschus aquaticus*	Annonaceae	*Greenwayodendron suaveolens*
Dubost 1984	Dubost 1984	Gabon	Guineo-Congolian	Mammalia	Artiodactyla	Tragulidae	*Hyemoschus aquaticus*	Annonaceae	*Xylopia aethiopica*
Dubost 1984	Dubost 1984	Gabon	Guineo-Congolian	Mammalia	Artiodactyla	Tragulidae	*Hyemoschus aquaticus*	Annonaceae	*Xylopia hypolampra*
Dubost 1984	Dubost 1984	Gabon	Guineo-Congolian	Mammalia	Artiodactyla	Bovidae	*Cephalophus leucogaster*	Annonaceae	*Artabotrys le-testui*
Dubost 1984	Dubost 1984	Gabon	Guineo-Congolian	Mammalia	Artiodactyla	Bovidae	*Cephalophus leucogaster*	Annonaceae	*Artabotrys congolensis*
Dubost 1984	Dubost 1984	Gabon	Guineo-Congolian	Mammalia	Artiodactyla	Bovidae	*Cephalophus leucogaster*	Annonaceae	*Greenwayodendron suaveolens*
Dubost 1984	Dubost 1984	Gabon	Guineo-Congolian	Mammalia	Artiodactyla	Bovidae	*Cephalophus nigrifrons*	Annonaceae	*Artabotrys congolensis*
Dubost 1984	Dubost 1984	Gabon	Guineo-Congolian	Mammalia	Artiodactyla	Bovidae	*Cephalophus nigrifrons*	Annonaceae	*Greenwayodendron suaveolens*
Dubost 1984	Dubost 1984	Gabon	Guineo-Congolian	Mammalia	Artiodactyla	Bovidae	*Cephalophus callipygus*	Annonaceae	*Artabotrys le-testui*
Dubost 1984	Dubost 1984	Gabon	Guineo-Congolian	Mammalia	Artiodactyla	Bovidae	*Cephalophus callipygus*	Annonaceae	*Greenwayodendron suaveolens*
Dubost 1984	Dubost 1984	Gabon	Guineo-Congolian	Mammalia	Artiodactyla	Bovidae	*Cephalophus callipygus*	Annonaceae	*Uvaria klaineana*
Dubost	Dubost 1984	Gabon	Guineo-	Mammalia	Artiodactyla	Bovidae	*Cephalophus*	Annonaceae	*Xylopia*

1984			Congolian				*callipygus*		*hypolampra*
Dubost 1984	Dubost 1984	Gabon	Guineo-Congolian	Mammalia	Artiodactyla	Bovidae	*Cephalophus callipygus*	Annonaceae	*Xylopia staudtii*
Dubost 1984	Dubost 1984	Gabon	Guineo-Congolian	Mammalia	Artiodactyla	Bovidae	*Cephalophus dorsalis*	Annonaceae	*Anonidium mannii*
Dubost 1984	Dubost 1984	Gabon	Guineo-Congolian	Mammalia	Artiodactyla	Bovidae	*Cephalophus dorsalis*	Annonaceae	*Artabotrys le-testui*
Dubost 1984	Dubost 1984	Gabon	Guineo-Congolian	Mammalia	Artiodactyla	Bovidae	*Cephalophus dorsalis*	Annonaceae	*Greenwayodendron suaveolens*
Dubost 1984	Dubost 1984	Gabon	Guineo-Congolian	Mammalia	Artiodactyla	Bovidae	*Cephalophus dorsalis*	Annonaceae	*Uvaria klaineana*
Dubost 1984	Dubost 1984	Gabon	Guineo-Congolian	Mammalia	Artiodactyla	Bovidae	*Cephalophus dorsalis*	Annonaceae	*Xylopia hypolampra*
Dubost 1984	Dubost 1984	Gabon	Guineo-Congolian	Mammalia	Artiodactyla	Bovidae	*Cephalophus dorsalis*	Annonaceae	*Xylopia staudtii*
Dubost 1984	Dubost 1984	Gabon	Guineo-Congolian	Mammalia	Artiodactyla	Bovidae	*Cephalophus silvicultor*	Annonaceae	*Greenwayodendron suaveolens*
Elder 2013	Elder 2013	Indonesia	Indo-Malayan	Mammalia	Primates	Hylobatidae	*Symphalangus syndactylus*	Annonaceae	*Polyalthia lateriflora*
Elder 2013	Elder 2013	Indonesia	Indo-Malayan	Mammalia	Primates	Hylobatidae	*Symphalangus syndactylus*	Annonaceae	*Polyalthia rumphii*
Elder 2013	Elder 2013	Indonesia	Indo-Malayan	Mammalia	Primates	Hylobatidae	*Symphalangus syndactylus*	Annonaceae	*Miliusa horsfieldii*
Elder 2013	Elder 2013	Indonesia	Indo-Malayan	Mammalia	Primates	Hylobatidae	*Symphalangus syndactylus*	Annonaceae	*Stelechocarpus burahol*
Elder 2013	Elder 2013	Indonesia	Indo-Malayan	Mammalia	Primates	Hylobatidae	*Hylobates agilis*	Annonaceae	*Polyalthia lateriflora*
Elder 2013	Elder 2013	Indonesia	Indo-Malayan	Mammalia	Primates	Hylobatidae	*Hylobates agilis*	Annonaceae	*Polyalthia rumphii*
Elder 2013	Elder 2013	Indonesia	Indo-Malayan	Mammalia	Primates	Hylobatidae	*Hylobates agilis*	Annonaceae	*Miliusa horsfieldii*
Engel 2000	Engel 2000	Kenya	African	Mammalia	Primates	Cercopithecidae	*Cercopithecus mitis*	Annonaceae	*Monanthotaxis buchananii*

Engel 2000	Engel 2000	Kenya	African	Mammalia	Carnivora	Viverridae	*Civettictis civetta*	Annonaceae	*Annona senegalensis*
Engel 2000	Engel 2000	Kenya	African	Mammalia	Carnivora	Viverridae	*Civettictis civetta*	Annonaceae	*Asteranthe asterias*
Engel 2000	Engel 2000	Kenya	African	Mammalia	Carnivora	Viverridae	*Civettictis civetta*	Annonaceae	*Lettowianthus stellatus*
Engel 2000	Engel 2000	Kenya	African	Mammalia	Carnivora	Viverridae	*Civettictis civetta*	Annonaceae	*Monanthotaxis buchananii*
Engel 2000	Engel 2000	Kenya	African	Mammalia	Carnivora	Viverridae	*Civettictis civetta*	Annonaceae	*Sphaerocoryne gracilis*
Engel 2000	Engel 2000	Kenya	African	Mammalia	Carnivora	Viverridae	*Civettictis civetta*	Annonaceae	*Uvaria acuminata*
Engel 2000	Engel 2000	Kenya	African	Mammalia	Carnivora	Viverridae	*Civettictis civetta*	Annonaceae	*Uvariodendron kirkii*
Engel 2000	Engel 2000	Kenya	African	Mammalia	Rodentia	Nesomyidae	*Cricetomys gambianus*	Annonaceae	*Monanthotaxis buchananii*
Engel 2000	Engel 2000	Kenya	African	Mammalia	Proboscidea	Elephantidae	*Loxodonta africana*	Annonaceae	*Monanthotaxis buchananii*
Engel 2000	Engel 2000	Kenya	African	Mammalia	Proboscidea	Elephantidae	*Loxodonta africana*	Annonaceae	*Uvaria acuminata*
Engel 2000	Engel 2000	Kenya	African	Mammalia	Primates	Galagidae	*Otolemur garnettii*	Annonaceae	*Monanthotaxis buchananii*
Engel 2000	Engel 2000	Kenya	African	Mammalia	Primates	Galagidae	*Otolemur garnettii*	Annonaceae	*Sphaerocoryne gracilis*
Engel 2000	Engel 2000	Kenya	African	Mammalia	Primates	Galagidae	*Otolemur garnettii*	Annonaceae	*Uvaria acuminata*
Engel 2000	Engel 2000	Kenya	African	Mammalia	Primates	Cercopithecidae	*Papio cynocephalus*	Annonaceae	*Uvaria acuminata*
Engel 2000	Engel 2000	Kenya	African	Aves	Bucerotiformes	Bucerotidae	*Tockus alboterminatus*	Annonaceae	*Monanthotaxis fornicata*
Erard 2007	Erard 2007	French Guiana	Amazonian	Aves	Galliformes	Cracidae	*Crax alector*	Annonaceae	*Ephedranthus guianensis*
Erard 2007	Erard 2007	French Guiana	Amazonian	Aves	Galliformes	Cracidae	*Crax alector*	Annonaceae	*Unonopsis guatterioides*
Erard 2007	Erard 2007	French Guiana	Amazonian	Aves	Galliformes	Cracidae	*Crax alector*	Annonaceae	*Xylopia nitida*
Erard 2007	Erard 2007	French	Amazonian	Aves	Tinamiformes	Tinamidae	*Tinamus major*	Annonaceae	*Duguetia eximia*

		Guiana							
Fontanari 2018	Fontanari 2018	Brazil	South American	Aves	Passeriformes	Fringillidae	*Euphonia chalybea*	Annonaceae	*Annona neosalicifolia*
Gautier-Hion 1985	Gautier-Hion 1985	Gabon	Guineo-Congolian	Mammalia	Primates	Cercopithecidae	*Cercopithecus cephus*	Annonaceae	*Anonidium mannii*
Gautier-Hion 1985	Gautier-Hion 1985	Gabon	Guineo-Congolian	Mammalia	Primates	Cercopithecidae	*Lophocebus albigena*	Annonaceae	*Anonidium mannii*
Gautier-Hion 1985	Gautier-Hion 1985	Gabon	Guineo-Congolian	Mammalia	Proboscidea	Elephantidae	*Loxodonta cyclotis*	Annonaceae	*Anonidium mannii*
Guillotin 1994	Guillotin 1994	French Guiana	Amazonian	Mammalia	Primates	Cebidae	*Sapajus apella*	Annonaceae	*Unonopsis guatterioides*
Hasui 1998	Hasui 1998	Brazil	South American	Aves	Passeriformes	Tyrannidae	*Elaenia flavogaster*	Annonaceae	*Rollinia sericea*
Hladik 1969	Hladik 1969	Panama	Panamanian	Mammalia	Primates	Cebidae	*Cebus imitator*	Annonaceae	*Annona spraguei*
Hladik 1969	Hladik 1969	Panama	Panamanian	Mammalia	Primates	Cebidae	*Cebus imitator*	Annonaceae	*Desmopsis panamensis*
Hladik 1969	Hladik 1969	Panama	Panamanian	Mammalia	Primates	Atelidae	*Ateles geoffroyi*	Annonaceae	*Annona spraguei*
Hodgkison 2001	Hodgkison 2001	Malaysia	Indo-Malayan	Mammalia	Chiroptera	Pteropodidae	*Balionycteris maculata*	Annonaceae	*Cyathocalyx pubescens*
Hodgkison 2001	Hodgkison 2001	Malaysia	Indo-Malayan	Mammalia	Chiroptera	Pteropodidae	*Balionycteris maculata*	Annonaceae	*Polyalthia obliqua*
Hodgkison 2001	Hodgkison 2001	Malaysia	Indo-Malayan	Mammalia	Chiroptera	Pteropodidae	*Balionycteris maculata*	Annonaceae	*Pseuduvaria setosa*
Hodgkison 2001	Hodgkison 2001	Malaysia	Indo-Malayan	Mammalia	Chiroptera	Pteropodidae	*Chironax melanocephalus*	Annonaceae	*Polyalthia obliqua*
Hodgkison 2001	Hodgkison 2001	Malaysia	Indo-Malayan	Mammalia	Chiroptera	Pteropodidae	*Cynopterus brachyotis*	Annonaceae	*Cyathocalyx pubescens*
Houle 2010	Houle 2010	Uganda	African	Mammalia	Primates	Cercopithecidae	*Cercopithecus ascanius*	Annonaceae	*Uvariopsis congensis*
Houle 2010	Houle 2010	Uganda	African	Mammalia	Primates	Cercopithecidae	*Cercopithecus mitis*	Annonaceae	*Uvariopsis congensis*

Houle 2010	Houle 2010	Uganda	African	Mammalia	Primates	Cercopithecidae	*Lophocebus albigena*	Annonaceae	*Uvariopsis congensis*
Houle 2010	Houle 2010	Uganda	African	Mammalia	Primates	Hominidae	*Pan troglodytes*	Annonaceae	*Monodora myristica*
Houle 2010	Houle 2010	Uganda	African	Mammalia	Primates	Hominidae	*Pan troglodytes*	Annonaceae	*Uvariopsis congensis*
Kitamura 2002	Kitamura 2002	Thailand	Oriental	Mammalia	Proboscidea	Elephantidae	*Elephas maximus*	Annonaceae	*Platymitra macrocarpa*
Kone 2008	Kone 2008	Côte d'Ivoire	Guineo-Congolian	Mammalia	Primates	Cercopithecidae	*Cercopithecus lowei*	Annonaceae	*Xylopia villosa*
Kone 2008	Kone 2008	Côte d'Ivoire	Guineo-Congolian	Mammalia	Primates	Cercopithecidae	*Cercopithecus diana*	Annonaceae	*Piptostigma fasciculatum*
Kone 2008	Kone 2008	Côte d'Ivoire	Guineo-Congolian	Mammalia	Primates	Cercopithecidae	*Cercopithecus diana*	Annonaceae	*Xylopia aethiopica*
Kone 2008	Kone 2008	Côte d'Ivoire	Guineo-Congolian	Mammalia	Primates	Cercopithecidae	*Cercopithecus diana*	Annonaceae	*Xylopia parviflora*
Kone 2008	Kone 2008	Côte d'Ivoire	Guineo-Congolian	Mammalia	Primates	Cercopithecidae	*Cercopithecus diana*	Annonaceae	*Xylopia quintasii*
Kone 2008	Kone 2008	Côte d'Ivoire	Guineo-Congolian	Mammalia	Primates	Cercopithecidae	*Cercopithecus diana*	Annonaceae	*Xylopia villosa*
Kone 2008	Kone 2008	Côte d'Ivoire	Guineo-Congolian	Mammalia	Primates	Cercopithecidae	*Cercopithecus petaurista*	Annonaceae	*Xylopia quintasii*
Kone 2008	Kone 2008	Côte d'Ivoire	Guineo-Congolian	Mammalia	Primates	Cercopithecidae	*Cercopithecus petaurista*	Annonaceae	*Xylopia villosa*
Kone 2008	Kone 2008	Côte d'Ivoire	Guineo-Congolian	Mammalia	Primates	Cercopithecidae	*Cercocebus atys*	Annonaceae	*Duguetia staudtii*
Kone 2008	Kone 2008	Côte d'Ivoire	Guineo-Congolian	Mammalia	Primates	Cercopithecidae	*Cercocebus atys*	Annonaceae	*Xylopia quintasii*
Kone 2008	Kone 2008	Côte d'Ivoire	Guineo-Congolian	Mammalia	Primates	Cercopithecidae	*Piliocolobus badius*	Annonaceae	*Duguetia staudtii*
Kone 2008	Kone 2008	Côte d'Ivoire	Guineo-Congolian	Mammalia	Primates	Cercopithecidae	*Piliocolobus badius*	Annonaceae	*Xylopia villosa*
Kone 2008	Kone 2008	Côte	Guineo-	Mammalia	Primates	Cercopithecidae	*Colobus polykomos*	Annonaceae	*Xylopia acutiflora*

		d'Ivoire	Congolian						
Kone 2008	Kone 2008	Côte d'Ivoire	Guineo-Congolian	Mammalia	Primates	Cercopithecidae	*Colobus polykomos*	Annonaceae	*Xylopia quintasii*
Llewellyn 1952	Llewellyn 1952	USA	North American	Mammalia	Carnivora	Procyonidae	*Procyon lotor*	Annonaceae	*Asimina triloba*
Mackinnon 1980	Mackinnon 1980	Malaysia	Indo-Malayan	Mammalia	Primates	Hylobatidae	*Symphalangus syndactylus*	Annonaceae	*Xylopia magna*
Mackinnon 1980	Mackinnon 1980	Malaysia	Indo-Malayan	Mammalia	Primates	Hylobatidae	*Hylobates lar*	Annonaceae	*Xylopia magna*
Mackinnon 1980	Mackinnon 1980	Malaysia	Indo-Malayan	Mammalia	Primates	Cercopithecidae	*Macaca fascicularis*	Annonaceae	*Xylopia magna*
Mackinnon 1980	Mackinnon 1980	Malaysia	Indo-Malayan	Mammalia	Primates	Cercopithecidae	*Trachypithecus obscurus*	Annonaceae	*Xylopia magna*
Mackinnon 1980	Mackinnon 1980	Malaysia	Indo-Malayan	Mammalia	Primates	Cercopithecidae	*Presbytis siamensis*	Annonaceae	*Xylopia magna*
Montalvan 2015	Montalvan 2015	Peru	Amazonian	Mammalia	Primates	Pitheciidae	*Plecturocebus discolor*	Annonaceae	*Xylopia aromatica*
Montalvan 2015	Montalvan 2015	Peru	Amazonian	Mammalia	Primates	Pitheciidae	*Cheracebus lucifer*	Annonaceae	*Guatteria longicuspis*
Montalvan 2015	Montalvan 2015	Peru	Amazonian	Mammalia	Primates	Pitheciidae	*Pithecia aequatorialis*	Annonaceae	*Diclinanona calycina*
Montalvan 2015	Montalvan 2015	Peru	Amazonian	Mammalia	Primates	Pitheciidae	*Pithecia aequatorialis*	Annonaceae	*Oxandra xylopioides*
Montalvan 2015	Montalvan 2015	Peru	Amazonian	Mammalia	Primates	Pitheciidae	*Pithecia aequatorialis*	Annonaceae	*Xylopia aromatica*
Montalvan 2015	Montalvan 2015	Peru	Amazonian	Mammalia	Carnivora	Procyonidae	*Potos flavus*	Annonaceae	*Rollinia cuspidata*
Ortiz-Pulido 2000	Ortiz-Pulido 2000	Mexico	Mexican	Aves	Trogoniformes	Trogonidae	*Trogon melanocephalus*	Annonaceae	*Sapranthus microcarpus*
Palacios 2016	Palacios 2016	Colombia	Amazonian	Aves	Piciformes	Ramphastidae	*Aulacorhynchus haematopygus*	Annonaceae	*Guatteria pilosula*
Palacios	Palacios	Colombia	Amazonian	Aves	Piciformes	Ramphastidae	*Aulacorhynchus*	Annonaceae	*Guatteria pilosula*

2016	2016						*prasinus*		
Palacios 2016	Palacios 2016	Colombia	Amazonian	Aves	Galliformes	Cracidae	*Chamaepetes goudotii*	Annonaceae	*Guatteria pilosula*
Palacios 2016	Palacios 2016	Colombia	Amazonian	Aves	Trogoniformes	Trogonidae	*Pharomachrus auriceps*	Annonaceae	*Guatteria pilosula*
Poulsen 2001 2002 primates	Poulsen 2001 2002 primates	Cameroon	Guineo-Congolian	Mammalia	Primates	Cercopithecidae	*Lophocebus albigena*	Annonaceae	*Anonidium mannii*
Poulsen 2001 2002 primates	Poulsen 2001 2002 primates	Cameroon	Guineo-Congolian	Mammalia	Primates	Cercopithecidae	*Lophocebus albigena*	Annonaceae	*Cleistopholis glauca*
Poulsen 2001 2002 primates	Poulsen 2001 2002 primates	Cameroon	Guineo-Congolian	Mammalia	Primates	Cercopithecidae	*Lophocebus albigena*	Annonaceae	*Annickia chlorantha*
Poulsen 2001 2002 primates	Poulsen 2001 2002 primates	Cameroon	Guineo-Congolian	Mammalia	Primates	Cercopithecidae	*Lophocebus albigena*	Annonaceae	*Duguetia staudtii*
Poulsen 2001 2002 primates	Poulsen 2001 2002 primates	Cameroon	Guineo-Congolian	Mammalia	Primates	Cercopithecidae	*Lophocebus albigena*	Annonaceae	*Greenwayodendron suaveolens*
Poulsen 2001 2002 primates	Poulsen 2001 2002 primates	Cameroon	Guineo-Congolian	Mammalia	Primates	Cercopithecidae	*Lophocebus albigena*	Annonaceae	*Xylopia aethiopica*
Poulsen 2001 2002 primates	Poulsen 2001 2002 primates	Cameroon	Guineo-Congolian	Mammalia	Primates	Cercopithecidae	*Lophocebus albigena*	Annonaceae	*Xylopia hypolampra*
Poulsen 2001 2002 primates	Poulsen 2001 2002 primates	Cameroon	Guineo-Congolian	Mammalia	Primates	Cercopithecidae	*Lophocebus albigena*	Annonaceae	*Xylopia parviflora*
Poulsen 2001 2002 primates	Poulsen 2001 2002 primates	Cameroon	Guineo-Congolian	Mammalia	Primates	Cercopithecidae	*Lophocebus albigena*	Annonaceae	*Xylopia quintasii*
Poulsen 2001 2002	Poulsen 2001 2002	Cameroon	Guineo-Congolian	Mammalia	Primates	Cercopithecidae	*Lophocebus albigena*	Annonaceae	*Xylopia rubescens*

primates	primates								
Poulsen 2001 2002 primates	Poulsen 2001 2002 primates	Cameroon	Guineo-Congolian	Mammalia	Primates	Cercopithecidae	*Lophocebus albigena*	Annonaceae	*Xylopia staudtii*
Poulsen 2001 2002 primates	Poulsen 2001 2002 primates	Cameroon	Guineo-Congolian	Mammalia	Primates	Cercopithecidae	*Cercopithecus nictitans*	Annonaceae	*Cleistopholis glauca*
Poulsen 2001 2002 primates	Poulsen 2001 2002 primates	Cameroon	Guineo-Congolian	Mammalia	Primates	Cercopithecidae	*Cercopithecus nictitans*	Annonaceae	*Annickia chlorantha*
Poulsen 2001 2002 primates	Poulsen 2001 2002 primates	Cameroon	Guineo-Congolian	Mammalia	Primates	Cercopithecidae	*Cercopithecus nictitans*	Annonaceae	*Hexalobus crispiflorus*
Poulsen 2001 2002 primates	Poulsen 2001 2002 primates	Cameroon	Guineo-Congolian	Mammalia	Primates	Cercopithecidae	*Cercopithecus nictitans*	Annonaceae	*Duguetia staudtii*
Poulsen 2001 2002 primates	Poulsen 2001 2002 primates	Cameroon	Guineo-Congolian	Mammalia	Primates	Cercopithecidae	*Cercopithecus nictitans*	Annonaceae	*Greenwayodendron suaveolens*
Poulsen 2001 2002 primates	Poulsen 2001 2002 primates	Cameroon	Guineo-Congolian	Mammalia	Primates	Cercopithecidae	*Cercopithecus nictitans*	Annonaceae	*Uvariastrum pierreanum*
Poulsen 2001 2002 primates	Poulsen 2001 2002 primates	Cameroon	Guineo-Congolian	Mammalia	Primates	Cercopithecidae	*Cercopithecus nictitans*	Annonaceae	*Xylopia aethiopica*
Poulsen 2001 2002 primates	Poulsen 2001 2002 primates	Cameroon	Guineo-Congolian	Mammalia	Primates	Cercopithecidae	*Cercopithecus nictitans*	Annonaceae	*Xylopia hypolampra*
Poulsen 2001 2002 primates	Poulsen 2001 2002 primates	Cameroon	Guineo-Congolian	Mammalia	Primates	Cercopithecidae	*Cercopithecus nictitans*	Annonaceae	*Xylopia rubescens*
Poulsen 2001 2002 primates	Poulsen 2001 2002 primates	Cameroon	Guineo-Congolian	Mammalia	Primates	Cercopithecidae	*Cercopithecus nictitans*	Annonaceae	*Xylopia staudtii*

Poulsen 2001 2002 primates	Poulsen 2001 2002 primates	Cameroon	Guineo-Congolian	Mammalia	Primates	Cercopithecidae	*Cercopithecus pogonias*	Annonaceae	*Cleistopholis glauca*
Poulsen 2001 2002 primates	Poulsen 2001 2002 primates	Cameroon	Guineo-Congolian	Mammalia	Primates	Cercopithecidae	*Cercopithecus pogonias*	Annonaceae	*Annickia chlorantha*
Poulsen 2001 2002 primates	Poulsen 2001 2002 primates	Cameroon	Guineo-Congolian	Mammalia	Primates	Cercopithecidae	*Cercopithecus pogonias*	Annonaceae	*Duguetia staudtii*
Poulsen 2001 2002 primates	Poulsen 2001 2002 primates	Cameroon	Guineo-Congolian	Mammalia	Primates	Cercopithecidae	*Cercopithecus pogonias*	Annonaceae	*Greenwayodendron suaveolens*
Poulsen 2001 2002 primates	Poulsen 2001 2002 primates	Cameroon	Guineo-Congolian	Mammalia	Primates	Cercopithecidae	*Cercopithecus pogonias*	Annonaceae	*Xylopia aethiopica*
Poulsen 2001 2002 primates	Poulsen 2001 2002 primates	Cameroon	Guineo-Congolian	Mammalia	Primates	Cercopithecidae	*Cercopithecus pogonias*	Annonaceae	*Xylopia hypolampra*
Poulsen 2001 2002 primates	Poulsen 2001 2002 primates	Cameroon	Guineo-Congolian	Mammalia	Primates	Cercopithecidae	*Cercopithecus pogonias*	Annonaceae	*Xylopia rubescens*
Poulsen 2001 2002 primates	Poulsen 2001 2002 primates	Cameroon	Guineo-Congolian	Mammalia	Primates	Cercopithecidae	*Cercopithecus pogonias*	Annonaceae	*Xylopia staudtii*
Poulsen 2001 2002 primates	Poulsen 2001 2002 primates	Cameroon	Guineo-Congolian	Mammalia	Primates	Cercopithecidae	*Cercopithecus cephus*	Annonaceae	*Cleistopholis glauca*
Poulsen 2001 2002 primates	Poulsen 2001 2002 primates	Cameroon	Guineo-Congolian	Mammalia	Primates	Cercopithecidae	*Cercopithecus cephus*	Annonaceae	*Annickia chlorantha*
Poulsen 2001 2002 primates	Poulsen 2001 2002 primates	Cameroon	Guineo-Congolian	Mammalia	Primates	Cercopithecidae	*Cercopithecus cephus*	Annonaceae	*Greenwayodendron suaveolens*
Poulsen 2001 2002	Poulsen 2001 2002	Cameroon	Guineo-Congolian	Mammalia	Primates	Cercopithecidae	*Cercopithecus cephus*	Annonaceae	*Xylopia hypolampra*

primates	primates								
Poulsen 2001 2002 primates	Poulsen 2001 2002 primates	Cameroon	Guineo-Congolian	Mammalia	Primates	Cercopithecidae	*Colobus guereza*	Annonaceae	*Cleistopholis glauca*
Poulsen 2001 2002 primates	Poulsen 2001 2002 primates	Cameroon	Guineo-Congolian	Mammalia	Primates	Cercopithecidae	*Colobus guereza*	Annonaceae	*Annickia chlorantha*
Poulsen 2001 2002 primates	Poulsen 2001 2002 primates	Cameroon	Guineo-Congolian	Mammalia	Primates	Cercopithecidae	*Colobus guereza*	Annonaceae	*Greenwayodendron suaveolens*
Poulsen 2001 2002 primates	Poulsen 2001 2002 primates	Cameroon	Guineo-Congolian	Mammalia	Primates	Cercopithecidae	*Colobus guereza*	Annonaceae	*Xylopia hypolampra*
Poulsen 2001 2002 primates	Poulsen 2001 2002 primates	Cameroon	Guineo-Congolian	Mammalia	Primates	Cercopithecidae	*Colobus guereza*	Annonaceae	*Xylopia rubescens*
Poulsen 2001 2002 primates	Poulsen 2001 2002 primates	Cameroon	Guineo-Congolian	Mammalia	Primates	Hominidae	*Pan troglodytes*	Annonaceae	*Annickia chlorantha*
Poulsen 2001 2002 primates	Poulsen 2001 2002 primates	Cameroon	Guineo-Congolian	Mammalia	Primates	Hominidae	*Gorilla gorilla*	Annonaceae	*Duguetia staudtii*
Purificacao 2014	Purificacao 2014 forest rainy	Brazil	Amazonian	Aves	Passeriformes	Thraupidae	*Dacnis cayana*	Annonaceae	*Xylopia aromatica*
Purificacao 2014	Purificacao 2014 forest dry	Brazil	Amazonian	Aves	Piciformes	Picidae	*Celeus flavescens*	Annonaceae	*Xylopia aromatica*
Purificacao 2014	Purificacao 2014 forest dry	Brazil	Amazonian	Aves	Piciformes	Picidae	*Celeus flavescens*	Annonaceae	*Xylopia sericea*
Purificacao 2014	Purificacao 2014 forest dry	Brazil	Amazonian	Aves	Passeriformes	Fringillidae	*Cyanerpes cyaneus*	Annonaceae	*Xylopia sericea*

Purificacao 2014	Purificacao 2014 forest dry	Brazil	Amazonian	Aves	Passeriformes	Thraupidae	*Dacnis cayana*	Annonaceae	*Xylopia aromatica*
Purificacao 2014	Purificacao 2014 forest dry	Brazil	Amazonian	Aves	Passeriformes	Thraupidae	*Dacnis cayana*	Annonaceae	*Xylopia sericea*
Purificacao 2014	Purificacao 2014 forest dry	Brazil	Amazonian	Aves	Passeriformes	Tyrannidae	*Elaenia mesoleuca*	Annonaceae	*Xylopia sericea*
Purificacao 2014	Purificacao 2014 forest dry	Brazil	Amazonian	Aves	Passeriformes	Thraupidae	*Hemithraupis guira*	Annonaceae	*Xylopia sericea*
Purificacao 2014	Purificacao 2014 forest dry	Brazil	Amazonian	Aves	Coraciiformes	Momotidae	*Momotus momota*	Annonaceae	*Xylopia aromatica*
Purificacao 2014	Purificacao 2014 forest dry	Brazil	Amazonian	Aves	Passeriformes	Thraupidae	*Ramphocelus carbo*	Annonaceae	*Xylopia aromatica*
Purificacao 2014	Purificacao 2014 forest dry	Brazil	Amazonian	Aves	Passeriformes	Thraupidae	*Thraupis sayaca*	Annonaceae	*Xylopia sericea*
Purificacao 2014	Purificacao 2014 forest dry	Brazil	Amazonian	Aves	Passeriformes	Tyrannidae	*Tityra semifasciata*	Annonaceae	*Xylopia aromatica*
Purificacao 2014	Purificacao 2014 forest dry	Brazil	Amazonian	Aves	Passeriformes	Turdidae	*Turdus leucomelas*	Annonaceae	*Xylopia aromatica*
Purificacao 2014	Purificacao 2014 forest dry	Brazil	Amazonian	Aves	Passeriformes	Turdidae	*Turdus leucomelas*	Annonaceae	*Xylopia sericea*
Purificacao 2014	Purificacao 2014 savannah rainy	Brazil	Amazonian	Aves	Passeriformes	Thraupidae	*Dacnis cayana*	Annonaceae	*Xylopia aromatica*
Purificacao	Purificacao	Brazil	Amazonian	Aves	Passeriformes	Thraupidae	*Dacnis cayana*	Annonaceae	*Xylopia*

2014	2014 savannah dry								*aromatica*
Purificacao 2014	Purificacao 2014 savannah dry	Brazil	Amazonian	Aves	Passeriformes	Tyrannidae	*Elaenia flavogaster*	Annonaceae	*Xylopia aromatica*
Razafindra tsima 2013	Razafindratsi ma 2013	Madagascar	Madagascan	Mammalia	Primates	Lemuridae	*Eulemur rubriventer*	Annonaceae	*Xylopia buxifolia*
Razafindra tsima 2013	Razafindratsi ma 2013	Madagascar	Madagascan	Mammalia	Primates	Lemuridae	*Varecia variegata*	Annonaceae	*Ambavia capuronii*
Sethi 2012	Sethi 2012	India	Oriental	Aves	Bucerotiformes	Bucerotidae	*Anthracoceros albirostris*	Annonaceae	*Polyalthia simiarum*
Sethi 2012	Sethi 2012	India	Oriental	Aves	Bucerotiformes	Bucerotidae	*Buceros bicornis*	Annonaceae	*Polyalthia simiarum*
Sethi 2012	Sethi 2012	India	Oriental	Mammalia	Rodentia	Sciuridae	*Callosciurus erythraeus*	Annonaceae	*Polyalthia simiarum*
Sethi 2012	Sethi 2012	India	Oriental	Mammalia	Rodentia	Sciuridae	*Callosciurus pygerythrus*	Annonaceae	*Polyalthia simiarum*
Sethi 2012	Sethi 2012	India	Oriental	Aves	Columbiformes	Columbidae	*Ducula badia*	Annonaceae	*Polyalthia simiarum*
Sethi 2012	Sethi 2012	India	Oriental	Aves	Passeriformes	Sturnidae	*Gracula religiosa*	Annonaceae	*Polyalthia simiarum*
Sethi 2012	Sethi 2012	India	Oriental	Mammalia	Primates	Cercopithecidae	*Macaca mulatta*	Annonaceae	*Polyalthia simiarum*
Sethi 2012	Sethi 2012	India	Oriental	Mammalia	Rodentia	Sciuridae	*Ratufa bicolor*	Annonaceae	*Polyalthia simiarum*
Sethi 2012	Sethi 2012	India	Oriental	Aves	Bucerotiformes	Bucerotidae	*Rhyticeros undulatus*	Annonaceae	*Polyalthia simiarum*
Simmen 1996	Simmen 1996	French Guiana	Amazonian	Mammalia	Primates	Atelidae	*Ateles paniscus*	Annonaceae	*Duguetia surinamensis*
Stevenson 2015	Stevenson 2015	Colombia	Amazonian	Aves	Galliformes	Cracidae	*Aburria cumanensis*	Annonaceae	*Guatteria punctata*
Stevenson 2015	Stevenson 2015	Colombia	Amazonian	Aves	Galliformes	Cracidae	*Aburria cumanensis*	Annonaceae	*Oxandra mediocris*

Stevenson 2015	Stevenson 2015	Colombia	Amazonian	Mammalia	Primates	Atelidae	*Ateles belzebuth*	Annonaceae	*Guatteria punctata*
Stevenson 2015	Stevenson 2015	Colombia	Amazonian	Mammalia	Primates	Atelidae	*Ateles belzebuth*	Annonaceae	*Oxandra mediocris*
Stevenson 2015	Stevenson 2015	Colombia	Amazonian	Mammalia	Primates	Atelidae	*Ateles belzebuth*	Annonaceae	*Malmea dielsiana*
Stevenson 2015	Stevenson 2015	Colombia	Amazonian	Aves	Passeriformes	Icteridae	*Cacicus cela*	Annonaceae	*Guatteria punctata*
Stevenson 2015	Stevenson 2015	Colombia	Amazonian	Aves	Piciformes	Ramphastidae	*Capito auratus*	Annonaceae	*Oxandra mediocris*
Stevenson 2015	Stevenson 2015	Colombia	Amazonian	Mammalia	Primates	Cebidae	*Sapajus apella*	Annonaceae	*Guatteria punctata*
Stevenson 2015	Stevenson 2015	Colombia	Amazonian	Mammalia	Primates	Cebidae	*Sapajus apella*	Annonaceae	*Oxandra mediocris*
Stevenson 2015	Stevenson 2015	Colombia	Amazonian	Mammalia	Primates	Cebidae	*Sapajus apella*	Annonaceae	*Malmea dielsiana*
Stevenson 2015	Stevenson 2015	Colombia	Amazonian	Mammalia	Primates	Atelidae	*Lagothrix lagothricha*	Annonaceae	*Guatteria punctata*
Stevenson 2015	Stevenson 2015	Colombia	Amazonian	Mammalia	Primates	Atelidae	*Lagothrix lagothricha*	Annonaceae	*Oxandra mediocris*
Stevenson 2015	Stevenson 2015	Colombia	Amazonian	Mammalia	Primates	Atelidae	*Lagothrix lagothricha*	Annonaceae	*Malmea dielsiana*
Stevenson 2015	Stevenson 2015	Colombia	Amazonian	Aves	Galliformes	Cracidae	*Penelope jacquacu*	Annonaceae	*Guatteria punctata*
Stevenson 2015	Stevenson 2015	Colombia	Amazonian	Aves	Piciformes	Ramphastidae	*Pteroglossus castanotis*	Annonaceae	*Oxandra mediocris*
Stevenson 2015	Stevenson 2015	Colombia	Amazonian	Aves	Piciformes	Ramphastidae	*Pteroglossus azara*	Annonaceae	*Guatteria punctata*
Stevenson 2015	Stevenson 2015	Colombia	Amazonian	Aves	Piciformes	Ramphastidae	*Pteroglossus pluricinctus*	Annonaceae	*Guatteria punctata*
Stevenson 2015	Stevenson 2015	Colombia	Amazonian	Aves	Passeriformes	Cotingidae	*Querula purpurata*	Annonaceae	*Guatteria punctata*
Stevenson	Stevenson	Colombia	Amazonian	Aves	Piciformes	Ramphastidae	*Ramphastos tucanus*	Annonaceae	*Guatteria*

2015	2015								*punctata*
Stevenson 2015	Stevenson 2015	Colombia	Amazonian	Aves	Piciformes	Ramphastidae	*Ramphastos tucanus*	Annonaceae	*Oxandra mediocris*
Stevenson 2015	Stevenson 2015	Colombia	Amazonian	Mammalia	Primates	Cebidae	*Saimiri sciureus*	Annonaceae	*Malmea dielsiana*
Sudhakaran 2012	Sudhakaran 2012	India	Oriental	Mammalia	Chiroptera	Pteropodidae	*Cynopterus sphinx*	Annonaceae	*Polyalthia longifolia*
Sudhakaran 2012	Sudhakaran 2012	India	Oriental	Mammalia	Chiroptera	Pteropodidae	*Cynopterus sphinx*	Annonaceae	*Polyalthia lateriflora*
Sudhakaran 2012	Sudhakaran 2012	India	Oriental	Mammalia	Chiroptera	Pteropodidae	*Rousettus leschenaultii*	Annonaceae	*Polyalthia longifolia*
Sudhakaran 2012	Sudhakaran 2012	India	Oriental	Mammalia	Chiroptera	Pteropodidae	*Rousettus leschenaultii*	Annonaceae	*Polyalthia lateriflora*
Sudhakaran 2012	Sudhakaran 2012	India	Oriental	Mammalia	Chiroptera	Pteropodidae	*Pteropus giganteus*	Annonaceae	*Polyalthia longifolia*
Sudhakaran 2012	Sudhakaran 2012	India	Oriental	Mammalia	Chiroptera	Pteropodidae	*Pteropus giganteus*	Annonaceae	*Polyalthia lateriflora*
Suzuki 2007	Suzuki 2007	Thailand	Oriental	Mammalia	Rodentia	Sciuridae	*Callosciurus finlaysonii*	Annonaceae	*Platymitra macrocarpa*
Suzuki 2007	Suzuki 2007	Thailand	Oriental	Mammalia	Rodentia	Sciuridae	*Callosciurus finlaysonii*	Annonaceae	*Uvaria concava*
Suzuki 2007	Suzuki 2007	Thailand	Oriental	Mammalia	Rodentia	Sciuridae	*Menetes berdmorei*	Annonaceae	*Alphonsea elliptica*
Suzuki 2007	Suzuki 2007	Thailand	Oriental	Mammalia	Scandentia	Tupaiidae	*Tupaia belangeri*	Annonaceae	*Alphonsea elliptica*
Suzuki 2007	Suzuki 2007	Thailand	Oriental	Mammalia	Scandentia	Tupaiidae	*Tupaia belangeri*	Annonaceae	*Miliusa cuneata*
Suzuki 2007	Suzuki 2007	Thailand	Oriental	Mammalia	Scandentia	Tupaiidae	*Tupaia belangeri*	Annonaceae	*Platymitra macrocarpa*
Suzuki 2007	Suzuki 2007	Thailand	Oriental	Mammalia	Rodentia	Muridae	*Rattus andamanensis*	Annonaceae	*Alphonsea elliptica*
Suzuki 2007	Suzuki 2007	Thailand	Oriental	Mammalia	Rodentia	Muridae	*Rattus andamanensis*	Annonaceae	*Miliusa cuneata*

Suzuki 2007	Suzuki 2007	Thailand	Oriental	Mammalia	Rodentia	Muridae	*Leopoldamys sabanus*	Annonaceae	*Alphonsea elliptica*
Suzuki 2007	Suzuki 2007	Thailand	Oriental	Mammalia	Rodentia	Muridae	*Leopoldamys sabanus*	Annonaceae	*Miliusa cuneata*
Suzuki 2007	Suzuki 2007	Thailand	Oriental	Mammalia	Rodentia	Muridae	*Leopoldamys sabanus*	Annonaceae	*Platymitra macrocarpa*
Suzuki 2007	Suzuki 2007	Thailand	Oriental	Mammalia	Rodentia	Muridae	*Leopoldamys sabanus*	Annonaceae	*Polyalthia viridis*
Suzuki 2007	Suzuki 2007	Thailand	Oriental	Mammalia	Rodentia	Muridae	*Maxomys surifer*	Annonaceae	*Alphonsea elliptica*
Suzuki 2007	Suzuki 2007	Thailand	Oriental	Mammalia	Rodentia	Muridae	*Maxomys surifer*	Annonaceae	*Miliusa cuneata*
Suzuki 2007	Suzuki 2007	Thailand	Oriental	Mammalia	Rodentia	Muridae	*Maxomys surifer*	Annonaceae	*Platymitra macrocarpa*
Suzuki 2007	Suzuki 2007	Thailand	Oriental	Mammalia	Rodentia	Muridae	*Maxomys surifer*	Annonaceae	*Unona jucunda*
Suzuki 2007	Suzuki 2007	Thailand	Oriental	Mammalia	Rodentia	Muridae	*Maxomys surifer*	Annonaceae	*Polyalthia viridis*
Suzuki 2007	Suzuki 2007	Thailand	Oriental	Mammalia	Rodentia	Muridae	*Maxomys surifer*	Annonaceae	*Uvaria concava*
Ungar 1995	Ungar 1995	Indonesia	Indo-Malayan	Mammalia	Primates	Hylobatidae	*Hylobates lar*	Annonaceae	*Cyathocalyx sumatranus*
Ungar 1995	Ungar 1995	Indonesia	Indo-Malayan	Mammalia	Primates	Cercopithecidae	*Macaca fascicularis*	Annonaceae	*Cananga odorata*
Ungar 1995	Ungar 1995	Indonesia	Indo-Malayan	Mammalia	Primates	Cercopithecidae	*Macaca fascicularis*	Annonaceae	*Cyathocalyx sumatranus*
Ungar 1995	Ungar 1995	Indonesia	Indo-Malayan	Mammalia	Primates	Hominidae	*Pongo pygmaeus*	Annonaceae	*Cananga odorata*
Ungar 1995	Ungar 1995	Indonesia	Indo-Malayan	Mammalia	Primates	Hominidae	*Pongo pygmaeus*	Annonaceae	*Cyathocalyx sumatranus*
Yasuda 2005	Yasuda 2005	Malaysia	Indo-Malayan	Mammalia	Primates	Cercopithecidae	*Macaca nemestrina*	Annonaceae	*Xylopia malayana*
Yasuda	Yasuda 2005	Malaysia	Indo-Malayan	Mammalia	Artiodactyla	Suidae	*Sus scrofa*	Annonaceae	*Xylopia malayana*

2005									

Table S2: Hypothesized trait matchings between frugivory-related plant and animal traits.

**Plant traits**	**Animal traits**	**Functionality**	**Reference for functionality**
Fruit length and width.	Bird beak volume index; bird and mammal body mass.	There is a positive correlation between fruit size and bird gape size and beak volume or mammal body size in endozoochoric dispersal due to physical constraints on the size of fruits that can be swallowed, ingested, and effectively dispersed.	Wheelwright (1985), Jordano (2000), Chen & Moles (2015), Lim *et al.* (2020), McFadden *et al*. (2022), Wölke *et al.* (2023).
Growth form: tree (i.e., woody plant at least 5 meters tall, typically with an unbranched main axis in its lower part); shrub (i.e., woody plant less than 5 meters tall, either lacking a distinct main axis or having branches that persist along the main axis nearly to its base); liana (i.e., woody climber).	Bird hand wing index; mammal body mass.	Plant height and hence growth form positively relate to fruit size and dispersal distance, similar as body mass approximates (home) range size in mammals, and wing shape (hand wing index) approximates flight efficiency and dispersal ability in birds.	Carbone *et al*. (2005), Thomson *et al*. (2011), Pires *et al*. (2018), Claramunt (2021).
	Foraging strata.	Growth form also affects the display of fruits across forest strata, and thus interaction with frugivore guilds that are restricted to particular strata (e.g., understory vs. canopy frugivores).	Givnish (2010), Onstein *et al*. (2017), Thiel *et al*. (2023).

Table S3: Spatial information obtained for Annonaceae species from original publications and from the Global Biodiversity Information Facility (GBIF, 2022). In absence of coordinates, we assigned the coordinate by centroid of the municipality reported in the original publications. This information was added to those obtained from the Taxonomic Databases Working Group (TDWG; https://www.tdwg.org/).

Taxon	Botanical country (code)	Continent	Source
Alphonsea annulata	THA	Asia Tropical	Leeratiwong *et al.*, 2021a
Alphonsea tsangyuanensis	CHC	Asia Temperate	GBIF, 2022
Ambavia gerrardii	MDG	Africa	GBIF, 2022
Anaxagorea radiata	PHI	Asia Tropical	GBIF, 2022
Annona neolaurifolia	BZC	Southern America	GBIF, 2022
Annona neolaurifolia	BZE	Southern America	GBIF, 2022
Annona neolaurifolia	BZL	Southern America	GBIF, 2022
Annona neolaurifolia	BZS	Southern America	GBIF, 2022
Annona primigenia	BLZ	Southern America	GBIF, 2022
Annona primigenia	GUA	Southern America	GBIF, 2022
Annona primigenia	MXS	Northern America	GBIF, 2022
Annona primigenia	MXT	Northern America	GBIF, 2022
Annona ubatubensis	BZL	Southern America	GBIF, 2022
Anonidium brieyi	GAB	Africa	GBIF, 2022
Artabotrys stolzii	BUR	Africa	GBIF, 2022
Artabotrys stolzii	MLW	Africa	GBIF, 2022
Artabotrys stolzii	TAN	Africa	GBIF, 2022
Artabotrys stolzii	ZAM	Africa	GBIF, 2022
Asimina longifolia	ALA	Northern America	GBIF, 2022
Asimina longifolia	FLA	Northern America	GBIF, 2022
Asimina longifolia	GEO	Northern America	GBIF, 2022
Asimina pulchella	FLA	Northern America	GBIF, 2022
Asimina rugelii	FLA	Northern America	Kral 1960
Asimina speciosa	FLA	Northern America	GBIF, 2022
Asimina speciosa	GEO	Northern America	GBIF, 2022
Bocagea asymmetrica	BZL	Southern America	GBIF, 2022
Bocagea moeniana	BZL	Southern America	Mello-Silva & Lopes, 2020
Desmos dubius	THA	Asia Tropical	GBIF, 2022
Disepalum platipetalum	SUM	Asia Tropical	GBIF, 2022
Fissistigma bracteolatum	CHC	Asia Temperate	GBIF, 2022
Fissistigma bracteolatum	VIE	Asia Tropical	GBIF, 2022
Goniothalamus parallelivenius	BOR	Asia Tropical	GBIF, 2022
Goniothalamus roseipetalus	THA	Asia Tropical	Leeratiwong *et al.*, 2021b
Goniothalamus sukhirinensis	THA	Asia Tropical	Leeratiwong et al., 2021b
Guatteria costaricencis	COS	Southern America	GBIF, 2022
Guatteria costaricencis	MXT	Northern America	GBIF, 2022
Guatteria sanctae-crucis	BOL	Southern America	Maas *et al.*, 2015
Guatteria sanctae-crucis	PER	Southern America	Maas *et al.*, 2015

Hornschuchia mediterranea	BZE	Southern America	Mello-Silva *et al.*, 2021
Meiogyne hainanensis	CHH	Asia Temperate	GBIF, 2022
Meiogyne hainanensis	THA	Asia Tropical	GBIF, 2022
Meiogyne oligocarpa	CHC	Asia Temperate	Xue *et al.*, 2021a
Meiogyne tiebaghiensis	NWC	Pacific	GBIF, 2022
Melodorum fruticosum	CBD	Asia Tropical	GBIF, 2022
Melodorum fruticosum	JAW	Asia Tropical	GBIF, 2022
Melodorum fruticosum	THA	Asia Tropical	GBIF, 2022
Melodorum leichhardtii	NSW	Australasia	GBIF, 2022
Melodorum leichhardtii	NWG	Asia Tropical	GBIF, 2022
Melodorum leichhardtii	QLD	Australasia	GBIF, 2022
Miliusa longipes	MLY	Asia Tropical	GBIF, 2022
Miliusa longipes	SUM	Asia Tropical	GBIF, 2022
Miliusa longipes	THA	Asia Tropical	GBIF, 2022
Miliusa sinensis	CHC	Asia Temperate	GBIF, 2022
Miliusa sinensis	VIE	Asia Tropical	GBIF, 2022
Monanthotaxis suffruticosa	MOZ	Africa	Hoekstra *et al.*, 2021
Monanthotaxis suffruticosa	TAN	Africa	Hoekstra *et al.*, 2021
Mosannona garwoodii	PAN	Southern America	GBIF, 2022
Neostenanthera platypetala	CMN	Africa	GBIF, 2022
Orophea sichaikhanii	THA	Asia Tropical	Damthongdee *et al.*, 2021
Phaeanthus piyae	THA	Asia Tropical	Wiya *et al.*, 2021
Piptostigma mortehani	CAF	Africa	GBIF, 2022
Piptostigma mortehani	GAB	Africa	GBIF, 2022
Piptostigma mortehani	ZAI	Africa	GBIF, 2022
Polyalthia longipes	BOR	Asia Tropical	GBIF, 2022
Polyalthia longipes	JAW	Asia Tropical	GBIF, 2022
Polyceratocarpus askhambryan-iringae	TAN	Africa	Marshall *et al.*, 2016
Pseudoxandra spiritus-sancti	BZL	Southern America	Maas & Westra, 2003
Pseuduvaria khaosokensis	THA	Asia Tropical	Yoosukkee *et al.*, 2020
Pseuduvaria luzonensis	PHI	Asia Tropical	Su & Saunders, 2006
Pseuduvaria nova-guineensis	NWG	Asia Tropical	Su & Saunders, 2006
Schefferomitra subaequalis	NWG	Asia Tropical	GBIF, 2022
Tetrameranthus globuliferus	ECU	Southern America	GBIF, 2022
Trivalvaria tomentosa	CHC	Asia Temperate	Xue *et al.*, 2021b
Unonopsis renati	BZL	Southern America	GBIF, 2022
Unonopsis sanctae-teresae	BZL	Southern America	Maas *et al.*, 2007
Uvaria anonoides	IVO	Africa	GBIF, 2022
Uvaria anonoides	ZAI	Africa	GBIF, 2022
Uvaria cherrevensis	THA	Asia Tropical	GBIF, 2022
Uvaria holtzei	NTA	Australasia	GBIF, 2022
Uvaria holtzei	QLD	Australasia	GBIF, 2022
Uvaria javana	BOR	Asia Tropical	GBIF, 2022
Uvaria javana	MLY	Asia Tropical	GBIF, 2022
Uvariodendron dzomboense	KEN	Africa	GBIF, 2022
Uvariodendron dzomboense	TAN	Africa	GBIF, 2022
Uvariodendron gorgone	MOZ	Africa	Dagallier, 2021
Uvariodendron gorgone	TAN	Africa	Dagallier, 2021
Uvariodendron mbagoi	TAN	Africa	GBIF, 2022
Uvariodendron occidentalis	IVO	Africa	GBIF, 2022
Uvariodendron schmidtii	KEN	Africa	GBIF, 2022

Uvariopsis pedunculosa	CMN	Africa	Dagallier, 2021
Uvariopsis pedunculosa	GAB	Africa	Dagallier, 2021
Uvariopsis pedunculosa	ZAI	Africa	Dagallier, 2021
Winitia cauliflora	THA	Asia Tropical	GBIF, 2022
Xylopia anomala	MDG	Africa	GBIF, 2022
Xylopia australis	MDG	Africa	GBIF, 2022
Xylopia carinata	BZC	Southern America	GBIF, 2022
Xylopia carinata	MDG	Africa	GBIF, 2022
Xylopia galokothamna	MDG	Africa	GBIF, 2022
Xylopia lokobensis	MDG	Africa	GBIF, 2022
Xylopia longirostra	MDG	Africa	GBIF, 2022
Xylopia marojejyana	MDG	Africa	GBIF, 2022
Xylopia perrierii	MDG	Africa	GBIF, 2022
Xylopia ravelonarivoi	MDG	Africa	GBIF, 2022
Xylopia retusa	MDG	Africa	GBIF, 2022
Xylopia sclerophylla	MDG	Africa	GBIF, 2022
Xylopia stenopetala	BOR	Asia Tropical	GBIF, 2022
Xylopia stenopetala	SUM	Asia Tropical	GBIF, 2022

Table S4: Number of animal species included in the analyses by family. Our analyses focused on terrestrial birds and mammals that co-occur in botanical countries with at least four Annonaceae species. Based on the percentage of fruits in the diet, we classified animals into two functional groups: 'frugivores' as animals with at least 50% of fruits in their diet, and 'non-frugivores' as animals with 0% fruits in their diet. The final dataset included 948 frugivorous birds, 3,979 non-frugivorous birds, 470 frugivorous mammals, and 2,210 non-frugivorous mammals.

Class	Order	Family	Functional group	Number of species
Aves	Accipitriformes	Accipitridae	Frugivores	1
Aves	Passeriformes	Aegithalidae	Frugivores	1
Aves	Passeriformes	Bombycillidae	Frugivores	3
Aves	Bucerotiformes	Bucerotidae	Frugivores	26
Aves	Psittaciformes	Cacatuidae	Frugivores	5
Aves	Passeriformes	Calyptomenidae	Frugivores	1
Aves	Passeriformes	Campephagidae	Frugivores	8
Aves	Piciformes	Capitonidae	Frugivores	11
Aves	Passeriformes	Cardinalidae	Frugivores	5
Aves	Struthioniformes	Casuariidae	Frugivores	2
Aves	Passeriformes	Chloropseidae	Frugivores	4
Aves	Coliiformes	Coliidae	Frugivores	6
Aves	Columbiformes	Columbidae	Frugivores	132
Aves	Passeriformes	Corvidae	Frugivores	17
Aves	Passeriformes	Cotingidae	Frugivores	56
Aves	Galliformes	Cracidae	Frugivores	39
Aves	Cuculiformes	Cuculidae	Frugivores	6
Aves	Passeriformes	Dicaeidae	Frugivores	18
Aves	Passeriformes	Dulidae	Frugivores	1
Aves	Passeriformes	Eurylaimidae	Frugivores	1
Aves	Passeriformes	Fringillidae	Frugivores	33
Aves	Passeriformes	Hypocoliidae	Frugivores	1
Aves	Passeriformes	Icteridae	Frugivores	10
Aves	Passeriformes	Irenidae	Frugivores	2
Aves	Passeriformes	Leiotrichidae	Frugivores	1
Aves	Piciformes	Lybiidae	Frugivores	25
Aves	Piciformes	Megalaimidae	Frugivores	1
Aves	Galliformes	Megapodiidae	Frugivores	1
Aves	Passeriformes	Melanocharitidae	Frugivores	1
Aves	Passeriformes	Meliphagidae	Frugivores	2
Aves	Passeriformes	Mimidae	Frugivores	3
Aves	Passeriformes	Mitrospingidae	Frugivores	1
Aves	Passeriformes	Modulatricidae	Frugivores	1
Aves	Coraciiformes	Momotidae	Frugivores	1
Aves	Passeriformes	Muscicapidae	Frugivores	4

Aves	Musophagiformes	Musophagidae	Frugivores	17
Aves	Galliformes	Odontophoridae	Frugivores	2
Aves	Passeriformes	Oriolidae	Frugivores	22
Aves	Passeriformes	Paradisaeidae	Frugivores	13
Aves	Passeriformes	Passerellidae	Frugivores	6
Aves	Galliformes	Phasianidae	Frugivores	5
Aves	Passeriformes	Philepittidae	Frugivores	1
Aves	Piciformes	Picidae	Frugivores	6
Aves	Passeriformes	Pipridae	Frugivores	38
Aves	Passeriformes	Ploceidae	Frugivores	1
Aves	Psittaciformes	Psittacidae	Frugivores	95
Aves	Gruiformes	Psophiidae	Frugivores	3
Aves	Passeriformes	Ptiliogonatidae	Frugivores	2
Aves	Passeriformes	Ptilonorhynchidae	Frugivores	8
Aves	Passeriformes	Pycnonotidae	Frugivores	34
Aves	Piciformes	Ramphastidae	Frugivores	29
Aves	Passeriformes	Sapayoidae	Frugivores	1
Aves	Piciformes	Semnornithidae	Frugivores	2
Aves	Passeriformes	Spindalidae	Frugivores	4
Aves	Caprimulgiformes	Steatornithidae	Frugivores	1
Aves	Passeriformes	Sturnidae	Frugivores	38
Aves	Passeriformes	Thraupidae	Frugivores	63
Aves	Struthioniformes	Tinamidae	Frugivores	6
Aves	Passeriformes	Tityridae	Frugivores	18
Aves	Trogoniformes	Trogonidae	Frugivores	20
Aves	Passeriformes	Turdidae	Frugivores	36
Aves	Passeriformes	Tyrannidae	Frugivores	36
Aves	Passeriformes	Vireonidae	Frugivores	4
Aves	Passeriformes	Zosteropidae	Frugivores	7
Mammalia	Rodentia	Anomaluridae	Frugivores	2
Mammalia	Primates	Atelidae	Frugivores	7
Mammalia	Cetartiodactyla	Bovidae	Frugivores	1
Mammalia	Primates	Callitrichidae	Frugivores	5
Mammalia	Primates	Cebidae	Frugivores	1
Mammalia	Primates	Cercopithecidae	Frugivores	31
Mammalia	Cetartiodactyla	Cervidae	Frugivores	1
Mammalia	Primates	Cheirogaleidae	Frugivores	3
Mammalia	Rodentia	Cricetidae	Frugivores	18
Mammalia	Didelphimorphia	Didelphidae	Frugivores	14
Mammalia	Rodentia	Echimyidae	Frugivores	22
Mammalia	Primates	Hominidae	Frugivores	4
Mammalia	Primates	Hylobatidae	Frugivores	13
Mammalia	Primates	Lemuridae	Frugivores	6
Mammalia	Primates	Lorisidae	Frugivores	1
Mammalia	Diprotodontia	Macropodidae	Frugivores	15
Mammalia	Rodentia	Muridae	Frugivores	58

Mammalia	Carnivora	Nandiniidae	Frugivores	1
Mammalia	Rodentia	Nesomyidae	Frugivores	7
Mammalia	Rodentia	Octodontidae	Frugivores	1
Mammalia	Diprotodontia	Phalangeridae	Frugivores	2
Mammalia	Chiroptera	Phyllostomidae	Frugivores	68
Mammalia	Primates	Pitheciidae	Frugivores	7
Mammalia	Carnivora	Procyonidae	Frugivores	8
Mammalia	Chiroptera	Pteropodidae	Frugivores	146
Mammalia	Perissodactyla	Rhinocerotidae	Frugivores	1
Mammalia	Rodentia	Sciuridae	Frugivores	25
Mammalia	Carnivora	Ursidae	Frugivores	2
Aves	Passeriformes	Acanthizidae	Non-frugivores	37
Aves	Accipitriformes	Accipitridae	Non-frugivores	190
Aves	Passeriformes	Acrocephalidae	Non-frugivores	18
Aves	Passeriformes	Aegithalidae	Non-frugivores	3
Aves	Passeriformes	Aegithinidae	Non-frugivores	4
Aves	Caprimulgiformes	Aegothelidae	Non-frugivores	7
Aves	Passeriformes	Alaudidae	Non-frugivores	56
Aves	Coraciiformes	Alcedinidae	Non-frugivores	58
Aves	Anseriformes	Anatidae	Non-frugivores	77
Aves	Anseriformes	Anhimidae	Non-frugivores	3
Aves	Suliformes	Anhingidae	Non-frugivores	4
Aves	Anseriformes	Anseranatidae	Non-frugivores	1
Aves	Caprimulgiformes	Apodidae	Non-frugivores	69
Aves	Gruiformes	Aramidae	Non-frugivores	1
Aves	Pelecaniformes	Ardeidae	Non-frugivores	52
Aves	Passeriformes	Artamidae	Non-frugivores	10
Aves	Passeriformes	Atrichornithidae	Non-frugivores	2
Aves	Pelecaniformes	Balaenicipitidae	Non-frugivores	1
Aves	Passeriformes	Bernieridae	Non-frugivores	5
Aves	Coraciiformes	Brachypteraciidae	Non-frugivores	3
Aves	Piciformes	Bucconidae	Non-frugivores	27
Aves	Passeriformes	Buphagidae	Non-frugivores	1
Aves	Charadriiformes	Burhinidae	Non-frugivores	7
Aves	Psittaciformes	Cacatuidae	Non-frugivores	3
Aves	Passeriformes	Calcariidae	Non-frugivores	4
Aves	Passeriformes	Calyptomenidae	Non-frugivores	3
Aves	Passeriformes	Calyptophilidae	Non-frugivores	2
Aves	Passeriformes	Campephagidae	Non-frugivores	24
Aves	Caprimulgiformes	Caprimulgidae	Non-frugivores	54
Aves	Passeriformes	Cardinalidae	Non-frugivores	11
Aves	Cariamiformes	Cariamidae	Non-frugivores	1
Aves	Cathartiformes	Cathartidae	Non-frugivores	6
Aves	Passeriformes	Certhiidae	Non-frugivores	8
Aves	Passeriformes	Chaetopidae	Non-frugivores	2
Aves	Charadriiformes	Charadriidae	Non-frugivores	48

Aves	Ciconiiformes	Ciconiidae	Non-frugivores	18
Aves	Passeriformes	Cinclosomatidae	Non-frugivores	7
Aves	Passeriformes	Cisticolidae	Non-frugivores	112
Aves	Passeriformes	Climacteridae	Non-frugivores	6
Aves	Columbiformes	Columbidae	Non-frugivores	46
Aves	Passeriformes	Conopophagidae	Non-frugivores	11
Aves	Coraciiformes	Coraciidae	Non-frugivores	10
Aves	Passeriformes	Corcoracidae	Non-frugivores	2
Aves	Passeriformes	Corvidae	Non-frugivores	15
Aves	Cuculiformes	Cuculidae	Non-frugivores	78
Aves	Passeriformes	Dasyornithidae	Non-frugivores	2
Aves	Passeriformes	Dicruridae	Non-frugivores	18
Aves	Passeriformes	Donacobiidae	Non-frugivores	1
Aves	Charadriiformes	Dromadidae	Non-frugivores	1
Aves	Passeriformes	Emberizidae	Non-frugivores	32
Aves	Passeriformes	Estrildidae	Non-frugivores	77
Aves	Passeriformes	Eupetidae	Non-frugivores	1
Aves	Passeriformes	Eurylaimidae	Non-frugivores	4
Aves	Eurypygiformes	Eurypygidae	Non-frugivores	1
Aves	Falconiformes	Falconidae	Non-frugivores	52
Aves	Passeriformes	Formicariidae	Non-frugivores	9
Aves	Passeriformes	Fringillidae	Non-frugivores	13
Aves	Passeriformes	Furnariidae	Non-frugivores	221
Aves	Piciformes	Galbulidae	Non-frugivores	18
Aves	Gaviiformes	Gaviidae	Non-frugivores	4
Aves	Charadriiformes	Glareolidae	Non-frugivores	13
Aves	Passeriformes	Grallariidae	Non-frugivores	33
Aves	Gruiformes	Gruidae	Non-frugivores	3
Aves	Charadriiformes	Haematopodidae	Non-frugivores	6
Aves	Gruiformes	Heliornithidae	Non-frugivores	3
Aves	Caprimulgiformes	Hemiprocnidae	Non-frugivores	4
Aves	Passeriformes	Hirundinidae	Non-frugivores	45
Aves	Passeriformes	Hyliotidae	Non-frugivores	3
Aves	Passeriformes	Icteridae	Non-frugivores	29
Aves	Piciformes	Indicatoridae	Non-frugivores	5
Aves	Charadriiformes	Jacanidae	Non-frugivores	8
Aves	Passeriformes	Lamproliidae	Non-frugivores	2
Aves	Passeriformes	Laniidae	Non-frugivores	20
Aves	Charadriiformes	Laridae	Non-frugivores	27
Aves	Passeriformes	Leiotrichidae	Non-frugivores	12
Aves	Leptosomiformes	Leptosomidae	Non-frugivores	1
Aves	Passeriformes	Locustellidae	Non-frugivores	18
Aves	Passeriformes	Machaerirhynchidae	Non-frugivores	1
Aves	Passeriformes	Macrosphenidae	Non-frugivores	14
Aves	Passeriformes	Malaconotidae	Non-frugivores	21
Aves	Passeriformes	Maluridae	Non-frugivores	17

Aves	Galliformes	Megapodiidae	Non-frugivores	8
Aves	Passeriformes	Melanocharitidae	Non-frugivores	2
Aves	Passeriformes	Melanopareiidae	Non-frugivores	4
Aves	Passeriformes	Meliphagidae	Non-frugivores	48
Aves	Passeriformes	Menuridae	Non-frugivores	1
Aves	Coraciiformes	Meropidae	Non-frugivores	26
Aves	Mesitornithiformes	Mesitornithidae	Non-frugivores	2
Aves	Passeriformes	Mimidae	Non-frugivores	4
Aves	Passeriformes	Modulatricidae	Non-frugivores	1
Aves	Coraciiformes	Momotidae	Non-frugivores	5
Aves	Passeriformes	Monarchidae	Non-frugivores	41
Aves	Passeriformes	Motacillidae	Non-frugivores	40
Aves	Passeriformes	Muscicapidae	Non-frugivores	90
Aves	Passeriformes	Nectariniidae	Non-frugivores	24
Aves	Passeriformes	Neosittidae	Non-frugivores	1
Aves	Passeriformes	Nicatoridae	Non-frugivores	2
Aves	Caprimulgiformes	Nyctibiidae	Non-frugivores	6
Aves	Galliformes	Odontophoridae	Non-frugivores	9
Aves	Passeriformes	Oreoicidae	Non-frugivores	1
Aves	Passeriformes	Orthonychidae	Non-frugivores	2
Aves	Otidiformes	Otididae	Non-frugivores	4
Aves	Passeriformes	Pachycephalidae	Non-frugivores	19
Aves	Passeriformes	Panuridae	Non-frugivores	1
Aves	Passeriformes	Pardalotidae	Non-frugivores	3
Aves	Passeriformes	Paridae	Non-frugivores	7
Aves	Passeriformes	Parulidae	Non-frugivores	26
Aves	Passeriformes	Passerellidae	Non-frugivores	21
Aves	Passeriformes	Passeridae	Non-frugivores	17
Aves	Charadriiformes	Pedionomidae	Non-frugivores	1
Aves	Pelecaniformes	Pelecanidae	Non-frugivores	6
Aves	Passeriformes	Pellorneidae	Non-frugivores	25
Aves	Passeriformes	Petroicidae	Non-frugivores	18
Aves	Passeriformes	Peucedramidae	Non-frugivores	1
Aves	Passeriformes	Phaenicophilidae	Non-frugivores	2
Aves	Suliformes	Phalacrocoracidae	Non-frugivores	4
Aves	Galliformes	Phasianidae	Non-frugivores	26
Aves	Passeriformes	Philepittidae	Non-frugivores	1
Aves	Phoenicopteriformes	Phoenicopteridae	Non-frugivores	5
Aves	Bucerotiformes	Phoeniculidae	Non-frugivores	3
Aves	Passeriformes	Phylloscopidae	Non-frugivores	41
Aves	Passeriformes	Picathartidae	Non-frugivores	2
Aves	Piciformes	Picidae	Non-frugivores	107
Aves	Passeriformes	Pittidae	Non-frugivores	14
Aves	Passeriformes	Platylophidae	Non-frugivores	1
Aves	Passeriformes	Platysteiridae	Non-frugivores	22
Aves	Passeriformes	Ploceidae	Non-frugivores	49

Aves	Charadriiformes	Pluvianellidae	Non-frugivores	1
Aves	Charadriiformes	Pluvianidae	Non-frugivores	1
Aves	Passeriformes	Pnoepygidae	Non-frugivores	4
Aves	Caprimulgiformes	Podargidae	Non-frugivores	11
Aves	Podicipediformes	Podicipedidae	Non-frugivores	14
Aves	Passeriformes	Polioptilidae	Non-frugivores	7
Aves	Passeriformes	Pomatostomidae	Non-frugivores	2
Aves	Passeriformes	Promeropidae	Non-frugivores	2
Aves	Passeriformes	Prunellidae	Non-frugivores	3
Aves	Psittaciformes	Psittacidae	Non-frugivores	34
Aves	Passeriformes	Psophodidae	Non-frugivores	3
Aves	Pterocliformes	Pteroclidae	Non-frugivores	14
Aves	Passeriformes	Pycnonotidae	Non-frugivores	9
Aves	Gruiformes	Rallidae	Non-frugivores	69
Aves	Piciformes	Ramphastidae	Non-frugivores	1
Aves	Charadriiformes	Recurvirostridae	Non-frugivores	6
Aves	Passeriformes	Regulidae	Non-frugivores	2
Aves	Passeriformes	Remizidae	Non-frugivores	4
Aves	Passeriformes	Rhinocryptidae	Non-frugivores	29
Aves	Passeriformes	Rhipiduridae	Non-frugivores	28
Aves	Charadriiformes	Rostratulidae	Non-frugivores	2
Aves	Accipitriformes	Sagittariidae	Non-frugivores	1
Aves	Charadriiformes	Scolopacidae	Non-frugivores	61
Aves	Pelecaniformes	Scopidae	Non-frugivores	1
Aves	Passeriformes	Scotocercidae	Non-frugivores	16
Aves	Passeriformes	Sittidae	Non-frugivores	15
Aves	Passeriformes	Stenostiridae	Non-frugivores	8
Aves	Strigiformes	Strigidae	Non-frugivores	146
Aves	Passeriformes	Sturnidae	Non-frugivores	2
Aves	Passeriformes	Sylviidae	Non-frugivores	4
Aves	Passeriformes	Teretistridae	Non-frugivores	1
Aves	Passeriformes	Thamnophilidae	Non-frugivores	147
Aves	Charadriiformes	Thinocoridae	Non-frugivores	3
Aves	Passeriformes	Thraupidae	Non-frugivores	64
Aves	Pelecaniformes	Threskiornithidae	Non-frugivores	30
Aves	Passeriformes	Timaliidae	Non-frugivores	13
Aves	Struthioniformes	Tinamidae	Non-frugivores	12
Aves	Passeriformes	Tityridae	Non-frugivores	12
Aves	Coraciiformes	Todidae	Non-frugivores	3
Aves	Caprimulgiformes	Trochilidae	Non-frugivores	262
Aves	Passeriformes	Troglodytidae	Non-frugivores	32
Aves	Trogoniformes	Trogonidae	Non-frugivores	8
Aves	Passeriformes	Turdidae	Non-frugivores	3
Aves	Charadriiformes	Turnicidae	Non-frugivores	15
Aves	Passeriformes	Tyrannidae	Non-frugivores	216
Aves	Strigiformes	Tytonidae	Non-frugivores	10

Aves	Bucerotiformes	Upupidae	Non-frugivores	1
Aves	Passeriformes	Urocynchramidae	Non-frugivores	1
Aves	Passeriformes	Vangidae	Non-frugivores	26
Aves	Passeriformes	Viduidae	Non-frugivores	10
Aves	Passeriformes	Vireonidae	Non-frugivores	16
Aves	Passeriformes	Zeledoniidae	Non-frugivores	1
Aves	Passeriformes	Zosteropidae	Non-frugivores	4
Mammalia	Rodentia	Abrocomidae	Non-frugivores	10
Mammalia	Diprotodontia	Acrobatidae	Non-frugivores	1
Mammalia	Rodentia	Anomaluridae	Non-frugivores	1
Mammalia	Cetartiodactyla	Antilocapridae	Non-frugivores	1
Mammalia	Rodentia	Bathyergidae	Non-frugivores	6
Mammalia	Cetartiodactyla	Bovidae	Non-frugivores	63
Mammalia	Pilosa	Bradypodidae	Non-frugivores	3
Mammalia	Cetartiodactyla	Camelidae	Non-frugivores	1
Mammalia	Carnivora	Canidae	Non-frugivores	18
Mammalia	Rodentia	Caviidae	Non-frugivores	10
Mammalia	Primates	Cercopithecidae	Non-frugivores	3
Mammalia	Cetartiodactyla	Cervidae	Non-frugivores	20
Mammalia	Primates	Cheirogaleidae	Non-frugivores	4
Mammalia	Rodentia	Chinchillidae	Non-frugivores	3
Mammalia	Cingulata	Chlamyphoridae	Non-frugivores	11
Mammalia	Afrosoricida	Chrysochloridae	Non-frugivores	19
Mammalia	Chiroptera	Cistugidae	Non-frugivores	2
Mammalia	Chiroptera	Craseonycteridae	Non-frugivores	1
Mammalia	Rodentia	Cricetidae	Non-frugivores	229
Mammalia	Rodentia	Ctenodactylidae	Non-frugivores	3
Mammalia	Rodentia	Ctenomyidae	Non-frugivores	49
Mammalia	Pilosa	Cyclopedidae	Non-frugivores	1
Mammalia	Dermoptera	Cynocephalidae	Non-frugivores	1
Mammalia	Cingulata	Dasypodidae	Non-frugivores	7
Mammalia	Dasyuromorphia	Dasyuridae	Non-frugivores	52
Mammalia	Primates	Daubentoniidae	Non-frugivores	1
Mammalia	Didelphimorphia	Didelphidae	Non-frugivores	10
Mammalia	Rodentia	Dipodidae	Non-frugivores	2
Mammalia	Rodentia	Echimyidae	Non-frugivores	6
Mammalia	Proboscidea	Elephantidae	Non-frugivores	2
Mammalia	Chiroptera	Emballonuridae	Non-frugivores	40
Mammalia	Perissodactyla	Equidae	Non-frugivores	5
Mammalia	Eulipotyphla	Erinaceidae	Non-frugivores	9
Mammalia	Carnivora	Eupleridae	Non-frugivores	5
Mammalia	Carnivora	Felidae	Non-frugivores	24
Mammalia	Chiroptera	Furipteridae	Non-frugivores	2
Mammalia	Primates	Galagidae	Non-frugivores	4
Mammalia	Rodentia	Geomyidae	Non-frugivores	24
Mammalia	Cetartiodactyla	Giraffidae	Non-frugivores	2

Mammalia	Carnivora	Herpestidae	Non-frugivores	11
Mammalia	odentia	Heteromyidae	Non-frugivores	30
Mammalia	Chiroptera	Hipposideridae	Non-frugivores	73
Mammalia	Carnivora	Hyaenidae	Non-frugivores	2
Mammalia	Primates	Indriidae	Non-frugivores	2
Mammalia	Primates	Lemuridae	Non-frugivores	4
Mammalia	Primates	Lepilemuridae	Non-frugivores	8
Mammalia	Lagomorpha	Leporidae	Non-frugivores	45
Mammalia	Primates	Lorisidae	Non-frugivores	1
Mammalia	Diprotodontia	Macropodidae	Non-frugivores	36
Mammalia	Macroscelidea	Macroscelididae	Non-frugivores	12
Mammalia	Pholidota	Manidae	Non-frugivores	4
Mammalia	Chiroptera	Megadermatidae	Non-frugivores	4
Mammalia	Carnivora	Mephitidae	Non-frugivores	5
Mammalia	Chiroptera	Miniopteridae	Non-frugivores	17
Mammalia	Chiroptera	Molossidae	Non-frugivores	90
Mammalia	Chiroptera	Mormoopidae	Non-frugivores	8
Mammalia	Cetartiodactyla	Moschidae	Non-frugivores	6
Mammalia	Rodentia	Muridae	Non-frugivores	270
Mammalia	Carnivora	Mustelidae	Non-frugivores	21
Mammalia	Rodentia	Myocastoridae	Non-frugivores	1
Mammalia	Dasyuromorphia	Myrmecobiidae	Non-frugivores	1
Mammalia	Pilosa	Myrmecophagidae	Non-frugivores	3
Mammalia	Chiroptera	Myzopodidae	Non-frugivores	1
Mammalia	Chiroptera	Natalidae	Non-frugivores	8
Mammalia	Rodentia	Nesomyidae	Non-frugivores	15
Mammalia	Chiroptera	Noctilionidae	Non-frugivores	2
Mammalia	Notoryctemorphia	Notoryctidae	Non-frugivores	2
Mammalia	Chiroptera	Nycteridae	Non-frugivores	16
Mammalia	Lagomorpha	Ochotonidae	Non-frugivores	13
Mammalia	Rodentia	Octodontidae	Non-frugivores	2
Mammalia	Tubulidentata	Orycteropodidae	Non-frugivores	1
Mammalia	Rodentia	Pedetidae	Non-frugivores	2
Mammalia	Peramelemorphia	Peramelidae	Non-frugivores	9
Mammalia	Diprotodontia	Petauridae	Non-frugivores	6
Mammalia	Diprotodontia	Phalangeridae	Non-frugivores	3
Mammalia	Diprotodontia	Phascolarctidae	Non-frugivores	1
Mammalia	Chiroptera	Phyllostomidae	Non-frugivores	11
Mammalia	Diprotodontia	Potoroidae	Non-frugivores	6
Mammalia	Carnivora	Prionodontidae	Non-frugivores	2
Mammalia	Hyracoidea	Procaviidae	Non-frugivores	2
Mammalia	Diprotodontia	Pseudocheiridae	Non-frugivores	2
Mammalia	Chiroptera	Pteropodidae	Non-frugivores	5
Mammalia	Perissodactyla	Rhinocerotidae	Non-frugivores	2
Mammalia	Chiroptera	Rhinolophidae	Non-frugivores	71
Mammalia	Chiroptera	Rhinopomatidae	Non-frugivores	3

Mammalia	Rodentia	Sciuridae	Non-frugivores	28
Mammalia	Eulipotyphla	Soricidae	Non-frugivores	288
Mammalia	Rodentia	Spalacidae	Non-frugivores	7
Mammalia	Cetartiodactyla	Suidae	Non-frugivores	8
Mammalia	Monotremata	Tachyglossidae	Non-frugivores	3
Mammalia	Eulipotyphla	Talpidae	Non-frugivores	18
Mammalia	Primates	Tarsiidae	Non-frugivores	4
Mammalia	Diprotodontia	Tarsipedidae	Non-frugivores	1
Mammalia	Cetartiodactyla	Tayassuidae	Non-frugivores	1
Mammalia	Afrosoricida	Tenrecidae	Non-frugivores	25
Mammalia	Peramelemorphia	Thylacomyidae	Non-frugivores	1
Mammalia	Chiroptera	Thyropteridae	Non-frugivores	3
Mammalia	Scandentia	Tupaiidae	Non-frugivores	16
Mammalia	Carnivora	Ursidae	Non-frugivores	1
Mammalia	Chiroptera	Vespertilionidae	Non-frugivores	294
Mammalia	Carnivora	Viverridae	Non-frugivores	15
Mammalia	Diprotodontia	Vombatidae	Non-frugivores	3

Table S5: Number of bird and mammal species not included in our analyses due to a less strictly fruit-based diet (a total of 2,353 bird species and 1,613 mammal species). These species consume less than 50% fruit in their overall diet and are represented here in four frugivory categories: 10%, 20%, 30%, or 40% of fruits in their diet (as reported in EltonTraits; Wilman *et al.*, 2014).

**Class**	**Order**	**Family**	**Number of species per frugivory category**			
			**10%**	**20%**	**30%**	**40%**
Aves	Accipitriformes	Accipitridae	3	1	0	0
Aves	Anseriformes	Anatidae	11	5	0	2
Aves	Apterygiformes	Apterygidae	3	0	0	0
Aves	Bucerotiformes	Bucerotidae	7	4	0	5
Aves	Bucerotiformes	Bucorvidae	1	0	0	0
Aves	Bucerotiformes	Phoeniculidae	2	3	0	0
Aves	Cariamiformes	Cariamidae	1	0	0	0
Aves	Casuariiformes	Dromaiidae	0	0	1	0
Aves	Charadriiformes	Charadriidae	5	0	0	0
Aves	Charadriiformes	Laridae	4	3	0	0
Aves	Charadriiformes	Scolopacidae	7	2	2	1
Aves	Charadriiformes	Stercorariidae	1	1	0	0
Aves	Charadriiformes	Turnicidae	0	0	1	0
Aves	Columbiformes	Columbidae	4	17	24	20
Aves	Coraciiformes	Coraciidae	1	0	0	0
Aves	Coraciiformes	Momotidae	1	1	2	0
Aves	Coraciiformes	Todidae	2	0	0	0
Aves	Cuculiformes	Cuculidae	18	13	1	0
Aves	Falconiformes	Falconidae	2	0	0	0
Aves	Galliformes	Cracidae	0	0	3	2
Aves	Galliformes	Megapodiidae	5	1	3	2
Aves	Galliformes	Numididae	4	1	1	0
Aves	Galliformes	Odontophoridae	2	2	12	1
Aves	Galliformes	Phasianidae	19	31	34	16
Aves	Gruiformes	Gruidae	3	1	0	0
Aves	Gruiformes	Rallidae	12	5	1	0
Aves	Mesitornithiformes	Mesitornithidae	1	0	0	0
Aves	Musophagiformes	Musophagidae	0	0	0	1
Aves	Otidiformes	Otididae	2	11	1	0
Aves	Passeriformes	Acanthisittidae	2	0	0	0
Aves	Passeriformes	Acanthizidae	7	3	0	0
Aves	Passeriformes	Aegithalidae	3	1	0	0
Aves	Passeriformes	Alaudidae	5	2	0	0
Aves	Passeriformes	Artamidae	1	0	0	0
Aves	Passeriformes	Callaeatidae	0	0	0	1
Aves	Passeriformes	Campephagidae	7	20	3	0

Aves	Passeriformes	Cardinalidae	5	10	17	3
Aves	Passeriformes	Chloropseidae	0	0	3	3
Aves	Passeriformes	Cisticolidae	6	3	0	0
Aves	Passeriformes	Colluricinclidae	3	1	0	1
Aves	Passeriformes	Corvidae	19	22	20	12
Aves	Passeriformes	Cotingidae	3	4	7	0
Aves	Passeriformes	Cracticidae	5	2	1	0
Aves	Passeriformes	Dasyornithidae	1	0	0	0
Aves	Passeriformes	Dendrocolaptidae	1	0	0	0
Aves	Passeriformes	Dicaeidae	2	1	9	9
Aves	Passeriformes	Dicruridae	2	0	0	1
Aves	Passeriformes	Emberizidae	8	96	32	9
Aves	Passeriformes	Estrildidae	6	11	3	4
Aves	Passeriformes	Eupetidae	2	0	0	0
Aves	Passeriformes	Eurylaimidae	2	0	0	0
Aves	Passeriformes	Falcunculidae	1	0	0	0
Aves	Passeriformes	Formicariidae	3	1	1	0
Aves	Passeriformes	Fringillidae	22	29	18	4
Aves	Passeriformes	Furnariidae	3	1	1	0
Aves	Passeriformes	Hirundinidae	2	2	0	0
Aves	Passeriformes	Icteridae	5	28	7	10
Aves	Passeriformes	Laniidae	3	2	0	0
Aves	Passeriformes	Malaconotidae	14	8	0	0
Aves	Passeriformes	Maluridae	1	0	3	0
Aves	Passeriformes	Melanocharitidae	1	0	0	0
Aves	Passeriformes	Meliphagidae	40	36	17	7
Aves	Passeriformes	Mimidae	3	5	10	5
Aves	Passeriformes	Monarchidae	5	1	0	0
Aves	Passeriformes	Motacillidae	5	0	0	0
Aves	Passeriformes	Muscicapidae	42	38	11	7
Aves	Passeriformes	Nectariniidae	3	11	23	0
Aves	Passeriformes	Oriolidae	0	0	2	6
Aves	Passeriformes	Orthonychidae	2	0	0	0
Aves	Passeriformes	Pachycephalidae	6	2	0	0
Aves	Passeriformes	Paradisaeidae	0	1	3	2
Aves	Passeriformes	Paridae	5	23	0	0
Aves	Passeriformes	Parulidae	16	16	2	1
Aves	Passeriformes	Passeridae	4	0	4	0
Aves	Passeriformes	Petroicidae	1	2	0	0
Aves	Passeriformes	Philepittidae	0	0	1	0
Aves	Passeriformes	Pipridae	1	2	1	0
Aves	Passeriformes	Pittidae	2	0	0	0
Aves	Passeriformes	Pityriaseidae	1	0	0	0
Aves	Passeriformes	Platysteiridae	0	1	0	0
Aves	Passeriformes	Ploceidae	16	12	5	2
Aves	Passeriformes	Pomatostomidae	2	0	0	0

Aves	Passeriformes	Prunellidae	3	3	0	0
Aves	Passeriformes	Ptilonorhynchidae	0	0	2	1
Aves	Passeriformes	Pycnonotidae	8	15	4	15
Aves	Passeriformes	Remizidae	1	2	0	1
Aves	Passeriformes	Rhabdornithidae	0	0	3	0
Aves	Passeriformes	Rhinocryptidae	2	0	0	0
Aves	Passeriformes	Sittidae	0	0	0	1
Aves	Passeriformes	Sturnidae	5	11	8	19
Aves	Passeriformes	Sylviidae	25	21	4	3
Aves	Passeriformes	Thamnophilidae	13	0	0	0
Aves	Passeriformes	Thraupidae	11	18	16	19
Aves	Passeriformes	Timaliidae	50	65	22	3
Aves	Passeriformes	Troglodytidae	4	7	0	0
Aves	Passeriformes	Turdidae	5	21	35	24
Aves	Passeriformes	Tyrannidae	46	49	24	10
Aves	Passeriformes	Vangidae	3	0	0	0
Aves	Passeriformes	Vireonidae	8	16	5	1
Aves	Passeriformes	Zosteropidae	2	13	44	19
Aves	Piciformes	Bucconidae	3	1	0	0
Aves	Piciformes	Indicatoridae	7	4	1	0
Aves	Piciformes	Picidae	38	31	8	4
Aves	Piciformes	Ramphastidae	0	2	4	7
Aves	Psittaciformes	Psittacidae	7	22	80	36
Aves	Rheiformes	Rheidae	0	1	0	0
Aves	Tinamiformes	Tinamidae	5	15	3	5
Aves	Trogoniformes	Trogonidae	4	3	3	3
Mammalia	Diprotodontia	Acrobatidae	0	0	0	1
Mammalia	Carnivora	Ailuridae	1	0	0	0
Mammalia	Rodentia	Anomaluridae	0	0	0	4
Mammalia	Primates	Aotidae	0	8	0	0
Mammalia	Primates	Atelidae	0	2	0	10
Mammalia	Cetartiodactyla	Bovidae	4	8	18	0
Mammalia	Diprotodontia	Burramyidae	0	4	1	0
Mammalia	Paucituberculata	Caenolestidae	5	0	0	0
Mammalia	Carnivora	Canidae	4	7	2	1
Mammalia	Rodentia	Capromyidae	0	0	20	0
Mammalia	Rodentia	Caviidae	2	0	3	0
Mammalia	Primates	Cebidae	0	9	31	6
Mammalia	Primates	Cercopithecidae	7	20	37	23
Mammalia	Cetartiodactyla	Cervidae	7	10	2	0
Mammalia	Primates	Cheirogaleidae	1	0	10	0
Mammalia	Rodentia	Cricetidae	74	133	76	5
Mammalia	Rodentia	Cuniculidae	0	2	0	0
Mammalia	Cingulata	Dasypodidae	0	2	0	0
Mammalia	Rodentia	Dasyproctidae	0	0	0	13
Mammalia	Dasyuromorphia	Dasyuridae	2	0	2	0

Mammalia	Didelphimorphia	Didelphidae	14	26	19	0
Mammalia	Rodentia	Dinomyidae	0	0	0	1
Mammalia	Rodentia	Dipodidae	8	2	13	0
Mammalia	Rodentia	Echimyidae	0	36	20	0
Mammalia	Proboscidea	Elephantidae	1	0	0	0
Mammalia	Chiroptera	Emballonuridae	8	1	0	0
Mammalia	Rodentia	Erethizontidae	1	0	14	1
Mammalia	Eulipotyphla	Erinaceidae	8	4	0	0
Mammalia	Carnivora	Eupleridae	2	1	0	0
Mammalia	Carnivora	Felidae	1	1	0	0
Mammalia	Primates	Galagidae	3	8	0	0
Mammalia	Rodentia	Gliridae	1	21	4	1
Mammalia	Carnivora	Herpestidae	13	3	1	0
Mammalia	Rodentia	Heteromyidae	0	19	0	0
Mammalia	Cetartiodactyla	Hippopotamidae	1	0	0	0
Mammalia	Primates	Hominidae	2	0	0	0
Mammalia	Carnivora	Hyaenidae	2	0	0	0
Mammalia	Diprotodontia	Hypsiprymnodontidae	0	1	0	0
Mammalia	Rodentia	Hystricidae	0	10	1	0
Mammalia	Primates	Indriidae	0	1	7	1
Mammalia	Primates	Lemuridae	1	1	5	1
Mammalia	Primates	Lorisidae	1	3	2	0
Mammalia	Diprotodontia	Macropodidae	4	5	0	0
Mammalia	Macroscelidea	Macroscelididae	1	0	0	0
Mammalia	Pilosa	Megalonychidae	0	0	2	0
Mammalia	Carnivora	Mephitidae	5	2	0	0
Mammalia	Rodentia	Muridae	86	146	40	30
Mammalia	Carnivora	Mustelidae	4	6	5	3
Mammalia	Rodentia	Nesomyidae	0	15	10	12
Mammalia	Rodentia	Octodontidae	0	4	2	3
Mammalia	Peramelemorphia	Peramelidae	0	0	0	7
Mammalia	Diprotodontia	Petauridae	4	0	0	0
Mammalia	Rodentia	Petromuridae	0	0	1	0
Mammalia	Diprotodontia	Phalangeridae	0	5	0	17
Mammalia	Chiroptera	Phyllostomidae	12	16	23	15
Mammalia	Primates	Pitheciidae	0	0	6	2
Mammalia	Rodentia	Platacanthomyidae	0	0	0	2
Mammalia	Hyracoidea	Procaviidae	0	0	2	0
Mammalia	Carnivora	Procyonidae	1	2	0	0
Mammalia	Diprotodontia	Pseudocheiridae	5	9	1	0
Mammalia	Chiroptera	Pteropodidae	3	2	3	7
Mammalia	Scandentia	Ptilocercidae	0	0	1	0
Mammalia	Perissodactyla	Rhinocerotidae	0	1	1	0
Mammalia	Rodentia	Sciuridae	1	68	64	24
Mammalia	Eulipotyphla	Solenodontidae	0	4	0	0
Mammalia	Rodentia	Spalacidae	0	0	3	0

Mammalia	Cetartiodactyla	Suidae	2	3	0	4
Mammalia	Cetartiodactyla	Tayassuidae	1	0	1	0
Mammalia	Afrosoricida	Tenrecidae	0	0	0	1
Mammalia	Rodentia	Thryonomyidae	2	0	0	0
Mammalia	Cetartiodactyla	Tragulidae	0	6	0	0
Mammalia	Scandentia	Tupaiidae	1	1	0	0
Mammalia	Carnivora	Ursidae	1	2	0	1
Mammalia	Carnivora	Viverridae	7	8	1	1

Table S6: List of botanical countries (assemblages), their respective continents, and the biogeographical realms used in the analyses. The definition of botanical countries and continents follows the Taxonomic Databases Working Group (TDWG; https://www.tdwg.org/), classification at level 3 and level 1, respectively (Govaerts *et al*., 2021). TDWG level 1 continents were assigned to biogeographical realms as follows: 'Africa' to 'Afrotropics'; 'Southern America' and 'Northern America' to 'Neotropics'; and 'Asia-Temperate', 'Asia-Tropical', 'Australasia' and 'Pacific' to 'Asia-Pacific'.

Botanical country code	Botanical country name	Continent	Realm
ANG	Angola	Africa	Africa
ASS	Assam	Asia-Tropical	Asia-Pacific
BAN	Bangladesh	Asia-Tropical	Asia-Pacific
BEN	Benin	Africa	Africa
BKN	Burkina	Africa	Africa
BLZ	Belize	Southern America	Americas
BOL	Bolivia	Southern America	Americas
BOR	Borneo	Asia-Tropical	Asia-Pacific
BOT	Botswana	Africa	Africa
BUR	Burundi	Africa	Africa
BZC	Brazil West-Central	Southern America	Americas
BZE	Brazil Northeast	Southern America	Americas
BZL	Brazil Southeast	Southern America	Americas
BZN	Brazil North	Southern America	Americas
BZS	Brazil South	Southern America	Americas
CAB	Cabinda	Africa	Africa
CAF	Central African Republic	Africa	Africa
CBD	Cambodia	Asia-Tropical	Asia-Pacific
CHA	Chad	Africa	Africa
CHC	China South-Central	Asia-Temperate	Asia-Pacific
CHH	Hainan	Asia-Temperate	Asia-Pacific
CHS	China Southeast	Asia-Temperate	Asia-Pacific
CLM	Colombia	Southern America	Americas
CMN	Cameroon	Africa	Africa
CON	Congo	Africa	Africa
COS	Costa Rica	Southern America	Americas
CUB	Cuba	Southern America	Americas
DOM	Dominican Republic	Southern America	Americas
ECU	Ecuador	Southern America	Americas
EHM	East Himalaya	Asia-Tropical	Asia-Pacific
ELS	El Salvador	Southern America	Americas
EQG	Equatorial Guinea	Africa	Africa
ETH	Ethiopia	Africa	Africa
FRG	French Guiana	Southern America	Americas

GAB	Gabon	Africa	Africa
GAM	Gambia, The	Africa	Africa
GGI	Gulf of Guinea Is.	Africa	Africa
GHA	Ghana	Africa	Africa
GNB	Guinea-Bissau	Africa	Africa
GUA	Guatemala	Southern America	Americas
GUI	Guinea	Africa	Africa
GUY	Guyana	Southern America	Americas
HAI	Haiti	Southern America	Americas
HON	Honduras	Southern America	Americas
IND	India	Asia-Tropical	Asia-Pacific
IVO	Ivory Coast	Africa	Africa
JAM	Jamaica	Southern America	Americas
JAW	Jawa	Asia-Tropical	Asia-Pacific
KEN	Kenya	Africa	Africa
LAO	Laos	Asia-Tropical	Asia-Pacific
LBR	Liberia	Africa	Africa
LSI	Lesser Sunda Is.	Asia-Tropical	Asia-Pacific
MDG	Madagascar	Africa	Africa
MLI	Mali	Africa	Africa
MLW	Malawi	Africa	Africa
MLY	Malaya	Asia-Tropical	Asia-Pacific
MOL	Maluku	Asia-Tropical	Asia-Pacific
MOZ	Mozambique	Africa	Africa
MXC	Mexico Central	Northern America	Americas
MXE	Mexico Northeast	Northern America	Americas
MXG	Mexico Gulf	Northern America	Americas
MXN	Mexico Northwest	Northern America	Americas
MXS	Mexico Southwest	Northern America	Americas
MXT	Mexico Southeast	Northern America	Americas
MYA	Myanmar	Asia-Tropical	Asia-Pacific
NAT	KwaZulu-Natal	Africa	Africa
NEP	Nepal	Asia-Tropical	Asia-Pacific
NGA	Nigeria	Africa	Africa
NIC	Nicaragua	Southern America	Americas
NTA	Northern Territory	Australasia	Asia-Pacific
NWC	New Caledonia	Pacific	Asia-Pacific
NWG	New Guinea	Asia-Tropical	Asia-Pacific
PAN	Panama	Southern America	Americas
PAR	Paraguay	Southern America	Americas
PER	Peru	Southern America	Americas
PHI	Philippines	Asia-Tropical	Asia-Pacific
QLD	Queensland	Australasia	Asia-Pacific
RWA	Rwanda	Africa	Africa
SEN	Senegal	Africa	Africa
SIE	Sierra Leone	Africa	Africa

SOL	Solomon Is.	Asia-Tropical	Asia-Pacific
SOM	Somalia	Africa	Africa
SRL	Sri Lanka	Asia-Tropical	Asia-Pacific
SUD	Sudan	Africa	Africa
SUL	Sulawesi	Asia-Tropical	Asia-Pacific
SUM	Sumatera	Asia-Tropical	Asia-Pacific
SUR	Surinam	Southern America	Americas
SWZ	Swaziland	Africa	Africa
TAN	Tanzania	Africa	Africa
THA	Thailand	Asia-Tropical	Asia-Pacific
TOG	Togo	Africa	Africa
TRT	Trinidad-Tobago	Southern America	Americas
TVL	Northern Provinces	Africa	Africa
UGA	Uganda	Africa	Africa
VAN	Vanuatu	Pacific	Asia-Pacific
VEN	Venezuela	Southern America	Americas
VIE	Vietnam	Asia-Tropical	Asia-Pacific
WIN	Windward Is.	Southern America	Americas
ZAI	Zaïre	Africa	Africa
ZAM	Zambia	Africa	Africa
ZIM	Zimbabwe	Africa	Africa
AND	Andaman Is.	Asia-Tropical	Asia-Pacific
BAH	Bahamas	Southern America	Americas
FLA	Florida	Northern America	Americas
LEE	Leeward Is. AB Ant	Southern America	Americas
NAM	Namibia	Africa	Africa
NCB	Nicobar Is.	Asia-Tropical	Asia-Pacific
PUE	Puerto Rico	Southern America	Americas
TAI	Taiwan	Asia-Temperate	Asia-Pacific

Table S7: Results for spatial autoregressive (SAR_{err}) model. This approach takes into account the spatial neighbourhood of data points (botanical countries, n=101) to evaluate the effects of direct predictors that explain Annonaceae FRic and Annonaceae SRic across assemblages globally. Relationships were derived from the structural equation model assessing direct and indirect effects on Annonaceae FRic across botanical countries, while SAR_{err} models were analysed separately for the equations with Annonaceae FRic or SRic as the response variable.

**Response**	**Predictor**	**Estimate**	**Std. Error**	**z value**	**Pr(>|z|)**
Annonaceae FRic	Annonaceae SRic	0.623535	0.059141	10.5431	< 2.2e-16
Annonaceae FRic	Mammal FRic	0.119213	0.044263	2.6933	0.007075
Annonaceae SRic	Mammal SRic	0.646452	0.070503	9.1692	< 2.2e-16
Annonaceae SRic	Area size	0.280845	0.073922	3.7992	0.0001452
Annonaceae SRic	Annual precipitation	0.187328	0.070327	2.6637	0.0077291

Table S8: Frugivory-related trait matching between Annonaceae and mammalian frugivores based on co-occurrence across botanical countries at a global scale. Continuous variables were log-transformed and re-scaled between zero and one. “Stat” indicates the fourthcorner function for which the fourth link was measured depending on the variable types (i.e., by a Pearson correlation coefficient for two quantitative variables, by a Pearson Chi2 and G statistic for two qualitative variables and by a Pseudo-F and Pearson r for one quantitative variable and one qualitative variable). “Std.Obs” represents the standardized effect size, which is computed as the observed statistic centered and scaled; means and standard deviations for standardization are computed from the distribution of the statistic under the null hypothesis (i.e., on the permuted data). “Alter” refers to the alternative hypothesis being tested, indicating whether the test is one-sided (greater or less) or two-sided. “Adj. P-value” indicates the p-value adjusted for multiple testing. Signif. Symbol: 0 ‘***’ 0.001 ‘**’ 0.01 ‘*’ 0.05 ‘.’ 0.1 ‘ ’ 1.

Annonaceae trait	Mammal trait	Stat	Obs	Std.Obs	Alter	Pvalue	Pvalue.adj	Symbol
**Fruit length (cm)**	**Adult body mass (g)**	**r**	**0.05357992**	**4.2351499**	**two-sided**	**0.000999**	**0.0053280**	**
**Tree**	**Adult body mass (g)**	**F**	**78.76508273**	**8.1329539**	**greater**	**0.001998**	**0.0063936**	**
Shrub	Adult body mass (g)	F	0.28235573	-0.7242998	greater	0.879121	0.9420579	
**Liana**	**Adult body mass (g)**	**F**	**772.78991906**	**19.0753378**	**greater**	**0.000999**	**0.0053280**	**
Fruit length (cm)	Arboreal foraging stratum	F	1.79772720	-0.5917795	greater	0.673327	0.9420579	
Tree	Arboreal foraging stratum	Chi2	2.42417082	-0.4837595	greater	0.585415	0.9366633	
Shrub	Arboreal foraging stratum	Chi2	4.89645336	-0.3014499	greater	0.434565	0.9366633	
Liana	Arboreal foraging stratum	Chi2	37.18671611	0.2071109	greater	0.270729	0.8663337	
Fruit length (cm)	Ground level foraging stratum	F	2.91633636	-0.4694513	greater	0.576424	0.9366633	
Tree	Ground level foraging stratum	Chi2	2.42114669	-0.4638160	greater	0.583417	0.9366633	

Shrub	Ground level foraging stratum	Chi2	5.50316505	-0.2410786	greater	0.413586	0.9366633	
Liana	Ground level foraging stratum	Chi2	62.28862548	0.7696788	greater	0.137862	0.5514486	
Fruit length (cm)	Scansorial foraging stratum	F	0.08173438	-0.6844213	greater	0.928072	0.9420579	
Tree	Scansorial foraging stratum	Chi2	0.10038606	-0.7243088	greater	0.914086	0.9420579	
Shrub	Scansorial foraging stratum	Chi2	0.06051107	-0.7401172	greater	0.942058	0.9420579	
Liana	Scansorial foraging stratum	Chi2	2.22264394	-0.6066742	greater	0.767233	0.9420579	

Supplementary References

Brown CM, Arbour JH, Jackson DA. 2012. Testing of the effect of missing data estimation and distribution in morphometric multivariate data analyses. *Systematic Biology* **61(6)**: 941–954.

Carbone C, Cowlishaw G, Isaac N, Rowcliffe JM. 2005. How far do animals go? Determinants of day range in mammals. *The American Naturalist* **165**: 290–297.

Chen SC, Moles AT. 2015. A mammoth mouthful? A test of the idea that larger animals ingest larger seeds. *Global Ecology and Biogeography* **24(11)**: 1269–1280.

Claramunt S. 2021. Flight efficiency explains differences in natal dispersal distances in birds. *Ecology* **102**: e03442.

Coelho MTP, Barreto E, Rangel TF, Diniz-Filho JAF, Wüest RO, Bach W, Skeels A, McFadden IR, Roberts DW, Pellissier L et al. 2023. The geography of climate and the global patterns of species diversity. *Nature* **622**: 537–544.

Dagallier L. 2021. *Diversification of the tropical African flora: spatial and temporal approaches.* Doctoral dissertation. Montpellier, France: Université Montpellier.

Damthongdee A, Aongyong K, Chaowasku T. 2021. *Orophea sichaikhan*ii (Annonaceae), a new species from southern Thailand, with a key to the species of *Orophea* in Thailand and notes on some species. *Plant Ecology and Evolution* **154(2)**: 307–315.

Erkens RH, Blanpain LM, Jara IC, Runge K, Verspagen N, Cosiaux A, Couvreur TL. 2022. Spatial distribution of Annonaceae across biomes and anthromes: Knowledge gaps in spatial and ecological data. Plants, People, Planet **5(4)**: 520–535.

Fricke EC, Svenning JC. 2020. Accelerating homogenization of the global plant–frugivore meta-network. *Nature* **585(7823)**: 74–78.

GBIF. 2022. GBIF.org *GBIF Occurrence Download* [WWW document] URL https://doi.org/10.15468/dl.ddz83q [accessed 18 April 2022].

Givnish TJ. 2010. Ecology of plant speciation. *Taxon* **59**: 1326–1366.

Govaerts R, Nic Lughadha E, Black N, Turner R, Paton A. 2021. The World Checklist of Vascular Plants, a continuously updated resource for exploring global plant diversity. *Scientific Data* **8**: 1–10.

Hijmans RJ, Bivand R, Forner K, Ooms J, Pebesma E. 2022. 'terra': Spatial data analysis. R package v.1.7-3. [WWW document] URL https://CRAN.R-project.org/package=terra [accessed 06 Mai 2022].

Hoekstra PH, Wieringa JJ, Maas PJM, Chatrou LW. 2021. Revision of the African species of *Monanthotaxis* (Annonaceae). *Blumea-Biodiversity, Evolution and Biogeography of Plants*, **66(2)**: 107–221.

Jordano P. 2000. Fruits and frugivory. In: Fenner M, ed. *Seeds: The ecology of regeneration in plant communities.* Wallingford, UK: CABI, 125–166, vol. 2.

Kral R. 1960. A revision of *Asimina* and *Deeringothamnus* (Annonaceae). *Brittonia* **12(4)**: 233–278.

Leeratiwong C, Chalermglin P, Johnson DM. 2021a. *Alphonsea annulata* (Annonaceae), a new species from Thailand. *Kew Bulletin* **76(2)**: 309–315.

Leeratiwong C, Chalermglin P, Saunders RM. 2021b. *Goniothalamus roseipetalus* and *G. sukhirinensis* (Annonaceae): Two new species from Peninsular Thailand. *PhytoKeys* **184**: 1–17.

Lim JY, Svenning JC, Göldel B, Faurby S, Kissling WD. 2020. Frugivore-fruit size relationships between palms and mammals reveal past and future defaunation impacts. *Nature Communications* **11**: 1–13.

Maas PJ, Westra LYT. 2003. Revision of the neotropical genus *Pseudoxandra* (Annonaceae). *Blumea-Biodiversity, Evolution and Biogeography of Plants* **48(2)**: 201–259.

Maas PJ, Westra LY, Vermeer M. 2007. Revision of the neotropical genera *Bocageopsis*, *Onychopetalum*, and *Unonopsis* (Annonaceae). *Blumea-Biodiversity, Evolution and Biogeography of Plants* **52(3)**: 413–554.

Maas PJM, Westra LYT, Guerrero SA, Lobão AQ, Scharf U, Zamora NA, Erkens RHJ. 2015. Confronting a morphological nightmare: revision of the Neotropical genus *Guatteria* (Annonaceae). *Blumea-Biodiversity, Evolution and Biogeography of Plants* **60(1-2)**: 1–219.

Marshall AR, Couvreur TL, Summers AL, Deere NJ, Luke WQ, Ndangalasi HJ, Sparrow S, Johnson DM. 2016. A new species in the tree genus *Polyceratocarpus* (Annonaceae) from the Udzungwa Mountains of Tanzania. *PhytoKeys* **63**: 63–76.

McFadden IR, Fritz SA, Zimmermann NE, Pellissier L, Kissling WD, Tobias JA, Schleuning M, Graham CH. 2022. Global plant-frugivore trait matching is shaped by climate and biogeographic history. *Ecology Letters* **25(3)**: 686–696.

Mello-Silva R, Lopes JC. 2020. The Brazilian Atlantic Forest genus *Bocagea* (Annonaceae), *Phytotaxa* **475(4)**: 279–288.

Mello-Silva R, Lopes JDC, Johnson DM. 2021. The new inland *Hornschuchia mediterranea* (Annonaceae) from Bahia, Brazil. *Phytotaxa* **483(3)**: 285–290.

Onstein RE, Baker WJ, Couvreur TLP, Faurby S, Svenning JC, Kissling WD. 2017. Frugivory-related traits promote speciation of tropical palms. *Nature Ecology & Evolution* **1**: 1903–1911.

Pires MM, Guimarães PR, Galetti M, Jordano P. 2018. Pleistocene megafaunal extinctions and the functional loss of long-distance seed-dispersal services. *Ecography* **41**: 153–163.

Stekhoven DJ, Bühlmann P. 2012. MissForest—non-parametric missing value imputation for mixed-type data. *Bioinformatics* **28**: 112–118.

Su YCF, Saunders RMK. 2006. Monograph of *Pseuduvaria* (Annonaceae). *Systematic Botany Monographs* **79**: 1–204.

Thiel S, Willems F, Farwig N, Rehling F, Schabo DG, Schleuning M, Tello NS, Töpfer T, Tschapka M, Heymann EW, Heer K. 2023. Vertically stratified frugivore community composition and interaction frequency in a liana fruiting across forest strata. *Biotropica* **55(3)**: 650–664.

Thomson FJ, Moles AT, Auld TD, Kingsford RT. 2011. Seed dispersal distance is more strongly correlated with plant height than with seed mass. *Journal of Ecology* **99**: 1299–1307.

van Setten AK, Koek-Noorman J, Schipper T. 1992. Fruits and seeds of Annonaceae: Morphology and its significance for classification. Stuttgart, Germany: Bibliotheca Botanica.

Wheelwright NT. 1985. Fruit-size, gape width, and the diets of fruit-eating birds. *Ecology* **66(3)**: 808–818.

Wilman H, Belmaker J, Simpson J, de la Rosa C, Rivadeneira MM, Jetz W. 2014. EltonTraits 1.0: Species-level foraging attributes of the world's birds and mammals: Ecological Archives E095-178. *Ecology* **95(7)**: 2027–2027.

Wiya C, Aongyong K, Damthongdee A, Baka A, Chaowasku T. 2021. The genus *Phaeanthus* (Annonaceae, Miliuseae) in Thailand: *P. piyae* sp. nov. and resurrection of *P. lucidus*, with molecular phylogenetic analyses. *Taiwania* **66(4)**: 509–516.

Wölke F, Cabral A, Lim JY, Kissling WD, Onstein RE. 2023. Africa as the evolutionary arena for large fruits. *New Phytologist* **special issue**: 1–13.

Xue B, Shao YY, Xiao CF, Liu MF, Li Y, Tan YH. 2021a. *Meiogyne oligocarpa* (Annonaceae), a new species from Yunnan, China. *PeerJ* **9**: e10999.

Xue B, Li JW, Liao JJ, Mo MZ, Tan YH, Chen YS. 2021b. *Trivalvaria tomentosa* (Annonaceae), a new species from Southeast Yunnan, China. *Nordic Journal of Botany* **39(8)**: 1–8.

Yoosukkee C, Damthongdee A, Jongsook H, Chaowasku T. 2020. *Pseuduvaria khaosokensis* (Annonaceae), a new species from southern Thailand as evidenced by plastid phylogeny and morphology. In: *Annales Botanici Fennici* **58(1–3)**: 49–59. Helsinki, Finland: Finnish Zoological and Botanical Publishing Board.
